# Fungal and Prokaryotic Activities in the Marine Subsurface Biosphere at Peru Margin and Canterbury Basin Inferred from RNA-Based Analyses and Microscopy

**DOI:** 10.3389/fmicb.2016.00846

**Published:** 2016-06-09

**Authors:** Maria G. Pachiadaki, Vanessa Rédou, David J. Beaudoin, Gaëtan Burgaud, Virginia P. Edgcomb

**Affiliations:** ^1^Department of Geology and Geophysics, Woods Hole Oceanographic InstitutionWoods Hole, MA, USA; ^2^Laboratoire Universitaire de Biodiversité et Ecologie Microbienne, EA 3882, ESIAB, Technopôle de Brest Iroise, Université de BrestPlouzané, France; ^3^Department of Biology, Woods Hole Oceanographic InstitutionWoods Hole, MA, USA

**Keywords:** metatranscriptome, iTAG, fungi, calcofluor white, sediment, rRNA operon, mRNA, DNA

## Abstract

The deep sedimentary biosphere, extending 100s of meters below the seafloor harbors unexpected diversity of Bacteria, Archaea, and microbial eukaryotes. Far less is known about microbial eukaryotes in subsurface habitats, albeit several studies have indicated that fungi dominate microbial eukaryotic communities and fungal molecular signatures (of both yeasts and filamentous forms) have been detected in samples as deep as 1740 mbsf. Here, we compare and contrast fungal ribosomal RNA gene signatures and whole community metatranscriptomes present in sediment core samples from 6 and 95 mbsf from Peru Margin site 1229A and from samples from 12 and 345 mbsf from Canterbury Basin site U1352. The metatranscriptome analyses reveal higher relative expression of amino acid and peptide transporters in the less nutrient rich Canterbury Basin sediments compared to the nutrient rich Peru Margin, and higher expression of motility genes in the Peru Margin samples. Higher expression of genes associated with metals transporters and antibiotic resistance and production was detected in Canterbury Basin sediments. A poly-A focused metatranscriptome produced for the Canterbury Basin sample from 345 mbsf provides further evidence for active fungal communities in the subsurface in the form of fungal-associated transcripts for metabolic and cellular processes, cell and membrane functions, and catalytic activities. Fungal communities at comparable depths at the two geographically separated locations appear dominated by distinct taxa. Differences in taxonomic composition and expression of genes associated with particular metabolic activities may be a function of sediment organic content as well as oceanic province. Microscopic analysis of Canterbury Basin sediment samples from 4 and 403 mbsf produced visualizations of septate fungal filaments, branching fungi, conidiogenesis, and spores. These images provide another important line of evidence supporting the occurrence and activity of fungi in the deep subseafloor biosphere.

## Introduction

The marine deep subsurface biosphere includes both sedimentary and oceanic crustal habitats hydrated by subsurface fluid flows. Based on compilations of counts of cells (primarily of Bacteria and Archaea) in marine sediments from locations that include the South and North Pacific Gyres and the eastern equatorial Pacific Ocean and ocean margins, the marine subsurface biosphere is thought to host numbers of cells that rival those in surface environments (Kallmeyer et al., 2012). Total prokaryotic cell abundance in marine sediments was recently estimated to be 5.39 × 10^29^ cells, which is comparable to global estimates of cell numbers in seawater and soil (Parkes et al., 2014). Although this accounts for only 0.18–3.6% of total global biomass (Kallmeyer et al., 2012), given the physical extent of potential sedimentary and rock subsurface habitats, contributions to global elemental cycling may be non-trivial in locations where sufficient carbon, electron donors and acceptors, and other nutrient sources exist (reviewed by Orcutt et al., 2013). Microorganisms in subsurface habitats confront increased challenges with increasing depth below seafloor. These include increased pressure, elevated temperatures, more refractory organic carbon pools, pH gradients resulting from fluid flow through sediments and rocks with different mineralogy, and in some habitats, decreased pore spaces. The marine subsurface is overall, a very heterogeneous amalgam of habitats more or less conducive to microbial life, with gradients that can vary on the sub millimeter scale (Schrenk et al., 2010).

The majority of subsurface microorganisms are thought to live under conditions of such extreme energy limitation that generation times may be up to 1000s of years (Jørgensen and Boetius, 2007). Nonetheless, at 1,000 m below seafloor (mbsf), the mean cell number of bacteria and archaea in sediments reaches up to 10^6^ cells per cm^3^ (Jørgensen and Boetius, 2007). Except for zones of particularly organic-rich deposits, heterotrophic populations must survive on increasingly recalcitrant (low reactivity) and increasingly diminished concentrations of organic material that has escaped remineralization by other microorganisms during the process of seafloor deposition and subsequent burial (Burdige, 2007). Even if necromass from (micro)eukaryotes and autotrophic/heterotrophic prokaryotes is a significant component of useable energy and carbon in the buried subsurface, the most labile molecules are rapidly removed after burial. Gas hydrates, organic rich layers, and sulfate/methane interfaces may be hotspots of activity that provide new pools of labile organic molecules. Additionally, buried organic material may be reactivated at depth at thermogenic temperatures that release methane, hydrocarbons, acetate, hydrogen, and CO_2_ that can diffuse upward to help fuel the deep biosphere (Parkes et al., 2014). Subsurface microbiota in the majority of locations, however, must have adaptations for survival in energy- and carbon-limited habitats. Culture-based studies have indeed demonstrated the ability of subsurface prokaryotic isolates to survive on extremely low energy fluxes (Hoehler and Jørgensen, 2013).

Microbial diversity in subsurface sediments includes representatives from all three domains of life. A meta-analysis of published prokaryotic small subunit ribosomal RNA gene (SSU rDNA) datasets from a range of subsurface sediments and depths shows that novel Archaea and Bacteria within the *Chloroflexi, Gammaproteobacteria, Planctomycetes*, and *Atribacteria* (the former candidate phylum JSI) dominate (Parkes et al., 2014). Subsurface environments are providing discoveries of new taxonomic groups, such as the recently described ‘Hadesarchaea’ discovered deep below hotsprings in Yellowstone National Park (Baker et al., 2016). Parkes et al. (2014) found overall prokaryotic community composition appears to be linked to sediment type or oceanic province. Although archaea have dominated the snapshot of subsurface populations obtained from some samples using molecular techniques on bulk extracted DNA (e.g., Biddle et al., 2008), it is premature at this point to generalize on whether bacterial or archaeal populations dominate or are more active. A metatranscriptome study of Peru Margin sediments from 5 to 159 mbsf showed that bacterial transcripts dominated, with most transcripts coming from *Firmicutes, Actinobacteria, Alphaproteobacteria*, and *Gammaproteobacteria* (Orsi et al., 2013a). The heterogeneity of detected metabolic lifestyles is thought to be linked to the severe energy limitation encountered in the deep subsurface, which prevents any one metabolism/prokaryotic population from dominating (Parkes et al., 2014).

Culture-independent approaches targeting microeukaryotes suggest their presence and activity in the deep subsurface on the basis of DNA, rRNA, and mRNA (Edgcomb et al., 2011; Orsi et al., 2013b; Ciobanu et al., 2014), with fungal sequences dominating the fraction of microeukaryote signatures. A recent culture-based study produced the isolation of numerous fungal isolates from different deep subseafloor sediment depths at Canterbury Basin (Rédou et al., 2015). That study examined the same sediment cores analyzed here. All of the fungal isolates obtained are known cosmopolitan species, raising intriguing ecological questions regarding their origin and abilities to adapt to deep subsurface conditions. Eco-physiological analyses exploring growth at different temperatures and in different salinities revealed that fungal strains obtained from the greatest depths are much better adapted to conditions resembling their *in situ* habitat compared to the same species isolated from shallower depths. Such a physiological shift from apparently terrestrial-adapted to marine-adapted lifestyles along the sediment core may indicate a transition where fungi become increasingly better adapted to subsurface conditions. While these results provide intriguing insights into the possible adaptations possessed by subsurface fungi, micro-eukaryotic cells are not abundant in the energy-starved subsurface sediments compared to prokaryotes. Fossilized microbial consortia of fungi and prokaryotes have been visualized in subseafloor igneous crust (Ivarsson et al., 2013, 2015; Bengston et al., 2014), suggesting symbiosis between chemoautotrophic prokaryotes and heterotrophic fungi in deep crustal environments. However, until now, non-fossilized fungal cells have not been visually detected in the deep subseafloor (Jørgensen and Marshall, 2016).

Our primary aim was to conduct an in-depth fungal-focused investigation of the occurrence, activity, and metabolic functions of deep subseafloor Fungi in sediment core samples from the Canterbury basin site U1352 and Peru Margin site 1229A. Peru Margin site 1229A is unusual in the sense that it has a deep brine incursion that peaks around 75 mbsf, so sulfate is present both near the sediment surface and at depth (Parkes et al., 2005). Organic matter content is 2–8% at this site, resulting in active prokaryotic communities at depth, as measured by cell counts, thymidine incorporation rates and rates of methanogenesis in a horizon at 95 mbsf that exceed rates at the sediment surface, and observed overlap of sulfate reduction and methanogenesis (Parkes et al., 2005). Canterbury Basin site U1352 is characterized by lithologies that span from unconsolidated sediments (clay, marl) to carbonate rocks (Ciobanu et al., 2014). Samples analyzed for this study consisted primarily of mud-rich sediment with some marl. Organic carbon content in the Canterbury Basin samples we analyzed was lower than in the Peru Margin samples, ranging from <0.6 to 1% (Ciobanu et al., 2014). In this study, we provide additional important evidence for active Fungi in the deep subsurface on the basis of iTAG analyses, metatranscriptomics, and microscopy. Fungal iTAG datasets were published previously for similar Peru Margin depths but were obtained using a broad microeukaryote-targeting approach (Orsi et al., 2013b). Here we aimed to apply fungal-specific PCR primers for iTAG studies to gather more in-depth information on this portion of the community. Microbial communities function as a consortium and exhibit complex interdependencies and interactions. Subseafloor Fungi must interact with prokaryotic community members present in these habitats (including competition for resources and responses to local biogeochemical conditions shaped by prokaryotes). Therefore, data on prokaryotic activities inferred from mRNA transcripts are presented, as they provide context for the habitat in which we investigate subsurface fungal populations. A eukaryote-focused metatranscriptome was also successful for one of our Canterbury Basin samples, which provides more in-depth information on eukaryotic activities in that sample and in particular, on the Fungi.

## Materials and Methods

### Site Descriptions and Sediment Core Collection

Sediment core samples were collected during IODP leg 201 from the continental margin of Peru at site 1229A (10°58.5721′S 77°57.4590′W) in 150.5 m water depth. Core depth at 1229A was 187 mbsf and the core lithology is described in the Shipboard Scientific Party (2003) report. For this work, we analyzed samples collected at 6 mbsf (2H2) and 95 mbsf (11H5). Samples preserved at -80°C were obtained from A. Teske (UNC Chapel Hill) for our analyses. Sediment samples were also collected at the Canterbury Basin, on the eastern margin of the South Island of New Zealand, during IODP leg 317 expedition (JOIDES Resolution). Sediment core was drilled at Site U1352 (44°56.2662 S 172°1.3630 E) in 344 m water depth. A total depth of 1927.5 mbsf was cored, spanning the Holocene to late Eocene periods. For this work, we obtained core sections from this site (1H3 from 3.76 mbsf, 2H5 from 11.60 mbsf, 4H1 from 24.60 mbsf, 42X3 from 345.50 mbsf and 48X3 from 403.10 mbsf) from M-C Ciobanu and K. Alain (University of Brest, France) that had been preserved at -80°C. The core lithology is described in Ciobanu et al. (2014).

### DNA Extraction, Purification, and Amplification for Peru Margin iTAG Analyses

Fungal iTAG analyses were performed only on Peru Margin sediment core samples as fungal iTAGs were generated and analyzed for the Canterbury Basin samples in a previous study (Rédou et al., 2014). DNA was extracted from either 2 or 20 g of exterior and interior core sediment from frozen (stored at -80°C) Peru Margin samples collected at 6 mbsf (2H2) and 95 mbsf (11H5), respectively, using the PowerSoil DNA Isolation Kit (MoBio Laboratories, USA). Extractions were also performed for replicate samples collected from the exterior of both cores. The manufacturer’s protocol was modified to include five repetitions of homogenization for 1-min intervals, with 1 min rest in between, using a FastPrep benchtop homogenizer (MP Biomedicals, Santa Ana, CA, USA) set to 4.0 m/s. A final purification step using isopropanol precipitation was also added. Duplicate extractions were performed for both interior and exterior regions of each core.

Partial small-subunit ribosomal DNA (SSU rDNA) fragments were PCR amplified from DNA extracts using the key-tagged fungal-targeting primer set nu-SSU-0817-5′ and nu-SSU-1196-3′ (Borneman and Hartin, 2000). Replicate PCR amplifications (3–6) were run for each sample using Phusion High-Fidelity DNA Polymerase (Thermo Fisher Scientific, USA) and 5X Phusion HF Buffer. PCR conditions were: 98°C for 30 s followed by 40 cycles of 98°C for 10 s, 56°C for 30 s, and 72°C for 30 s, and a final incubation for 7 min at 72°C. PCR products were visualized by agarose gel electrophoresis and positive results were excised and purified from the gel using the ZymoClean Gel DNA recovery Kit (Zymo Research, USA). Purified replicate PCR amplifications from each sample were combined prior to iTAG sequencing using Illumina MiSeq PE300 at Georgia Genomics Center. The Peru Margin iTAG sequence data are deposited at in the GenBank SRA (accession number SRP072127).

### Analyses of Peru Margin iTAG Sequence Data

The R1 and R2 iTAG sequences for Peru Margin were quality controlled using the Trim Galore! version 0.3.7^1^. Default values were applied. The R1 and R2 reads that survived quality trimming were merged using FLASh (Magoc and Salzberg, 2011). Sequence clustering at 97% similarity level was performed using uclust (Edgar, 2010) in QIIME pipeline (Caporaso et al., 2010). The taxonomic assignment of the representative sequences of each cluster was performed using BLASTN (Altschul et al., 1990) against Silva 111 (Quast et al., 2013). A heat map was generated for the two replicates of the two Peru Margin samples using the heat map function included in the OMICS module of XLStat (Addinsoft). Normalized read abundances were used for clustering of samples applying hierarchical clustering based on Euclidian distances.

### RNA Extraction, Purification, and Reverse Transcription for Metatranscriptome Analyses

RNA was extracted from 16 g of sediment from the Canterbury core samples and from 20 g of sediment from the Peru Margin core samples using the RNA PowerSoil Total RNA Isolation Kit (MoBio Laboratories, Carlsbad, CA, USA) following a modified manufacturer’s protocol. Modifications included: 10 cycles of homogenization for 1-min intervals with 1 min rest between intervals using a FastPrep benchtop homogenizer (MP Biomedicals, Santa Ana, CA, USA) set to 4.0 m/s and increasing the incubation time to 45 min following the addition of SR4 buffer. Trace DNA was removed by treatment with Turbo DNA-free (Life Technologies, Grand Island, NY, USA) for 60 min at 37°C. A final RNA purification step was performed using the MEGAclear kit (Life Technologies, USA). In order to avoid contamination, all manipulations were carried out in a dedicated PCR hood (AirClean Systems, USA) for RNA work. An extraction blank was also carried through the entire procedure to control for kit contamination and served as “negative control.” Removal of carry-over DNA in RNA extracts was confirmed by the absence of visible amplification of the V4 hypervariable region of SSU rDNA after 35 cycles of PCR using the RNA extracts as template with key-tagged bacterial primers (Flores et al., 2011). PCR conditions were: 95°C for 2 min followed by 35 cycles of 95°C for 15 s, 53°C for 45 s and 68°C for 45 s with a final incubation of 68°C for 3 min.

Total RNA was used as template for cDNA amplification using the Ovation 3′-DGE System (NuGEN, San Carlos, CA, USA) to obtain an enriched eukaryotic metatranscriptome through selection of poly-A transcripts. Double-stranded cDNA was purified using the DNA Clean & Concentrator kit (Zymo Research) as described in the modified user guide^2^ provided by NuGEN. The quantity of amplified cDNA was evaluated using a fluorometer (Qubit 2.0, Life Technologies).

The same RNA extract (5 μl) was used to construct metatranscriptomic libraries targeting all three domains of life using the Ovation RNA-Seq System V2 kit (NuGEN, San Carlos, CA, USA) that uses random hexamers to initiate reverse transcription. Purification of double stranded cDNA was performed with Agencourt RNAClean XP Purification Beads following the instructions provided by NuGEN. Two replicate extractions and reverse transcriptions were performed per sample.

### Illumina Library Preparation and Sequencing

For the Canterbury poly-A-enriched cDNA library preparations, paired-end 2 × 150 bp Illumina Hi-Seq sequencing was performed at the University of Delaware Sequencing and Genotyping Center (Delaware Biotechnology Institute). The Peru Margin and Canterbury Basin metatranscriptome library preparations that were successful (replicate RNA-Seq libraries for Peru Margin 6 and 95 mbsf, and replicate RNA-seq libraries for Canterbury Basin 12 and 345 mbsf, as well as a single poly-A-based library for Canterbury Basin 345 mbsf) were sent to Georgia Genomics Facility^3^ for library preparations and paired-end 2 × 150 Illumina NextSeq sequencing. The metatranscriptome sequence data are deposited in the GenBank SRA accession number SRP072032 (Canterbury Basin poly-A-based library) and accession number SRP072233 (Canterbury Basin and Peru Margin RNA-seq data).

### Analyses of Peru Margin and Canterbury Basin Metatranscriptome Data

The R1 and R2 reads were filtered using Trimmomatic (Bolger et al., 2014), which performs a “sliding window” trimming, cutting once the average quality within the window (eight nucleotides used here) falls below a threshold (set to 12). The length of the trimmed sequences was set to be at least 50 nucleotides. The trimmed reads surviving quality control were assembled into contigs using Trinity (Grabherr et al., 2011) release r20140717. Metapathways 2.5 (Konwar et al., 2013, 2015) was used for open reading frame predictions using Prodigal (Hyatt et al., 2010). Functional annotation of the contigs used LAST (Kiełbasa et al., 2011) against the RefSeq non-redundant database (Pruitt et al., 2005) update 2014-01-18, and visualization of metabolic pathways used Pathway Tools (Karp et al., 2010). Additionally for the poly-A-enriched libraries BLASTX analyses were run through the Blast2Go platform (Conesa et al., 2005) using an *e*-value of 10^-3^ and fungal taxonomy filter (fungi nr subset database). Contigs were then mapped and annotated to clusters of gene ontologies (GO) using an *e*-value of 10^-6^ and fungal taxonomy filter (fungi nr subset database). For the taxonomic annotation of the contigs BLASTX (*e*-value10^-3^) against nr and MEGAN v.5 10 7 were used.

To estimate the abundance of each transcript within a library a Burrows–Wheeler Aligner (BWA-based) version of the RPKM calculation (Li and Durbin, 2010) was applied as described in Konwar et al. (2015). RPKM calculates the proportion of number of reads mapped to a sequence section normalized for sequencing depth and transcript length. Transcripts that showed at least five times higher abundance in one of the sediment libraries compared to the control were retained for subsequent analyses discussed below.

### Visualization of Fungal Cells in Canterbury Basin Core Samples

Sediment samples (2.0 g) from the same core material that was immediately frozen at -80°C on the ship for shore-based molecular work were slowly thawed and then immediately fixed in 4% paraformaldehyde (final concentration) for 3 h at 4°C in the dark. We could not utilize the exact same sediment samples for microscopy as were used for extraction of nucleic acids because very little un-manipulated interior sediment remained from those samples that we could guarantee had not been exposed to potentially contaminating core exterior material. Additionally, the thawing and re-freezing of those samples may have damaged cellular structures. For this reason, we performed microscopy on frozen material from the same cores from slightly different depths, that had not been utilized previously for another purpose. After fixation, sediment samples were washed three times with sterile 1X phosphate buffered saline (PBS) and stained with Calcofluor white for 30 min in the dark (Damare and Raghukumar, 2008). Sediment samples were washed with sterile water and dropped on a coated PTFE/Teflon multi-well microscope slides at several dilutions (1:2, 1:5, 1:10, 1:50, and 1:100). Observations were processed using an epifluorescence microscope (Olympus).

## Results and Discussion

### Comparison of Overall Community Metabolism in Peru Margin and Canterbury Basin Samples

Metatranscriptomics have been widely used in microbial ecology as a proxy for microbial activity (e.g., Frias-Lopez et al., 2008; Stewart et al., 2010; Damon et al., 2012; Raychoudhury et al., 2013). However, the extraction and sequencing of mRNA remains challenging for low biomass environments such as the deep marine subsurface. Only one study so far (Orsi et al., 2013a) has used this approach to report gene expression from anoxic Peru Margin sediments up to 159 mbsf (Site 1229). Here we analyzed sediments from the same Peru Margin site as well as aerobic sediments from Canterbury Basin (Site U1352) in order to expand on those first data on subsurface gene expression for the whole community, and to evaluate whether transcript expression is distinct at comparable depths in these two very different habitats. RNA extraction and metatranscriptome library construction and sequencing of a negative control sample was used to account for reagent and laboratory contamination since contamination can be a major problem for low biomass samples. Biases related to extraction and reverse transcription efficiency are well documented (Alberti et al., 2014; Tsementzi et al., 2014), so interpretation of relative abundances of transcripts should be treated cautiously. The poly-A-focused metatranscriptome library preparation protocol was largely unsuccessful for our samples. Although cDNA was obtained after reverse transcription, analysis of results revealed that the libraries were dominated by concatemers of poly-T sequences. It is likely that extremely low biomass samples can result in production of such artificial sequences (NuGEN technical staff, personal communication). The only sample for which this approach was successful was a Canterbury Basin sample from 345 mbsf (**Supplementary Table S1**). All library preparations were thus repeated using NuGEN Ovation RNA-Seq chemistry, which captures transcripts from all domains of life. For Canterbury Basin analyses, there was not sufficient remaining core material from 3, 30, and 402 mbsf to repeat extractions, so we used samples from 12 and 345 mbsf for further analysis. The sequencing statistics for the successful libraries are presented in **Supplementary Table S2**.

Heterogeneity in metatranscriptome library content was apparent in sediment samples from both sites and between depths (**Supplementary Table S3**). The majority of transcripts were detected in only one of the two replicates, likely reflecting the heterogeneous nature of marine sediments even within individual samples. For this reason the reads from both replicates were combined and co-assembled for analysis. The relative transcript abundances from these combined datasets are discussed below. Transcripts assigned to hypothetical or uncharacterized proteins were excluded from downstream analysis. The number of transcripts is low but we believe that this reflects the low diversity of the subsurface. It is noticeable that the retrieved transcripts decrease with depth (e.g., the CB 12 m b and CB 345 m a samples have similar numbers of raw reads as well as reads that survived quality control, but the assembled transcripts were 3x more in the shallow sample).

Not surprisingly, transcripts encoding housekeeping genes (polymerases, ribosomal proteins, t-RNA synthetases, transcriptional regulators, translation initiation/elongation factors) were abundant in all samples. In all samples, the transcripts assigned to bacteria dominated. The number of transcripts assigned to Archaea decreased with depth. For Peru Margin, 8.4% of archaeal transcripts were detected in the 6 mbsf depth sample and only 1.5% at 95 mbsf. Similarly for Canterbury Basin, 5.6 and 0.9% of archaeal transcripts were present at 12 and 345 mbsf, respectively. The number of fungal transcripts retrieved was 49 from the Peru Margin 6 mbsf sample and 52 from the 95 mbsf sample, representing 0.9 and 2.2%, respectively. A similar trend was observed for Canterbury Basin with 48 fungal transcripts at 12 mbsf and 60 at 345 mbsf, representing 1.0 and 4.6% of transcripts, respectively.

### Central Metabolism

**FIGURE 1:**
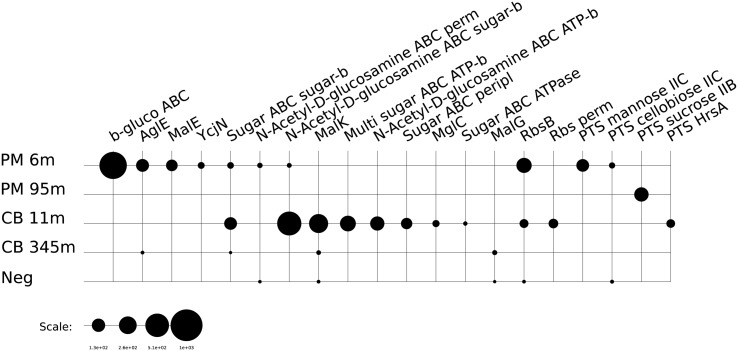
**Relative expression (presented as RPKM values) of genes associated with amino acid/peptide transporters:** BCA ABC trans, branched-chain amino acid ABC transporter, amino acid-binding protein; BCA aminotrans, branched-chain amino acid aminotransferase; DppB, dipeptide transport system permease protein DppB; LivF, branched-chain amino acid transport ATP-binding protein LivF; LivH, high-affinity branched-chain amino acid transport system permease protein LivH; LivG, branched-chain amino acid transport ATP-binding protein LivG; OppA, oligopeptide ABC transporter, periplasmic oligopeptide-binding protein OppA; OppB, oligopeptide transport system permease protein OppB; OppF, oligopeptide transport ATP-binding protein OppF; LivM, branched-chain amino acid transport system permease protein LivM; OppD, oligopeptide transport ATP-binding protein OppD; OppC, oligopeptide transport system permease protein OppC; Oligo ABC trans, oligopeptide ABC transporter, periplasmic oligopeptide-binding protein; ACT peripl, ABC-type amino acid transport/signal transduction systems, periplasmic component/domain; ACT perm, ABC-type amino acid transport system, permease component; DPB ABC perm, ABC-type dipeptide/oligopeptide/nickel transport system, permease component; DPB ABC sub-bind, ABC-type polar amino acid transport system protein, substrate-binding protein; AC ABC trans, amino acid ABC transporter, periplasmic amino acid-binding protein; AC perm, amino acid permease; AC ABC trans ATP, amino acids ABC transporter, ATP-binding protein; AC ABC trans perm, amino acids ABC transporter, permease protein; BCA perm, branched-chain amino acid transport system carrier protein; Cystine trans, cystine ABC transporter, permease protein; DppC, dipeptide transport system permease protein DppC; GltI, glutamate aspartate periplasmic binding protein precursor GltI; PON ABC trans, peptide/opine/nickel uptake family ABC transporter, periplasmic substrate-binding protein; Urea ABC, urea ABC transporter, urea binding protein.

The use of different organic substrates as possible energy sources for subsurface microbial life was examined. The majority of transcripts we recovered that are associated with enzymes involved in carbohydrate, amino acid, and lipid metabolism can participate both in the catabolism of organic material or anabolism/internal recycling of cellular building blocks. We considered the expression of transporters as a proxy for the active catabolic pathways of microbial communities in our samples. Various transcripts indicating an active transport of amino acids and peptides were detected at both depths in all samples examined from Canterbury Basin and Peru Margin. These included transcripts associated with the ABC-type amino acid transport system, branched-chain amino acid transport, *Dpp* dipeptide transport system and *Opp* oligopeptide ABC transport system. The overall relative expression of amino acid and peptide transporters appeared higher at the Canterbury site compared to Peru Margin. While expression of these transporters seems to be decreasing with depth at both sites (**Figure 1** and **Supplementary Table S3**), amino acid metabolism may play an important role as a source of carbon and nitrogen for subsurface microbial communities (Lloyd et al., 2013). The taxonomic assignment of these reads suggests that Archaea may play a role in the utilization of amino acids in the subsurface although to a lesser degree compared to Bacteria.

**FIGURE 2:**
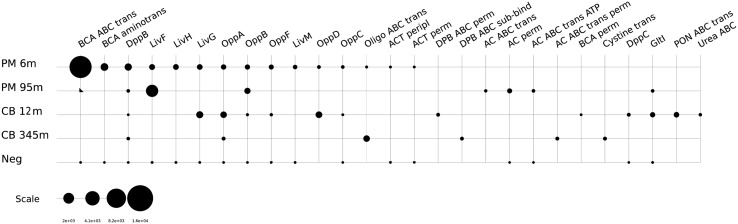
**Relative expression (presented as RPKM values) of genes associated with sugar transporters:** b-gluco ABC, beta-glucoside ABC transport system, sugar-binding protein; AglE, alpha-glucosides-binding periplasmic protein AglE precursor; MalE, maltose/maltodextrin ABC transporter, substrate binding periplasmic protein MalE; YcjN, ABC-type sugar transport system, periplasmic binding protein YcjN; Sugar ABC sugar-b, sugar ABC transporter sugar-binding protein; *N*-acetyl-D-glucosamine ABC perm, *N*-acetyl-D-glucosamine ABC transport system, permease protein 2; *N*-acetyl-D-glucosamine ABC sugar-b, *N*-acetyl-D-glucosamine ABC transport system, sugar-binding protein; MalK, maltose/maltodextrin transport ATP-binding protein MalK (EC 3.6.3.19); Multi sugar ABC ATP-b, multiple sugar ABC transporter, ATP-binding protein; *N*-acetyl-D-glucosamine ABC ATP-b, *N*-acetyl-D-glucosamine ABC transport system ATP-binding protein; Sugar ABC peripl, ABC-type sugar transport system, periplasmic component; MglC, galactose/methyl galactoside ABC transport system, permease protein MglC; Sugar ABC ATPase, ABC-type sugar transport systems, ATPase components; MalG, maltose/maltodextrin ABC transporter, permease protein MalG; RbsB, ribose ABC transport system, periplasmic ribose-binding protein RbsB; Rbs perm, ribose ABC transporter (permease); PTS mannose IIC, PTS system, mannose-specific IIC component; PTS cellobiose IIC, PTS system, cellobiose-specific IIC component (EC 2.7.1.69); PTS sucrose IIB, PTS system, sucrose-specific IIB component (EC 2.7.1.69)/PTS system, sucrose-specific IIC component (EC 2.7.1.69)/PTS system, sucrose-specific IIA component (EC 2.7.1.69); PTS HrsA, PTS system HrsA EIIA component/PTS system HrsA EIIB component/PTS system HrsA permease IIC component.

Sugars and other carbohydrates appear to be additional energy sources for microorganisms in the shallow samples at both sites. Transcripts were detected for the ABC-type sugar transport system, alpha- and beta-glucoside transport system, maltose/maltodextrin ABC transporters, the *N*-Acetyl-D-glucosamine ABC transport system, sugar ABC transporters, the PTS system (cellobiose and mannose specific) and ribose transporters in the Peru Margin 6 mbsf and the Canterbury Basin 12 mbsf samples (**Figure 2**). The overall expression of sugar transporters was found to be higher at the Peru Margin site. This may be a function of the higher overall organic content of the Peru Margin sediments, which may contain higher concentrations of available carbohydrates in shallow subsurface sediments than at Canterbury Basin. Consistent with this idea, transporters of organic acids were also detected in the Peru Margin samples from both depths. High expression of various TRAP dicarboxylate transporters was detected in the Peru Margin 6 mbsf sample. The 95 mbsf showed expression of benzoate MFS transporters (**Supplementary Table S3**).

### Respiration

Transcripts associated with cytochromes (electron transport activities) were not detected in our samples from Peru Margin. In contrast, transcripts for both oxygen and nitrite binding cytochromes were recovered in the shallow sample (12 msbf) from Canterbury Basin in roughly equal relative abundances (**Supplementary Table S3**). Transcripts for the oxygen-utilizing c-type cytochromes were not present in the deep sample (345 mbsf), while the relative abundance of transcripts for the cytochrome cd1 nitrite reductase increased more than 10-fold relative to the 12 mbsf sample. This indicates that respiration through denitrification is an important process at this depth at Canterbury Basin.

### Motility and Cell–Cell Interactions

While motility of cells in subsurface sediments has been proposed (Parkes et al., 2000), flagellar motility in the deep subsurface may be inhibited due to limitations imposed by extremely low expected metabolic rates that would not be expected to provide sufficient energy (Hoehler and Jørgensen, 2013). Consistent with a previous analysis of gene expression in other samples from Peru Margin sediments (Orsi et al., 2013a), we detected expression of flagellar proteins, including the hook-basal body complex (FliE), flagellar biosynthesis proteins (FliC), motor proteins, flagellin, and an RNA polymerase sigma factor for the flagellar operon in the samples we examined (**Supplementary Figure S1**). The highest expression of flagellar-associated protein genes was noted in the sediments from Peru Margin near the sediment surface (6 mbsf depth). Orsi et al. (2013a) also observed a decline in expression of flagellar-associated motility genes between 5 and 91 mbsf, but an increase to near-surface levels again at 159 mbsf. The porosity of our Peru Margin core samples was approximately 45% at both 6 and 95 mbsf (Shipboard Scientific Party, 2003). In our samples from Canterbury Basin, expression of flagellar-associated genes was higher in the sample from 345 mbsf vs. 12 mbsf. The porosity at these two depths was roughly equivalent, approximately 45% (Fulthorpe et al., 2011). We detected expression of genes associated with twitching motility proteins PilJ and PilT in the Peru Margin 6 mbsf and Canterbury Basin 12 mbsf sediments.

We detected expression of genes associated with pili formation, including type IV pilin in sediment samples from Peru Margin (6 mbsf) and Canterbury Basin (12 and 345 mbsf; **Supplementary Figure S1**). Pili are hair-like appendages found on the surfaces of many bacteria that can not only be the attachment site of bacteriophages, but can be involved in bacterial adherence, conjugation, and movement (Shi and Sun, 2002). Greater expression of genes for these proteins was observed in our 6 mbsf vs. 95 mbsf sediments from Peru Margin, and expression was roughly similar in shallow and deep Canterbury Basin sediment samples. Adhesin proteins – which are found on bacterial cell surfaces or appendages, facilitate attachment to other cells or surfaces and are, therefore, another sign of interactions between cells and their environment – were detected in samples from both Peru Margin depths. Collectively, these data all suggest that the heterogeneous nature of subsurface sediments may allow for cellular motility when adequate porosity exists and a sufficient supply of electron donors and acceptors permits metabolic rates that can support motility.

Expression of genes associated with toxin and antitoxin production, and antibiotic production and resistance in sediment samples is an indication of a microbial community that is interacting and actively competing for resources. Toxin and antimicrobial/antibiotic transcripts were detected in libraries from both depths studied at each site (**Supplementary Figure S2**). These included transcripts for beta-lactamases in the Peru Margin 6 mbsf and Canterbury Basin 345 mbsf sample at similar relative abundances (0.19 and 0.11%, respectively). Such enzymes are produced by bacteria to defend their cell wall peptidoglycan-synthesizing machinery against the toxic effects of penicillin derivatives (Goffin and Ghuysen, 1998) and other beta-lactam antibiotics, such as cephalosporins and carbapenems. Transcripts associated with terpenoid backbone biosynthesis were detected in the same samples. Terpenoid secondary metabolites, including naphterpin, terpentecin, napyradiomycin, BE-40644, and furaquinocin, are known to be produced by Actinomycetes (Takagi et al., 2005). We also note the presence of transcripts in all samples aside from Canterbury 345 mbsf associated with numerous described antimicrobials/antibiotics, most of them being retrieved from Canterbury Basin at 12 mbsf at different relative abundances, i.e., streptomycin (2.5%), YoeB toxin (0.2%), vibriolysin (0.02%), and tetracycline (0.01%), followed by 95 mbsf Peru Margin with bacitracin (0.3%) and 6 mbsf Peru Margin with chloramphenicol (0.1%). However, transcripts involved in emericellamide and penicillin biosynthetic processes were also detected in the poly-A enriched metatranscriptome library from Canterbury Basin 345 mbsf (see “Fungal Diversity and Activity Based on iTAG and Poly-A-Focused Metatranscriptome Analyses of Canterbury Basin and Peru Margin Samples” of the Discussion). Multidrug eﬄux and extrusion (Na^+^/drug antiporter) proteins were some of the most highly expressed genes associated with antimicrobial activities in the Peru Margin 6 and 95 mbsf samples. High expression of a permease gene associated with the drug/metabolite transporter (DMT) superfamily was noted in the Canterbury Basin 12 mbsf sample. Transporters, including quaternary ammonium compound-resistance protein *SugE*, involved in multidrug resistance (Chung and Saier, 2002), were detected in all samples, including the Canterbury 345 mbsf sample where these were relatively highly expressed. Transcripts of inositol-1-phosphate synthase were also highly expressed in the Peru Margin 6 mbsf sample. While this gene can be associated with inositol phosphate metabolism, it can also participate in streptomycin biosynthesis by members of *Streptomyces* (Pittner et al., 1979). All beta-lactam antibiotics target proteins associated with cell division and cell wall integrity (Koch, 2000). We note that transcripts for multimodular transpeptidase–transglycosylase, involved in production of high molecular weight penicillin-binding proteins used in growth and division of the bacterial cell envelope were highly expressed in the 95 mbsf Peru Margin sample, suggesting that bacterial cell division at the depth where this sample was collected may be active enough for targeting by certain antibiotic compounds. Deep subseafloor secondary metabolisms analyzed here highlight complex cell–cell interactions between microbial communities from all three domains of life, especially between bacteria and fungi. Peru Margin and Canterbury basin sediment samples from 6 to 345 mbsf may be allegorized as chemical battlefields and may open an era of metabolomics to better understand interactions between microbial communities using metabolite profiling as a complementary approach.

### Heavy Metals

Concentrations of particular heavy metals in the marine subsurface are likely to vary considerably with depth due to variations in the depositional environment when the sediments were originally deposited on the seafloor as well as subsequent intrusions of metal-containing fluids. Concentrations and compositions of heavy metals such as cadmium, mercury, lead, arsenic, cobalt, and copper are known to shape microbial community composition because of their toxicity (e.g., Oliveira and Pampulha, 2006; Ravikumar et al., 2007). These metals can bind to vital cellular structural proteins, enzymes, and nucleic acids, and can thus interfere with their function (e.g., Srivastava and Goyal, 2010; Olaniran et al., 2013). We detected expression of general ion transport-related genes included those affiliated with siderophore biosynthesis, as well as general magnesium and iron transporters and chelatases not necessarily associated with detoxification activities (**Supplementary Figure S3**). In addition, we note the presence of arsenic resistance transcripts in libraries from both depths of Canterbury Basin. Transcripts for cobalt–zinc–cadmium resistance proteins were detected in all samples at relatively high levels. While these metals can be involved in general metabolic functions, our detection of eﬄux system proteins for the same metals suggests cells in these samples may face challenges with balancing intra- and extra-cellular concentrations of these metals. Except for arsenic resistance for which no transcripts were obtained, the same trend was observed in the poly-A enriched metatranscriptome library from Canterbury Basin 345 mbsf (see below). Microorganisms in anoxic sediments are known to be capable of respiring through the coupled reduction of iron or sulfur and toxic metals such as arsenic (Reyes et al., 2008). These organisms commonly use chaperone proteins to counter heavy metal-induced protein aggregation (Tamas et al., 2014). Expression of genes associated to movement of arsenic ions across the cell membrane (e.g., arsenical pump-driving ATPases in the Canterbury Basin 345 mbsf sample) has been observed. Arsenic (As) bioabsorption and/or volatilization by terrestrial fungi in myco bioremediation processes have highlighted the ability of some fungi to cope with As and to accumulate low concentrations in their biomass (Cernansky et al., 2007; Adeyemi, 2009). Inversely, when As concentrations are far too high, for example at seafloor hydrothermal fields, fungal organic matter can serve as a geochemical trap for As, which seems to react with S and mineralize the fungal hyphae in a process leading to a dead-end biosequestration fate (Dekov et al., 2013). Here, evidence for arsenic resistance transcripts may support the ability of microorganisms to cope with non-lethal *in situ* arsenic concentrations.

### Fungal Diversity and Activity Based on iTAG and Poly-A-Focused Metatranscriptome Analyses of Canterbury Basin and Peru Margin Samples

#### Peru Margin vs. Canterbury Basin iTAG Analyses

**FIGURE 3:**
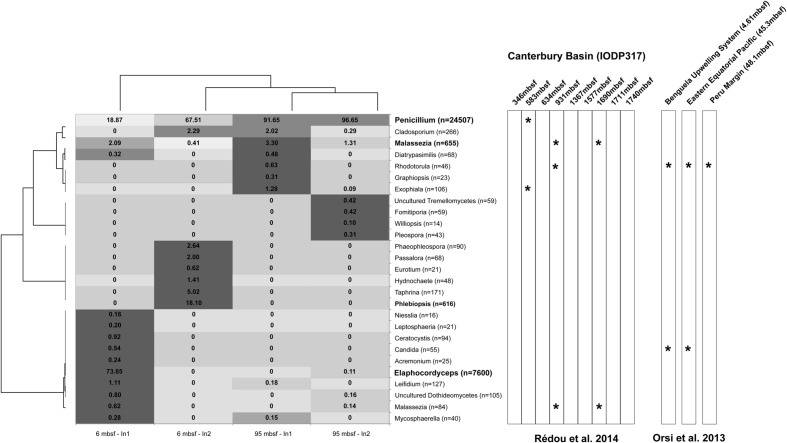
**Heat map highlighting the difference of fungal OTUs between 6 and 95 mbsf Peru Margin sediment samples using iTAG**. Numbers presented are the percentage of reads for a particular OTU in each sample. Colors correspond to the relative abundance of individual OTUs across samples. Black corresponds to taxon present at ≥80%, dark gray ≥60%, light gray ≥40%, and white <40%. Dendograms are based on normalized read abundances for samples (top) and taxonomic groups (left). A complementary heat map highlights the overlapping genera (^∗^) between this study and those of Orsi et al. (2013a) and Rédou et al. (2014).

Fungal iTAG sequences obtained from Peru Margin samples indicate low fungal diversity in both the 6 and 95 mbsf samples. From exterior control replicates we obtained 52 and 14,447 quality fungal sequences for the 6 mbsf sample, and 23,275 and 42,231 sequences for the 95 mbsf sample. From core interior replicate samples we obtained 10,286 and 3,438 quality sequences from the 6 mbsf samples, and 14,110 and 7,387 sequences from the 95 mbsf samples. For the 6 and 95 mbsf core interior samples we obtained a total of 60 and 49 fungal OTUs at 97% sequence similarity. After removing OTUs with fewer than 10 reads, we retained 20 and 16 OTUs for each sample, respectively. These results are consistent with Rédou et al. (2014) who removed iTAGs that represented singletons prior to clustering, and then obtained nine OTUs from their Canterbury Basin sample from 345 mbsf. After removing OTUs containing less than 10 reads, Rédou et al. (2014) retained seven OTUs for downstream analyses. This suggests overall fungal diversity is roughly consistent (same order of magnitude) between the two sites at the depths examined, with slightly more diversity in the Peru Margin samples. We discuss the results for replicate samples as one pooled sample per depth because even at the family level OTU representation was highly dissimilar, likely due in part to the heterogeneous nature of sediment subsamples. Additionally, discussing results for replicates as one pool allows a more accurate comparison between depths because of uneven sequence recovery. This was particularly true for one of our exterior control replicates from 6 mbsf (for which we do not have an explanation for the low sequence recovery). There were only three groups for which sequences were detected exclusively in the core exterior samples vs. the core interior (the families Clavicipitaceae, Pyxidiophoraceae, and an unclassified Tremellomycete) at 6 mbsf. These were low abundance OTUs, and given the variable sequence recovery of replicate samples and evident sediment heterogeneity, we interpret this result with caution and do not feel this is unequivocal proof these taxa are contaminants. The exterior control data sets were deemed not to be informative for serving as a contamination control, and were not utilized further. They are available from the authors upon request. For the 95 mbsf sample OTUs from the families Clavicipitaceae and Pyxidiophoraceae were also detected exclusively in the exterior samples, along with OTUs affiliated with *Sporothrix* sp. and *Ceriporia purpurea*, and all were at low abundance. Detection of overlap (or lack thereof) between interior and exterior OTUs is inclusive as a means of detecting contamination, since one would expect to find the same OTUs present in the absence of contamination, and if sequencing depth is not sufficient, absence is difficult to interpret. Other means of controlling for sampling and laboratory contamination are clearly required, such as sequencing drilling fluids. For our analyses of iTAG libraries, OTUs encompassing less than 10 reads were discarded for discussion. The dominant fungal OTU (70% of all reads) in samples from both Peru Margin depths analyzed here affiliated with the filamentous fungal genus *Penicillium* of the Trichocomaceae family (∼70–95% of OTUs in both 95 mbsf replicates and one of the 6 mbsf replicates) with higher abundance in the deeper sample (**Figure 3**). The filamentous fungal genus *Elaphocordyceps* of the Ophiocordycipitaceae family accounts for 20% of all Peru Margin reads and was retrieved almost exclusively from one replicate sample from 6 mbsf (∼70% of OTUs of one 6 mbsf replicate, **Figure 3**). OTUs related to the yeast-like genus *Malassezia* and to the filamentous fungal genus *Cladosporium* were detected in both 6 and 95 mbsf core interiors, with higher abundance in the deeper sample (**Figure 3**).

Fungal iTAG sequencing from Canterbury basin sediment samples was previously reported for sediments from 345 to 1711 mbsf (Rédou et al., 2014). That study also demonstrates a low fungal diversity within the samples analyzed. Indeed, only 22 different genera were obtained. Four genera detected by Rédou et al. (2014) were retrieved in our Peru Margin iTAG analyses (*Penicillium*, *Malassezia, Rhodotorula*, and *Exophiala*) as shown in **Figure 3**. This suggests that the fungal taxonomic composition of samples at both sites is not the same, although we interpret these differences with caution since the depths analyzed at the two sites were different and since different amplification strategies were used (fungal targeting primers here vs. eukaryotic targeting primers in previous studies). Such overlapping genera have been previously detected in different marine environments, including deep subseafloor habitats. Indeed, *Penicillium* is a ubiquitous genus that has been detected in marine subsurface samples from 582 mbsf at Canterbury Basin (Rédou et al., 2014) and isolates of this genus were also obtained from the same core (Rédou et al., 2015). *Malassezia* is an enigmatic well-known genus from terrestrial environments (Amend, 2014) and signatures of this genus have recently been repeatedly retrieved from deep-sea samples using DNA-based analyses (Bass et al., 2007; Lai et al., 2007; López-García et al., 2007; Le Calvez et al., 2009; Jebaraj et al., 2010; Nagahama et al., 2011; Singh et al., 2011; Stock et al., 2012; Xu et al., 2014; Sohlberg et al., 2015) as well as rRNA-based analyses of Peru Margin samples from 1.75 and 35 mbsf (Edgcomb et al., 2011) and Canterbury Basin samples from 931 to 1689 mbsf (Rédou et al., 2014). However, to date no isolates of *Malassezia* have been obtained using marine environment samples. *Rhodotorula* appears as a widely distributed genus and is common in deep-sea environments (Nagahama et al., 2001; Gadanho and Sampaio, 2005; Bass et al., 2007; Burgaud et al., 2010; Singh et al., 2011) including deep subseafloor sediments where *Rhodotorula* was retrieved using iTAG sequencing from the three deepest samples analyzed by Orsi et al. (2013a), i.e., Benguela Upwelling System, Eastern Equatorial Pacific and Peru Margin (**Figure 3**) and was the most abundant culturable yeast from Canterbury Basin sediment samples (Rédou et al., 2015). Recent analyses have clearly demonstrated the ability of yeasts affiliated to the genus *Rhodotorula* to support elevated hydrostatic pressure conditions (Burgaud et al., 2015). Signatures of *Exophiala* have been detected in marine subsurface samples at 583 mbsf at Canterbury Basin (Rédou et al., 2014) and this genus was also isolated from the same core (Rédou et al., 2015). Isolates of *Exophiala* were also recently obtained from deep-sea hydrothermal vent samples (Burgaud et al., 2009).

The predominant fungal OTUs identified from Canterbury 345 mbsf sample were affiliated to the yeast phylotypes *Cryptococcus curvatus, Trichosporon* sp., *Meyerozyma guilliermondii, Cyberlindnera jadinii* and *Filobasidium globisporum*. This is consistent with previous studies addressing micro-eukaryotic diversity that suggest marine dikarya are dominated by yeast forms in water columns (Bass et al., 2007) and superficial sediment samples (Richards et al., 2015). The *Cryptococcus*, *Cyberlindnera, Trichosporon*, and *Filobasidium* yeast genera were also identified in subsurface sediments in RNA-based studies of Peru Margin sediments (Edgcomb et al., 2011; Orsi et al., 2013a). Among the filamentous fungi, OTUs affiliated to the genera *Fusarium* and *Trichoderma* were identified from the Canterbury 345 mbsf sample. Interestingly, molecular signatures of *Fusarium* were also detected in deep-sea sediments of the Pacific Ocean (Xu et al., 2014) and in Arctic marine sediments (Zhang et al., 2015). Recovery of cultured isolates affiliated with the genus *Fusarium* and the species *Meyerozyma guilliermondii* from the Canterbury basin (Rédou et al., 2015) and isolates of *Trichosporon* sp. from St. Helena Bay in South Africa (Mouton et al., 2012) support the notion that at least a portion of buried fungal communities detected by molecular methods is able to persist in deep-subseafloor sediment.

#### Eukaryote-Focused Metatranscriptome Analysis of a Canterbury Basin Sample from 345 mbsf

**FIGURE 4:**
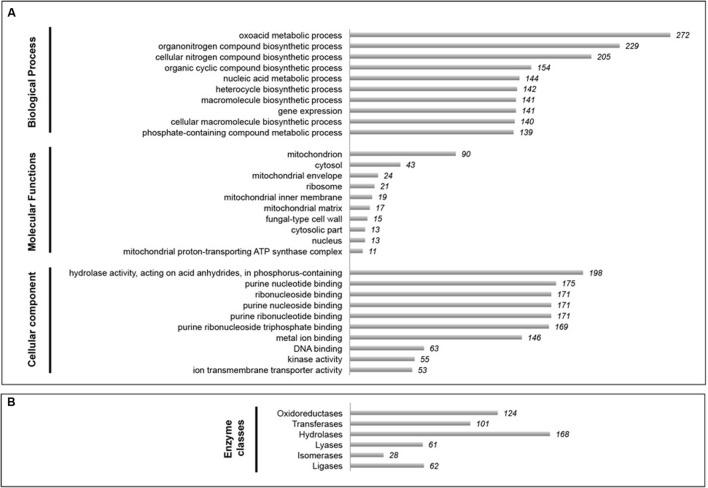
**Snapshot of fungal metabolic activities in 345.50 mbsf Canterbury Basin sediment sample. (A)** Top 10 Gene Ontology (GO) terms based on GO analysis of biological processes, molecular functions, and cellular components (GO level 4). **(B)** Distribution of enzyme classes as a proxy for fungal catalytic activity.

To examine the functional repertoire of the Fungi, a poly-A targeted metatranscriptomic approach was employed. Fourteen poly-A enriched metatranscriptome libraries were constructed and sequenced in total (**Supplementary Table S1**). Out of these, only one (Canterbury Basin 345 mbsf sample) produced high quality sequences and will be discussed below. For this sample the *de novo* assembly using 5,250,985 quality-trimmed reads generated 11,389 contigs with an average length of 1,705 bp. Nine hundred and thirty sequences were unambiguously assigned to fungal taxa, indicating that fungi appeared metabolically active in this deep subseafloor sample. As fungal databases improve, a larger fraction of our “unassigned” sequences may be possible to assign to fungi. Nonetheless, a wide diversity of fungal transcripts involved in different functions was represented in the dataset based on Gene Ontology term analysis (**Supplementary Tables S4**–**S6**). Preliminary analyses indicated that GO term levels 2 and 3 were not informative enough. Final analysis was therefore conducted at the GO term level 4. The three descriptive ontologies “cellular components,” “molecular function,” and “biological process” were analyzed (**Figure 4A**). Gene expression from fungal communities was mainly assigned to metabolic and biosynthetic processes, e.g., amino acid, fatty acid, peptide, and ATP biosynthetic processes, but also responses to stress, and cell and membrane functions, e.g., ergosterol biosynthetic process, cell wall and septum formation. These results provide further support for the notion of fungal activity in the deep subseafloor at record depths, extending the previously known depth limits of eukaryotic functions from 159 to 345 mbsf.

Several DNA-based analyses have revealed the presence of fungal communities in the deep subseafloor (Edgcomb et al., 2011; Orsi et al., 2013a,b; Ciobanu et al., 2014), but only two studies provide evidence that part of these communities is active. Edgcomb et al. (2011) and Orsi et al. (2013b) used an rRNA and mRNA-based analysis, respectively, to report activity and functions in the deep subseafloor. In a study of Peru Margin subseafloor sediments Orsi et al. (2013b) reported fungal cell-division transcripts, indicating actively dividing cells, but also fungal transcripts involved in carbohydrate, amino acid, and lipid metabolism, suggesting that fungi have a role in organic carbon turnover at this site (Orsi et al., 2013b). In our eukaryote-focused metatranscriptome from 345 mbsf at Canterbury Basin, cell-division transcripts (cytokinesis) were also retrieved indicating actively dividing fungal cells at 345 mbsf., Fungal transcripts involved in mycelium development, filamentous growth, fungal cell membrane (ergosterol biosynthesis), and hyphal growth were detected, also providing concrete evidence for active fungal growth in deeply buried sediment. Spore germination and conidium formation transcripts were detected in this library, indicative of fungal sporulation in this deep biosphere. This non-exhaustive list of fungal transcripts clearly indicates that physical and geochemical constraints at 345 mbsf are not a bottleneck for fungal life.

Transcripts involved in fungal ergosterol biosynthesis were detected. Ergosterol is an important component of fungal membranes but it is also known to have a protective role for fungal cell membranes (Fernandes et al., 2004). It was shown that *S. cerevisiae* is able to modify its membrane composition in order to tolerate high hydrostatic pressure conditions by increasing the proportion of unsaturated fatty acids and ergosterol (Simonato et al., 2006). This adaptive strategy increases membrane fluidity to counteract the effect of higher hydrostatic pressure, and thus, helps maintain the functionality of cell membranes in deep ecosystems. The occurrence of ergosterol biosynthesis transcripts suggests this may be one mechanism by which deep subseafloor fungi are able to cope with elevated hydrostatic and lithostatic pressures in the deep biosphere.

Several types of fungal transcripts were detected that are involved in stress tolerance, i.e., starvation, heat, hypoxia, oxidative stress, and DNA damage. These included trehalose-6-phosphate synthase, heat-shock proteins, chaperonins, catalase, glucose-6-phosphate isomerase, glutathione peroxidase, thioredoxin, uracil-dna glycosylase, methyltransferase, histone demethylase, adenylosuccinate synthetase. Such transcripts indicate stress responses occur and represent mechanisms by which fungi are able to cope with subsurface conditions. DNA degradation could be a major obstacle to long-term survival in a dormant state. As previously suggested by Jørgensen (2011) and Orsi et al. (2013b), the occurrence of DNA repair transcripts also in the Canterbury metatranscriptome, supports the idea that dormancy may not be a successful survival strategy in the deep biosphere, both for prokaryotes and fungi. Indeed, maintaining adequate levels of intracellular DNA repair enzymes may represent an adaptive mechanism to handle the slow degradation of DNA over geological timescales.

Significant catalytic activities were also reported within different classes of enzymes such as hydrolases, oxidoreductases, transferases, ligases, lyases, and isomerases (**Figure 4B**). We hypothesize that such enzymes are predominantly exoenzymes secreted outside the cell to break down complex macromolecules into smaller units for subsequent absorption. Given that overall numbers of fungal transcripts were higher at increasing depths at both Peru Margin and Canterbury Basin (based on the whole community metatranscriptomes described above) this complex enzymatic fingerprint revealed in our 345 mbsf sample may reflect the increased abilities of fungi to degrade refractory organic matter in the deep, possibly more specialized, fungal biosphere. These results are consistent with previous metatranscriptomic work from Peru Margin sediments (Orsi et al., 2013b) and suggest that active fungal communities play a role in biogeochemical cycling and with organic carbon turnover in subseafloor sediments by virtue of their ability to efficiently degrade a wide range of biopolymers.

Nitrate reduction has been reported to occur in the deep subseafloor with *Alphaproteobacteria* and *Betaproteobacteria* as predominant actors (Orsi et al., 2013b). Those authors argued that the resulting nitrite was probably reduced by fungi, *Gammaproteobacteria*, and *Firmicutes*. Indeed, transcripts involved in nitrite reduction were reported in our poly-A enriched Canterbury Basin metatranscriptome dataset, supporting the idea of syntrophic interactions in the deep subseafloor. These results are also supported by previous studies that report the potentially active role of fungi in denitrification within marine sediments (Jebaraj et al., 2010; Mouton et al., 2012).

Fungal expression of genes for biosynthesis of the secondary metabolites asperthecin, penicillin, and emericellamide was detected. Fungal expression of siderophores was also detected. While siderophores are known to play a role in transport of metals, they may also play a role in antioxidant and antibiotic activities (Sebat et al., 2001). Microbial expression of genes associated with defense mechanisms and secondary metabolite biosynthetic processes, such as, non-ribosomal peptide biosynthetic processes suggests that fungi are able to interact with, and compete with, prokaryotic communities occurring in the deep subseafloor. A previous study provided direct evidence that fungal isolates from deep subseafloor sediments harbor genes involved in secondary metabolite biosynthetic pathways (Rédou et al., 2015). That study and ours indicate further investigation is warranted to search for potentially novel and useful secondary metabolites produced by deep subseafloor microorganisms, including Fungi. We hypothesize that the deep biosphere represents an exciting ecosystem for searching for novel biomolecules that may help us tackle the innovation crisis related to the development of new molecular entities in the pharmaceutical industry (Kling, 2014).

### Microscopical Analysis of Canterbury Basin Samples from 4 and 403 mbsf

**FIGURE 5:**
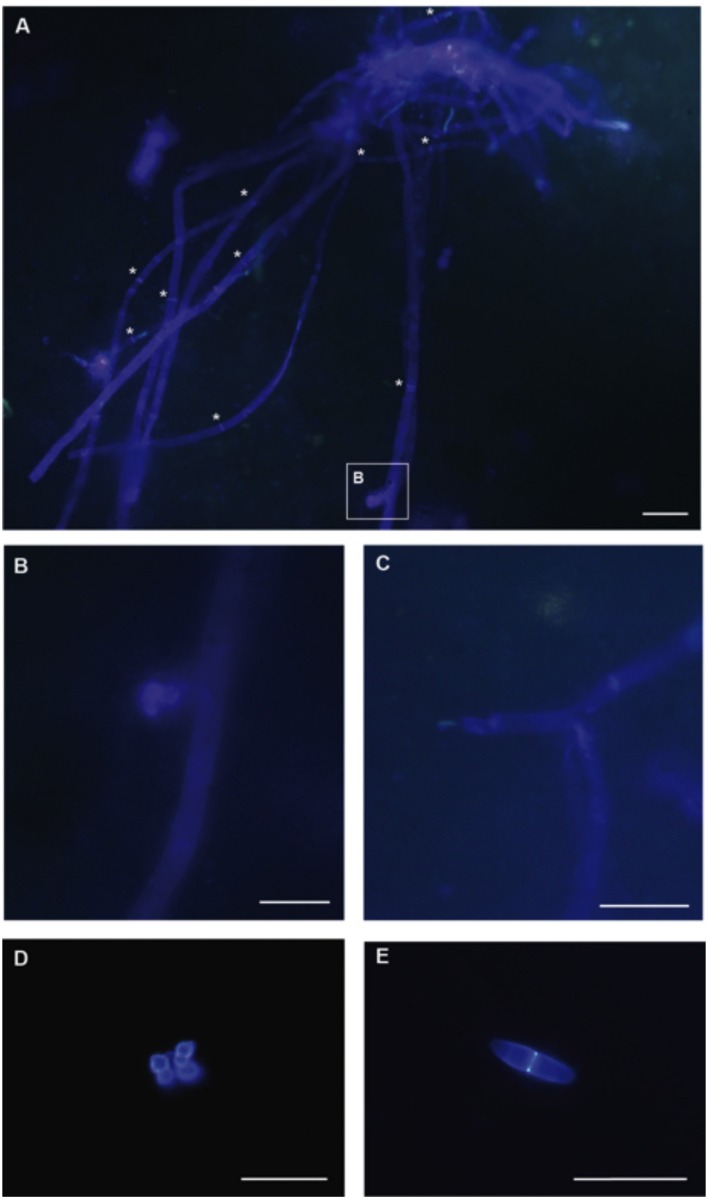
**Microscopical analysis of Calcofluor stained fungal cells using 4 mbsf (A–D) and 4000 mbsf (E) Canterbury Basin sediment samples. (A)** Fungal hyphae compartmentalized by septa, highlighted with white stars (Scale bar, 10 μm). **(B)** Fungal conidiogenesis (Scale bar, 5 μm). **(C)** Fungal filamentous branching (Scale bar, 10 μm). **(D)** Cluster of fungal spores (Scale bar, 10 μm). **(E)** Fungal septate spore (Scale bar, 10 μm).

Fossilized fungi have been documented previously from deep subsurface habitats (e.g., Ivarsson et al., 2013). The aim of our cell visualization efforts was to visualize for the first time, living fungal cells in deep subseafloor sediments. The presence of fungal cells was detected in samples from two depths, 1H3 (4 mbsf) and 48X3 (403 mbsf). Fungal biomass appears to be very low, as few fungal cells were observed, and thus no quantification or estimation of abundance could be established based on this study. However, direct evidence of septate fungal filaments (**Figure 5A**), conidiogenesis (**Figure 5B**), branching (**Figure 5C**), and spores at 4 mbsf (**Figure 5D**) and 403 mbsf (**Figure 5E**) highlight the ability of deep subseafloor fungi to grow as filaments and reproduce (even asexually). Although no specific taxonomic features were observed, the presence of septa clearly indicates those fungi are Dikarya, i.e., ascomycetes or basidiomycetes. Recent studies of deep-sea habitats show that fungal diversity consists of a mixture of basal and higher lineages. Using molecular tools, basal fungal lineages, mostly Chytridiomycota and Cryptomycota, have been detected in extreme environments such as cold seeps (Nagahama et al., 2011; Thaler et al., 2012), deep sediments (Nagano et al., 2010) and hydrothermal vents (Bass et al., 2007; Le Calvez et al., 2009). However, marine fungal diversity in deep-sea habitats, including deep subsurface sediments, appears mostly comprised of the higher fungal phyla Ascomycota and Basidiomycota (Ciobanu et al., 2014; Rédou et al., 2014, 2015). Consistent with previous studies, only fungal cells from these higher fungal phyla have been visualized in this study. All data available, i.e., from culture-based methods, molecular analyses, and cell visualization, strongly support the idea that deep subsurface fungi are almost exclusively affiliated to the Ascomycota and Basidiomycota phyla.

## Conclusion

Whole community metatranscriptome analyses reveal higher relative expression of amino acid and peptide transporters in Canterbury Basin vs. Peru Margin sediments, possibly reflecting the lower organic content at this site and hence, the greater challenge microorganisms face for obtaining needed nutrients. Genes associated with cell motility were more highly expressed in the more organic-rich Peru Margin sediments. High expression of heavy metals transporters and eﬄux systems in our Canterbury Basin samples suggests heavy metals toxicity may be a greater challenge for microorganisms at that site relative to Peru Margin. A poly-A focused metatranscriptome produced for our Canterbury Basin sample from 345 mbsf provides further evidence for active Fungi in the subsurface in the form of transcripts for metabolic and cellular processes, cell and membrane functions, and catalytic activities. Consistent with previous observations, our molecular signatures of fungal diversity based on ribosomal RNA iTAGs suggest communities are not dominated by the same taxa in subsurface sediments that vary in organic content and oceanic province. Our microscopic observations of fungal cells and structures presented here, together with our metatranscriptome data and previous culture-based and molecular studies, indicate deep subseafloor filamentous fungi and spores are active members of deep biosphere communities. Indeed, transcripts involved in mycelium development, filamentous/hyphal growth, and conidium formation confirm our microscopic observations based on Calcofluor white staining. Future global studies of the microbiology of the deep biosphere should incorporate additional investigations of fungal populations and their activities.

## Author Contributions

VE, MP, and GB designed this study and drafted the manuscript. DJB and VR performed the molecular work. MP and VR performed the bioinformatic analyses. All authors contributed to data interpretation. All authors read and approved the final manuscript.

## Conflict of Interest Statement

The authors declare that the research was conducted in the absence of any commercial or financial relationships that could be construed as a potential conflict of interest.

## Footnotes

1 http://www.bioinformatics.babraham.ac.uk/projects/trim_galore/

2 https://www.biolynx.ca/pdf/admin/nu_threedgeuserguide.pdf

3 http://dna.uga.edu

## Supplementary Material

FIGURE S1 **Relative expression (presented as RPKM values) of genes associated with motility and cell–cell interactions**.

FIGURE S2 **Relative expression (presented as RPKM values) of genes associated with toxin and antimicrobial/antibiotic synthesis/resistance**.

FIGURE S3 **Relative expression (presented as RPKM values) of genes associated with heavy metal ion transport and detoxification activities**.

Table S1 **Samples processed for iTAG and metatranscriptome analyses with success noted for different approaches.** nd, not done.

Table S2 **Metrics of the metatranscriptomics libraries**.

Table S3 **RPKM values for replicate metatranscriptome libraries prepared from Peru Margin and Canterbury Basin core samples.** Also shown for each depth/site are RPKM values calculated for data sets produced by combining replicate libraries.

Table S4 **Profile of fungal metabolic activities in our 345.50 mbsf Canterbury Basin metatranscriptome as revealed by the distribution of contigs across the “biological functions” category as detected by Gene Ontology (GO) term analysis at the GO level 4**.

Table S5 **Profile of fungal metabolic activities in our 345.50 mbsf Canterbury Basin metatranscriptome as revealed by the distribution of contigs across the “molecular functions” category as detected by Gene Ontology (GO) term analysis at the GO level 4**.

Table S6 **Profile of fungal metabolic activities in our 345.50 mbsf Canterbury Basin metatranscriptome as revealed by the distribution of contigs across the “cellular component” category as detected by Gene Ontology (GO) term analysis at the GO level 4**.
