# Supplementary figures and images for: Fungal and Prokaryotic Activities in the Marine Subsurface Biosphere at Peru Margin and Canterbury Basin Inferred from RNA-Based Analyses and Microscopy
Source: Front Microbiol. 2016 Jun 9;7:846. doi: 10.3389/fmicb.2016.00846 (PMC4899926; doi:10.3389/fmicb.2016.00846)

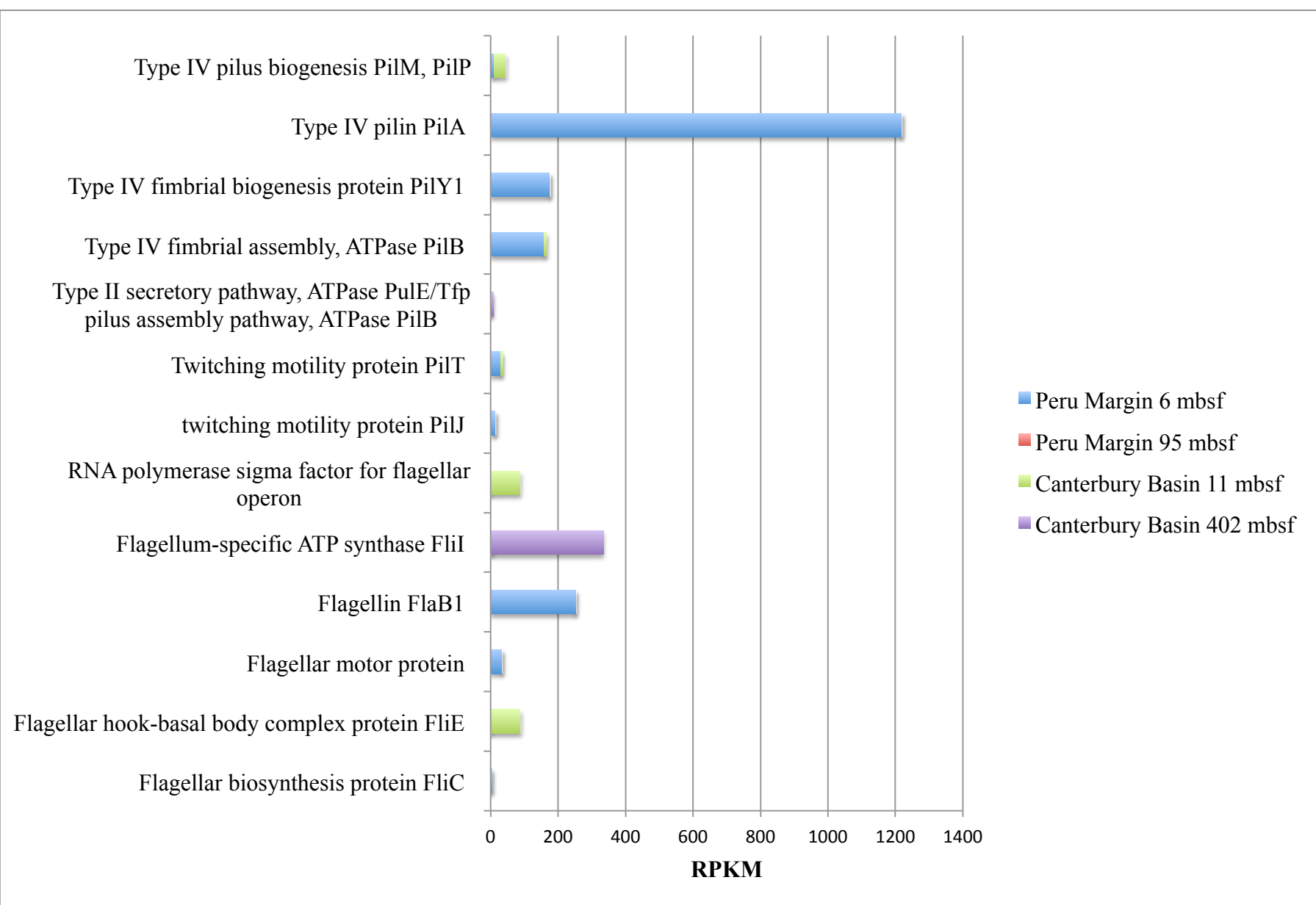

Supplement: FIGURE S1 — Relative expression (presented as RPKM values) of genes associated with motility and cell–cell interactions. [file Image_1.PDF]

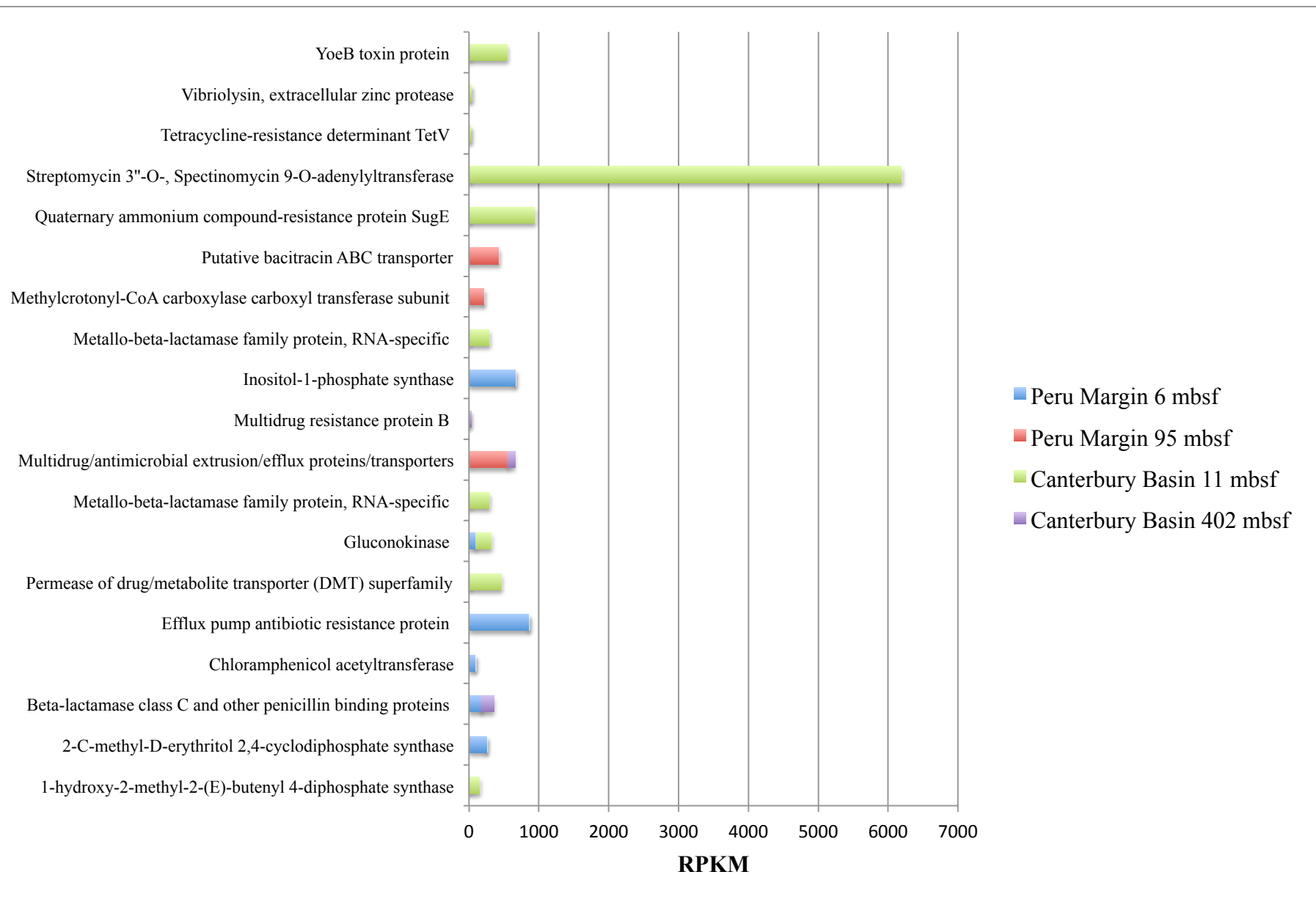

Supplement: FIGURE S2 — Relative expression (presented as RPKM values) of genes associated with toxin and antimicrobial/antibiotic synthesis/resistance. [file Image_2.PDF]

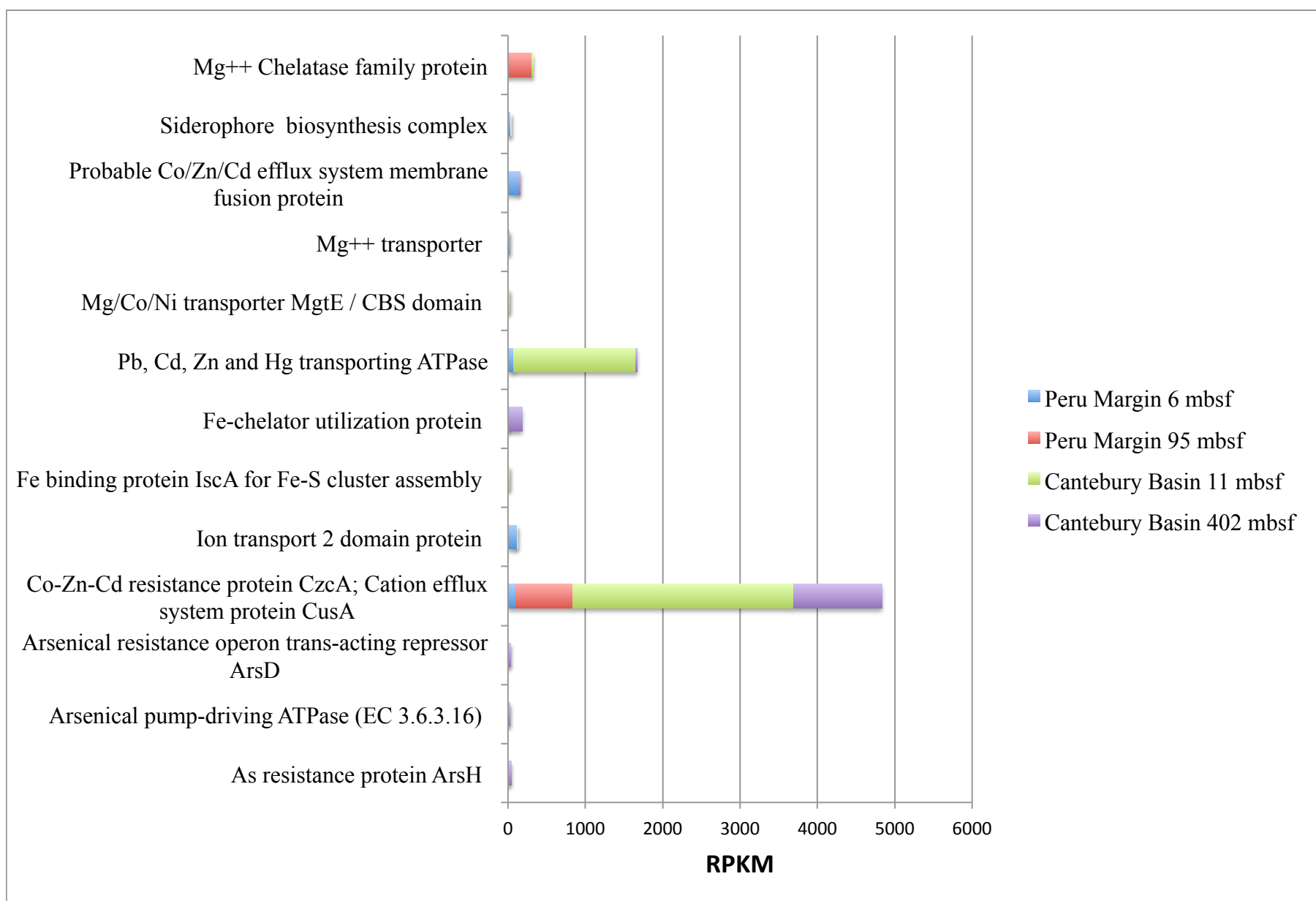

Supplement: FIGURE S3 — Relative expression (presented as RPKM values) of genes associated with heavy metal ion transport and detoxification activities. [file Image_3.PDF]
