# Supplementary material for: Fungal and Prokaryotic Activities in the Marine Subsurface Biosphere at Peru Margin and Canterbury Basin Inferred from RNA-Based Analyses and Microscopy
Source: Front Microbiol. 2016 Jun 9;7:846. doi: 10.3389/fmicb.2016.00846 (PMC4899926; doi:10.3389/fmicb.2016.00846)
Supplement: Table S1 — Samples processed for iTAG and metatranscriptome analyses with success noted for different approaches. nd, not done. [file Table_1.DOCX]

STable 1. Samples processed for iTAG and metatranscriptome analyses with success noted for different approaches. Experiment performed in study by Redou et al. (2014) noted with *

| Sample | Fungal iTAGs | 3’DGE (polyA-enriched) metatranscriptome | NuGEN Ovation RNA-Seq metatranscriptome |
| --- | --- | --- | --- |
| Peru Margin 6 mbsf | Successful (2 replicates) | Failed | Successful (2 replicates) |
| Peru Margin 95 mbsf | Successful (2 replicates) | Failed | Successful (2 replicates) |
| Canterbury Basin 4 mbsf | nd | Failed | nd |
| Canterbury Basin 12 mbsf | nd | Failed | Successful (2 replicates) |
| Canterbury Basin 25 mbsf | nd | Failed | nd |
| Canterbury Basin 345 mbsf | *Successful (2 replicates) | Successful (1 replicate) | Successful (2 replicates) |
| Canterbury Basin 403 mbsf | nd | Failed | nd |
