# Supplementary material for: Fungal and Prokaryotic Activities in the Marine Subsurface Biosphere at Peru Margin and Canterbury Basin Inferred from RNA-Based Analyses and Microscopy
Source: Front Microbiol. 2016 Jun 9;7:846. doi: 10.3389/fmicb.2016.00846 (PMC4899926; doi:10.3389/fmicb.2016.00846)
Supplement: Table S3 — RPKM values for replicate metatranscriptome libraries prepared from Peru Margin and Canterbury Basin core samples. Also shown for each depth/site are RPKM values calculated for data sets produced by combining replicate libraries. [file Table_3.PDF]

Transcript	CB 11mbsfA	CB 11mbsfB	CB 11mbsfCombined	CB 345mbsfA	CB 345mbsfB	CB 345mbsfCombined	PM 6mbsfA	PM 6mbsfB	PM 6mbsfCombined	PM 95mbsfA	PM 95mbsfB	PM 95mbsfCombined	Negative
1-hydroxy-2-methyl-2-(E)-butenyl 4-diphosphate synthase (EC 1.17.7.1)	278.434	0	145.384	0	0	0	0	0	0	0	0	0	0.692421
16S rRNA processing protein RimM	9.29486	0	4.87477	0	0	0	0	0	0	0	0	0	0.559999
2-5A-dependent ribonuclease (EC 3.1.26.-)	0	0	0	0	0	0	0	0	0	1027.21	0	447.838	0
2-keto-3-deoxy-D-arabino-heptulosonate-7-phosphate synthase I beta (EC 2.5.1.54)	0	0	0	0	0	0	0	0	0	257.549	0	132.086	0
2-keto-3-deoxy-D-arabino-heptulosonate-7-phosphate synthase II (EC 2.5.1.54)	0	0	0	16.35539	0	9.37581	0	0	0	0	0	0	0
2-keto-4-pentenoate hydratase/2-oxohepta-3-ene-1,7-dioic acid hydratase (catechol pathway)	0.203281	0	0.106613	0	0	0	0	0	0	0	0	0	0
2-nitropropane dioxygenase (EC 1.13.11.32)	0.197908	0	0.103795	0	0	0	0	0	0	0	0	0	0
2-oxobutyrate oxidase, putative	1.26805	0	0.665039	0	0	0	0	0	0	0	0	0	0
2-oxoglutarate oxidoreductase, alpha subunit (EC 1.2.7.3)	0	0	0	0	0	0	96.3397	175.447	134.4992	0	0	0	0
2-oxoglutarate oxidoreductase, beta subunit (EC 1.2.7.3)	0	0	0	0	0	0	0	5.11414	2.52264	0	0	0	0
2-oxoglutarate oxidoreductase, delta subunit, putative (EC 1.2.7.3)	0	3.21307	1.52795	0	0	0	0	42.8011	0	0	0	0	0
2-polyprenyl-6-methoxyphenol hydroxylase and related FAD-dependent oxidoreductases	354.452	0	208.3505	0	0	0	0	0	0	0	0	0	1.924201
2,3-bisphosphoglycerate-independent phosphoglycerate mutase, archaeal type (EC 5.4.2.1)	0	0	0	0	0	0	0	30.2116	16.9038	0	0	0	0
2,4-dienoyl-CoA reductase (NADPH) (EC 1.3.1.34)	0	0	0	0	0	0	60.7589	0	12.25685	0	0	0	0
23S rRNA (Uracil-5-) -methyltransferase rumB (EC 2.1.1.-)	0.18115	0	0.0950056	0	0	0	0	0	0	0	0	0	0
3-dehydroquinate synthase (EC 4.2.3.4)	3527.66	0	1956.853	0	0	0	0	0	0	0	0	0	0
3-hydroxyacyl-CoA dehydrogenase type II	0	0	0	0	0	0	0	0	0	0	12.4315	6.09159	0
3-hydroxybutyryl-CoA dehydratase (EC 4.2.1.55)	0	11.0331	5.2467	0	0	0	0	0	0	0	0	0	0
3-hydroxybutyryl-CoA dehydrogenase (EC 1.1.1.157)	0	21.9502	9.67113	0	0	0	0	12.192124	74.75293	0	0	0	0.288509
3-hydroxypropionate dehydrogenase (EC 1.1.1.298)	0	0	0	21.398	0	13.66673	0	0	0	0	0	0	0
3-isopropylmalate dehydratase large subunit (EC 4.2.1.33)	3724.48	0	1956.54	0	0.52008	0.217712	0	394.372	206.424	0	1.24739	0.607022	0.924337
3-ketoacyl-CoA thiolase (EC 2.3.1.16)	0	41.71	20.8927	0	0	0	0	0	0	0	0	0	0
3-oxoacyl- reductase (EC 1.1.1.100)	9172.431	461.965	4399.805	0	0	0	0	358.30739	6.852902	186.066	4.31558	75.034311	9.432723
3-oxoacyl- synthase, KASIII (EC 2.3.1.180)	1916.312	0	0.174099	0	0.352081	0.147386	0	0	1687.879	0	0	0	0
3,4-dihydroxy-2-butanone 4-phosphate synthase (EC 4.1.99.12) / GTP cyclohydrolase II (EC 3.5.4.25)	0	0	0	0	0	0	530.229	0	0	0	0	0	0
3,7-dideoxy-D-threo-hepto-2, 6-diulosonate synthase (EC 4.2.3.-)	0	0	0	0	0	0	69.2628	0	33.9747	0	0	0	0
308aa long conserved hypothetical protein	0	214.481	101.31	0	0	0	0	0	0	0	0	0	0
4-carboxy-2-hydroxymuconate-6-semialdehyde dehydrogenase	0	670.407	516.269	0	0	0	0	0	0	0	0	0	0
4-hydroxybutanoyl-CoA dehydratase (EC 4.2.1.-) / Vinylacetyl-CoA Delta-isomerase (EC 5.3.3.3)	0	0	0	0	0	0	0	0.730675	0.371921	0	0	0	0
4-hydroxybutyrate coenzyme A transferase	0	0	0	0	0	0	0	0	0	16.0793	0	8.2546	0
4-hydroxyphenylacetate 3-monooxygenase (EC 1.14.13.3)	0	9.34655	4.44467	0	0	0	0	1.20651	0.614126	0	0	0	0
4'-phosphopantetheinyl transferase superfamily domain protein	0	0	0	0	0	0	0	0	0	131.752	0	68.7307	0
4Fe-4S ferredoxin, iron-sulfur binding domain protein	0	0	0	0	0	0	37.4777	23.867	30.4699	0	0	0	0
5-enolpyruvylshikimate-3-phosphate synthase (EC 2.5.1.19)	0	0	0	4.80684	0	2.80182	0	0	0	0	0	0	0
5'-methylthioadenosine nucleosidase (EC 3.2.2.16)	0	144.4781	68.8432	0	0	0	0	0	0	0	0	0	1.19678
5'-nucleotidase (EC 3.1.3.5)	221.30659	71.58299	130.389911	0	0	0	0	49.5597	25.0922	545.414	0	281.093	0
6-phosphofructokinase (EC 2.7.1.11)	0	35.4513	16.8586	0	0	0	0	710.774	349.307	0	0	0	0
6-phosphogluconate dehydrogenase	0	0	0	0	0	0	0	0	0	8.72547	0	4.5413	0
6,7-dimethyl-8-ribityllumazine synthase (EC 2.5.1.9)	0	0	0	0	0	0	0	0	0	0	0.249479	0.121404	0
60S ribosomal protein L14/L17/L23	0	0	0	0	0	0	0	794.377	405.063	0	0	0	0
67 kDa myosin-crossreactive antigen	0	0	0	0	8145.69	3417	0	0	0	0	0	0	0
7,8 dihydropteroate synthase (methanopterin)	0	0	0	0	0	0	0	18.80873	9.87303	0	0	0	0
8-amino-7-oxononanoate synthase	0	0	0	0	0	0	0	0.24569	0.125058	0	0	0	0
AA3-600 quinol oxidase subunit I	0	0	0	0	0	0	0	0	0	1361.62	0	699.255	0
AA3-600 quinol oxidase subunit II	0	0	0	0	0	0	0	0	0	645.082	0	333.684	0
ABC superfamily (ATP-binding membrane) transport protein	0.190361	0	0.0998364	0	0	0	0	0	0	0	0	0	0
ABC transporter	0	0	0	0	0	0	3.89478	0	1.45699	0	0	0	0
ABC transporter ATP-binding protein	42.2825	58.1533	49.8297	0	0	0	0	10.0828	5.26629	0.259585	0	0.133263	1.619427
ABC transporter multidrug efflux pump, fused ATP-binding domains	0	0	0	0	0	0	0	331.785	169.962	0	0	0	0
ABC transporter permease protein	41.1454	0	24.1844	0	0.275419	0.115294	0	0	0	0	0	0	0.629801
ABC transporter substrate-binding protein	0.156716	39.7823	19.1563709	0	0	0	64.9108	0	31.4574	335.459	0	173.166	1.734247
ABC transporter, ATP-binding protein EcsA	0	0	0	0	0	0	0	0	389.089	49.2331	0	27.0654	0
ABC transporter, nucleotide binding/ATPase protein	0	0	0	0	0	0	0	8.17208	4.15967	0	0	0	0
ABC transporter, periplasmic binding protein	0	0	0	2.83734	0	1.73207	0	0	0	0	0	0	0
ABC transporter, periplasmic spermidine putrescine-binding protein potD (TC_3.A.1.11.1)	0	0	0	0	0	0	0	101.487	52.3824	0	0	0	0
ABC transporter, periplasmic substrate-binding protein	0	0	0	0	9.80566	4.10478	0	67.1252	114.612	0	0	0	1.418443
ABC transporter, permease protein EscB	0	0	0	0	0	0	0	0	0	48.9842	0	25.1464	0
ABC transporter, substrate-binding protein, putative	0	0	0	0	0	0	0	0	0	3705.93	0	172.556	0
ABC-type dipeptide/oligopeptide/nickel transport system, permease component	0	123.151	59.4943	0	0	0	0	0	0	0	0	0	0
ABC-type polar amino acid transport system protein, substrate-binding protein	0	0	0	97.9242	0	60.6329	0	0	0	0	0	0	0
ABC-type sugar transport system, periplasmic binding protein YcjN	0	0	0	0	0	0	0	36.5448	18.6645	0	0	0	0
ABC-type sugar transport system, periplasmic component	0	177.201	84.6498	0	0	0	0	0	0	0	0	0	0
ABC-type sugar transport systems, ATPase components	4.96383	0	2.60332	0	0	0	0	0	0	0	0	0	0
ABC-type transport system, involved in lipoprotein release, permease component	0	0	0	0	0	0	4.36639	0	2.40067	0	0	0	0
ABC-type transport system, involved in lipoprotein release, permease component	0	0	0	0	0	0	7.98333	0	0	0	0	0	0
ABC-type tungstate transport system, periplasmic binding protein	0	0	0	0	0	0	0	31.5193	17.32291	0	0	0	0
ABC1 family protein	94.7717	0	48.9627	0	0	0	0	0	0	0	0	0	0
Accessory colonization factor AcfD precursor	0	0	0.157601	0	0	0	0	0	0	0	0	0	0
Accessory regulator Y	27.9435	0	14.65523	0	0	0	0	0	0	0	0	0	0
Acetate kinase (EC 2.7.2.1)	0	0	0	0	0	0	126.429	0	63.228	0	0	0	0
Acetoacetate metabolism regulatory protein AtoC	0	0	0	0	0	0	0	2.005626	1.020884	0	0	0	0
Acetoacetyl-CoA synthetase (EC 6.2.1.16) / Long-chain-fatty-acid--CoA ligase (EC 6.2.1.3)	0	0.289181	0.183357	0	0	0	0	0	0	0	0	0	0
Acetoin utilization protein	0	87.2774	41.4701	0	0	0	0	0	0	0	0	0	0
Acetolactate synthase, catabolic (EC 2.2.1.6)	0	0	0	0	0	0	0	0	0	38.159	0	19.7895	0
Acetolactate synthase, large subunit (EC 2.2.1.6)	0.174806	0	0.0916785	0	0	0	0	0	0	0	0	0	0
Acetyl-CoA acetyltransferase (EC 2.3.1.9)	12.25232	0	6.42583	0	0	0	0	0	0	0	0	0	0.485675
Acetyl-CoA synthase corrinoid activation protein	0	0	0	0	0	0	0	0.582454	0.296474	0	0	0	0
Acetyl-CoA synthase corrinoid iron-sulfur protein, small subunit	0	0	0	0	0	0	0	141.569711	71.895247	0	0	0	0
Acetyl-CoA synthetase (ADP-forming) alpha and beta chains, putative	0	598.26	284.401	0	0	0	0	0.904379	0.460337	0	0	0	0.452732
Acetyl-coenzyme A carboxyl transferase alpha chain (EC 6.4.1.2)	754.552	78.7091	434.7941	0	0	0	0	87.5269	45.3336	0	0	0	0.203939
Acetylglutamate kinase (EC 2.7.2.8)	0	0	0	0.211075	0	0.122716	0	0	0	0	0	0	0
Acetylornithine aminotransferase (EC 2.6.1.11)	0	1367.56	650.94	0	0	0	0	0	0	0	0	0	0
Acetyltransferase	0	0	0	0	0	0	0	0	0	0	19.7836	9.62735	0
Acetyltransferase (EC 2.3.1.-)	0	0	0	0	0	0	0	0	0	39.9667	0	20.4895	0
Aconitate hydratase (EC 4.2.1.3)	0	17.9524	8.53711	0	0.275419	0.115294	0	0	0	0	0	0	2.227253
Aconitate hydratase X, predicted	0	0	0	0	0	0	0	47.1329	30.1792	0	0	0	0
Acriflavin resistance protein B	0	0	0	0	0	0	0	7.47377	3.54186	0	0	0	0
Actin and related proteins	0	0	0	0	0	0	0	0	0	284.349	0	148.949	0
Activator of (R)-2-hydroxyglutaryl-CoA dehydratase	0	0	0	0	0	0	0	1041.78	537.715	0	0	0	0
Acyl---UDP-N-acetylglucosamine O-acyltransferase (EC 2.3.1.129)	0	24.0093	10.7687	0	0	0	0	209.804	107.193	0	0	0	0
Acyl-ACP thioesterase	0	0	0	0	0	0	0	0	0	0	83.0881	40.3664	0
Acyl-CoA synthetases (AMP-forming)/AMP-acid ligases	0	0.978881	0.465498	0	0	0	0	0	0	0	0	0	0
Acylamino-acid-releasing enzyme (EC 3.4.19.1)	0	305.838	145.9154	0	0	0	0	0	0	0	0.270078	0.131429	0
Adenine specific DNA methylase (Mod-related)	0	0	0	0	0	0	0	36.9	18.32	0	0	0	0
Adenosine (5')-pentaphospho-(5'')-adenosine pyrophosphohydrolase (EC 3.6.1.-)	0	42.3854	20.156	0	0	0	0	0	0	0	0	0	0
Adenosine deaminase (EC 3.5.4.4)	0	0	0	31.2635	0	18.1762	0	0	0	0	0	0	0.620467
Adenosylhomocysteinase (EC 3.3.1.1)	0	0	0	0	214.282	88.1118	0	108.0423	55.0666	0	0	0	5.155449
Adenosylmethionine-8-amino-7-oxononanoate aminotransferase (EC 2.6.1.62)	752.89	0	343.009	0	0	0	0	0	0	0	0	0	1.724995
Adenylate cyclase (EC 4.6.1.1)	0.465201	0	0.243979	5.78946	0	5.06622	0	0	0	0	0.285811	0.139085	0.597103
Adenylosuccinate lyase (EC 4.3.2.2)	0	0	0	283.296	0	164.6302	0	0	0	0	0	0	0.947729
Adenylylsulfate kinase (EC 2.7.1.25)	0	99.9871	46.918	0	0	0	0	5.79513	3.74868	0	0	0	0
Adenylylsulfate reductase beta-subunit (EC 1.8.99.2)	0	0	0	258.571	0	155.409	0	0	0	0	0	0	0
Adhesin of unknown specificity SdrC	0	0	0	0	0	0	0	0	0	0	63.0825	30.5569	0
ADP-heptose--lipooligosaccharide heptosyltransferase II (EC 2.4.1.-)	0	0	0	151.7152	0	81.9094	0	0	0	0	0	0	0.596611
ADP-heptose:LPS heptosyltransferase II	0	0	0	0	0	0	0	15.5509	7.91554	0	0	0	0
ADP-ribosylglycohydrolase (EC 3.2.-.-)	0	0	0	0	0	0	0	1.11677	0.568447	0	0	0	0
AIR Synthase, TMP kinase, hydrogenase maturation protein HypE superfamily	0	0	0	0	0	0	37.4362	0	18.0252	0	0	0	0
Alanine dehydrogenase (EC 1.4.1.1)	0	459.685	217.615	0	0	0	0	0	0	0.439455	0	0.225602	0
Alanyl-tRNA synthetase (EC 6.1.1.7)	13.5028	22.005988	17.576621	0	0	0	0	1.18643	0.629066	250.435	0	124.3673	0.731963
Aldehyde dehydrogenase (EC 1.2.1.3)	31.735252	0	24.6907743	0	0	0	0	0	0	0	0	0	3.932351
Aldehyde dehydrogenase B (EC 1.2.1.22)	0	14.9438	7.03041	0	0	0	0	0	0	0	0	0	2.798934
Aldehyde ferredoxin oxidoreducatse (EC 1.2.7.-)	0	3.66107	1.74099	0	0	0	36.7642	0	16.4287	0	0	0	0
Aldose 1-epimerase (EC 5.1.3.3)	0	0	0	0	0	0	0	0	0	5.15936	0	2.69471	0
Aldose-ketose isomerase YihS	0.172789	0	0.0906207	0	0	0	0	0	0	0	0	0	0
Aldoxime dehydratase	0.250698	0	0.131481	0	0	0	0	0	0	0	0	0	0
Alkaline shock protein 23	0	0	0	0	0	0	0	0	0	84.0196	0	43.382	0
Alpha-acetolactate decarboxylase (EC 4.1.1.5)	0	0	0	0	0	0	0	0	0	3.48818	0	1.79072	0
Alpha-galactosidase (EC 3.2.1.22)	0	0	0	0	0	0	0	226.3236	116.1464	0	0	0	0
Alpha-glucosides-binding periplasmic protein AglE precursor	0	0	0	1.417689	0	0.824225	0	231.83538	116.536295	0	0	0	0
Alternative dihydrofolate reductase 3	0	29.8316	14.1862	0	0	0	0	0	0	0	0	0	0
Amidohydrolase AmhX	0	0	0	1.3292	0	0.772781	0	12.6135	6.28083	44.1554	0	22.6506	0
Amidophosphoribosyltransferase (EC 2.4.2.14)	0.192812	0	0.101122	0	0	0	865.629	0	443.2932	0	0	0	0
Amino acid ABC transporter, periplasmic amino acid-binding protein	0	0	0	0	0	0	0	0	0	51.1367	0	26.8249	0
Amino acids ABC transporter, permease protein	0	0	0	107.155	0	62.8665	0	0	0	0	0	0	0
Aminopeptidase N	0	0	0	0	0	0	90.4851	0	44.5733	0	0	0	0
Aminopeptidase YpdF (MP-, MA-, MS-, AP-, NP- specific)	0	0	0	0	0	0	0	29.7143	15.1249	0	0	0	0

Anaerobic C4-dicarboxylate transporter DcuB	0	0	0	0	0	0	0	0	0	0.214657	0	0.110198	0
Anaphase-promoting complex (APC), Cdc16 subunit	0	5.76781	2.74283	0	0	0	0	0	0	0	0	0	0
Anhydro-N-acetylmuramic acid kinase (EC 2.7.1.-)	314.004	0	163.8117	0	0	0	0	0	0	0	0	0	0.984643
Arabinan endo-1,5-alpha-L-arabinosidase A precursor(EC:3.2.1.99)	0.224626	0	0.117807	0	0	0	0	0	0	0	0	0	0
Archaeal DNA polymerase I (EC 2.7.7.7)	0	186.5248	88.8695	0	0	0	93.8248	23.1537	52.20475	0	0	0	0
Archaeal DNA polymerase II large subunit (EC 2.7.7.7)	0	0	0	0	0	0	618.456	0	303.202	0	0	0	0
Archaeal transcription factor E	0	63.7771	30.3121	0	0	0	0	0	0	0	0	0	0
Archaeosine tRNA-ribosyltransferase (EC 2.4.2.-) type 1 / archaeosine tRNA-ribosyltransferase (EC 2.4.2.-) PU	0	0	0	0	0	0	0	6.37487	3.28163	0	0	0	0
Arginine deiminase (EC 3.5.3.6)	0.278462	0	0.146042	0	0	0	0	0	0	0	0	0	0
Argininosuccinate synthase (EC 6.3.4.5)	0	49.657	23.7192	0	0	0	0	0	0	0	0	0	0
Aromatic hydrocarbon utilization transcriptional regulator CatR (LysR family)	0	15.293	7.20662	0	0	0	0	0	0	0	0	0	0
Arsenic resistance protein ArsH	0	37.5215	17.843	47.0642	0	27.3625	0	0	0	0	0	0	0
Arsenical pump-driving ATPase (EC 3.6.3.16)	0	0	0	39.9592	0	23.4061	0	0	0	0	0	0	0
Arsenical resistance operon trans-acting repressor ArsD	0	0	0	58.4566	0	33.9859	0	0	0	0	0	0	0
Arylmalonate decarboxylase	0	0	0	0	0	0	0	1.5427	0.78525	0	0	0	0
Asparagine synthetase (EC 6.3.5.4)	0	0	0	0	0.531547	0.222513	0	0	0	0	0	0	0
Aspartate aminotransferase	0	0	0	0	0	0	0	15.3634	7.78844	0	0	0	0
Aspartate ammonia-lyase (EC 4.3.1.1)	0	546.866	259.741	0	0	0	0	0	0	0	0	0	0.976288
Aspartate carbamoyltransferase (EC 2.1.3.2)	0.87403	2.68942	2.644123	0	0	0	0	0	0	0	0	0	0
Aspartate-semialdehyde dehydrogenase (EC 1.2.1.11)	0	67.403	31.2199	0	0	0	0	5.43757	2.7183	0	0	0	0.241903
Aspartokinase (EC 2.7.2.4)	0	103.9696	49.159	0	0	0	0	64.1796	32.8704	0	0	0	0.806974
Aspartyl-tRNA synthetase (EC 6.1.1.12)	0	0	0	0	0	0	846.743	4.84292	420.96384	0	0	0	0
Aspartyl-tRNA(Asn) amidotransferase subunit B (EC 6.3.5.6)	5.91803	0	3.10376	0	0	0	0	0	0	0	0	0	0.559243
ASPIC/UnbV domain-containing protein	0	0	0	0	0	0	0	0	0	1.15206	0	0.591429	0
ATP phosphoribosyltransferase regulatory subunit (EC 2.4.2.17)	1418.87	0	735.279	0	0	0	0	0	0	0	0	0	0.382279
ATP synthase epsilon chain (EC 3.6.3.14)	0	0	0	0	0	0	0	175.980424	89.980633	0	0	0	0
ATP synthase F0 sector subunit a	0	0	0	0	0	0	0	0	0	38.5595	0	20.3402	0
ATP synthase F0 sector subunit b	0	0	0	0	0	0	0	0	0	1277.607	0	639.578	0
ATP synthase F0 sector subunit c	0	0	0	0	14.6746	6.14301	0	0	0	0	0	0	0
ATP-binding protein p271	0.190361	0	0.0998364	0	0	0	0	0	0	0	0	0	0
ATP-binding region, ATPase-like	0	0	0	0	0	0	0	6.46197	3.2892	0	0	0	0
ATP-dependent Clp protease ATP-binding subunit ClpA	2161.779	37.7572	1056.614738	0	0	0	0	0	0	0	0	0	293.003784
ATP-dependent Clp protease ATP-binding subunit ClpC	0	41.427659	19.865402	50.576	0	30.4241	0	284.90693	145.14963	0	0	0	0
ATP-dependent DNA helicase	0.3623	0	0.190011	0	0	0	0	0	0	0	0	0	0
ATP-dependent DNA helicase RecQ	55.9459	0	29.4149	0	0	0	0	0	0	0	83.069278	39.272929	0.956048
ATP-dependent DNA helicase SCO5183	0.185641	0	0.0973611	0	0	0	0	0	0	0	1.75433	0.96834	0
ATP-dependent DNA helicase UvrD/PcrA, actinomycete paralog	0.208954	0	0.109588	0	0	0	0	0	0	0	0	0	0
ATP-dependent DNA ligase (EC 6.5.1.1) LigC	0	0	0	0	0	0	0.370931	0	0.182124	0	0	0	0
ATP-dependent DNA ligase I	0	0.212646	0.101122	0	0	0	0	0	0	0	0	0	0
ATP-dependent helicase	0	0	0	0	0	0	0	12.77586	6.50303	0	0	0	0
ATP-dependent helicase hrpA	0	0	0	0	0	0	0	16.4176	8.3567	0	0	0	0
ATP-dependent hsl protease ATP-binding subunit HslU	0	678.587	322.368	0	0	0	0	0	0	0	0	0	1.00632
ATP-dependent protease La (EC 3.4.21.53) Type I	0	127.028604	64.929277	0	0	0	0	0	0	15.5154	0	7.7932	1.099142
ATP-dependent protease La (EC 3.4.21.53) Type II	0	0	0	0	0	0	0	61.1312	41.9377	0	0	0	0
ATP-dependent RNA helicase	0	0	0	0	0	0	0	1373.6481	873.8974	0	0	1089.87	0
ATP-dependent RNA helicase A	0	0	0	0	0	0	0	14.0785	6.99584	0	0	0	0
ATP-dependent RNA helicase, DEAD box family	0	0	0	0	0	0	0	0	0	7.37814	0	3.78769	0
ATP-dependent RNA helicase, eIF-4A family	0	0	0	0	0	0	57.9112	0	29.1944	0	0	0	0
ATP/GTP-binding protein	0	0	0	0	0	0	0	0	0	0	0.262844	0.127908	0
ATPase component of ABC transporters with duplicated ATPase domains/Translation elongation factor EF-3	0	0	0	21.9959	0	12.6897	0	0	0	0	0	0	0
ATPase component of general energizing module of ECF transporters	0	0	0	0	0	0	0	0	0	0	246.96	2.19271	0
ATPase involved in DNA repair	0	0	0	0	0	0	56.265	0	27.5743	0	0	0	0
ATPase type IV secretion complex and secretion of T-DNA complex (VirB11)	0	0	0	0.400138	0	0.232635	0	0	0	0	0	0	0
ATPase YjeE	0.228047	0	0.119601	0	0	0	0	0	0	0	0	0	0
Autotransporter adhesin	0	0	0	0	0	0	0	0.866696	0.575422	0	0	0	0
Auxin-responsive GH3 protein homolog, putative	0	0	0	0	0	0	345.724	0	170.568	0	0	0	0
B12 binding domain of Methylmalonyl-CoA mutase (EC 5.4.99.2)	0	20.9318	15.9188	0	0	0	0	0	0	0	0	0	0
Bacillopeptidase F	162.187	0	0	0	0	0	0	0	0	0	0	0	0
Bacillosamine/Legionaminic acid biosynthesis aminotransferase PglE; 4-keto-6-deoxy-N-Acetyl-D-hexosamin	0	0	0	0	0	0	0	22.3221	11.4865	0	0	0	0
Bacterial proteasome-activating AAA-ATPase (PAN)	0	0	0	0	0	0	0	0	0	0.350277	0	0.179821	0
Bacterioferritin	297.261	93.9246	203.1818	0	0	0	0	0	0	0	0	0	0.353703
Barstar, RNAse (barnase) inhibitor	0	0	0	96.3939	0	56.0681	0	0	0	0	0	0	0
Beta N-acetyl-glucosaminidase (EC 3.2.1.52)	3539.59	0	1974.473	0	0	0	0	0	0	0	0	0	0
Beta-adrenergic receptor kinase 1 (EC 2.7.11.15)	0	0	0	0	10033.5	0	0	0	0	0	943.903	2097.56	0
Beta-carotene ketolase (EC 1.14.-.-)	0	0	0	0	0	0	0	2.056767	1.031764	0	0	0	0
Beta-galactosidase (EC 3.2.1.23)	0	0	0	0	0	0	4.22643	76.4276	40.977579	0	1.95552	0.951619	0
Beta-glucanase precursor (EC 3.2.1.73)	0	0	0	0.198309	0	0.115294	0	0	0	0	0	0	0
Beta-glucoside ABC transport system, sugar-binding protein	0	0	0	0	0	0	1427.45	0	708.129	0	0	0	0
Beta-hexosaminidase (EC 3.2.1.52)	0	0	63.4776	0	0	0	0	0	0	0	0	0	0
Beta-hexosaminidase precursor	0	0	0	0	0	0	0	0	0	43.8178	0	22.4745	0
Beta-lactamase (EC 3.5.2.6)	0	0	0	322.7954	0	187.5779	0	302.831	153.0783	0	0	0	0.733281
Beta-ureidopropionase (EC 3.5.1.6)	0	0	0	2.96507	0	3.47068	0	0	0	0	0	0	0.626983
BH2577 unknown conserved protein	0	0	0	0	0	0	0	0	0	12.2016	0	6.26388	0
Bifunctional autolysin Atl / N-acetylmuramoyl-L-alanine amidase (EC 3.5.1.28)/ endo-beta-N-acetylglucosam	0	0	0	0	0	0	0	0	0	421.708	0	258.2133	0
Biofilm PGA outer membrane secretin PgaA	0	7.62646	3.6267	0	0	0	0	0	0	0	0	0	0
Biosynthetic arginine decarboxylase (EC 4.1.1.19)	0	0	0	0	0	0	0	0	0	0	0.285811	0.139085	0
Biotin synthesis protein BioC	9.32506	0	4.89061	0	0	0	0	0	0	0	0	0	0
Biotin--protein ligase (EC 6.3.4.9, EC 6.3.4.10, EC 6.3.4.11, EC 6.3.4.15)	0	0	0	0	0	0	0	18.68459	9.28867	0	0	0	0
Bipolar DNA helicase HerA	0	0	0	0	0	0	0	460.903	253.178	0	0	0	0
Blr3456; hypothetical protein	0	0	0	0	0	0	0	15.34	8.28432	0	0	0	0
Branched-chain amino acid transport ATP-binding protein LivG (TC 3.A.1.4.1)	1165.31	0	616.344	0	0	0	621.674	0	304.796	0	0	0	5.345865
Branched-chain amino acid transport system carrier protein	8.36982	0	4.38962	0	0	0	0	0	0	0	0	0	0
C4-dicarboxylate transport sensor protein	211.2211	0	46.7902	0	0	0	0	0	0	0	0	0	0
Calmodulin-binding protein CRAG, contains DENN domain	61.9657	0	33.1626	0	0	0	0	0	0	0	0	0	0
cAMP-binding proteins - catabolite gene activator and regulatory subunit of cAMP-dependent protein kinas	0	0	0	0	0	0	0	0	0	0	0.347699	0.169202	0
Carbamate kinase (EC 2.7.2.2)	0	0	0	0	0	0	0	56.9802	29.0035	0	0	0	0
Carbon monoxide dehydrogenase CooS subunit (EC 1.2.99.2)	0	0	0	0	0	0	0	6.11056	3.26342	0	0	0	0
Carbon-nitrogen hydrolase	0	0	0	0	0	0	0	0.231783	0.11798	0	0	0	0
Carbonic anhydrase (EC 4.2.1.1)	0.262209	0	0.137518	0	0	0	0	0	0	0	0	0	0
Carboxy-terminal processing protease	0	0	0	16.31899	0	9.48765	0	0	0	0	0	0	0
CarD-like transcriptional regulator	0	0	0	0	0	0	0	0	0	830.474	0	425.895	0
Cardiolipin synthetase (EC 2.7.8.-)	0	0	0	499.677	0	299.56	0	0	0	0	0	0	0.673123
Cartilage oligomeric matrix protein	0	248.3424	117.9314	0	0	0	0	0	0	0	0	0	0
Catalase (EC 1.11.1.6)	0	30.9961	14.5709	0	0	0	0	0	0	0	0	0	2.339523
Catechol 1,2-dioxygenase (EC 1.13.11.1)	0	0	0	0	5.48466	2.29595	0	0	0	0	0	0	0
Cation transport ATPase	0	0	0	0	0	0	0	0	0	0.270926	0	0.139085	0
Cation-transporting ATPase, E1-E2 family	0	0	0	0	0	0	0	0	0	56.6446	0	29.0795	0
CBS domain protein	0	0	0	0	9.39751	3.93392	0	0	0	0	0	0	0.529884
CBS domain protein	0	0	0	0	0	0	0	0	32.788	0	0	0	0
CcsA-related protein	53.2671	0	28.163	0	0	0	0	0	0	0	0	0	0.161269
CDP-6-deoxy-delta-3,4-glucoseen reductase-like	0	0	377.868	0	0	0	0	0	0	0	0	0	0.445011
Cell division control protein CDC48 homolog	0	0	0.262962	0	0	0	0	0	0	0	0	0	0
Cell division inhibitor related protein	0	0	0	0	0	0	1.25452	0	0.615959	0	0	0	0
Cell division protein FtsI (EC 2.4.1.129)	219.7134	0	114.9104	0	0	0	0	1.44209	0.734038	14.6222	96.67653	55.013189	0.472105
Cell division protein FtsJ / Ribosomal RNA large subunit methyltransferase E (EC 2.1.1.-)	0	7.93754	3.77463	0	0	0	0	0	0	0	0	0	0
Cell division protein FtsW	0	6.41138	3.04888	0	0	0	0	0	0	0	0	0	0
Cell division protein FtsZ (EC 3.4.24.-)	0	180.99462	85.92239	0	0	0	1277.97	1060.6015	1137.0426	0	0	0	0.475287
Cell division protein MraZ	542.322	218.3413	405.7908	0	0	0	0	0	0	0	0	0	0.216134
Cell surface glycoprotein related protein	0	0	116.304	0	0	0	0	0	0	0	0	0	0
Cell surface protein	0	657.1572	312.1306	0	0	0	0	0	0	0	0	0	0
Cell wall/surface repeat protein	0	0	0	0	0	0	0	100.851	51.334	0	0	0	0
Cellobiohydrolase A (1,4-beta-cellobiosidase A)	0	5.55771	0	0	0	0	0	0	0	0	0	0	0
Cellulose synthase catalytic subunit (EC 2.4.1.12)	0.170818	0	0.089587	0	0	0	0	0	0	0	0	0	0
central glycolytic regulator	0	0	0	0	0	0	0	0	0	1.99324	0	1.02327	0
Chaperone HdeB	13.3407	0	6.99665	0	0	0	0	0	0	0	0	0	0
Chaperone protein DnaK	0	320.524116	152.650589	0	0	0	47.6508	96.6306	72.7495	27.9054	0	14.32573	1.691018
Chaperonin complex component, TCP-1 epsilon subunit (CCT5)	0	0	0	0	0	0	0	259.4	133.253	0	0	0	0
CheA signal transduction histidine kinase 2	0	0	0	0	0	0	0	0	0	181.7524	0	93.4135	0
Chemotaxis protein methyltransferase CheR (EC 2.1.1.80)	18.9384	0	9.9324	0	0	0	0	0	0	0	0	0	2.156392
Chemotaxis response regulator protein-glutamate methylesterase CheB (EC 3.1.1.61)	0	7.99499	4.23034	0	0	0	0	0	0	0	0	0	0.954122
Chitinase (EC 3.2.1.14)	42.8555	198.011	121.6435	0	0	0	0	0	0	0	0	0	0
ChlI component of cobalt chelatase involved in B12 biosynthesis	0	0	0	0	0	0	0	4.23247	2.15437	762.103	0	391.677	0
Chloramphenicol acetyltransferase (EC 2.3.1.28)	0	0	0	0	0	0	172.7984	1.07067	85.492869	0	0	0	0
Chromosomal replication initiator protein DnaA	0	63.9244	30.3987	0	0	0	0	0	0	46.8829	0	24.0221	2.622765
Chromosome (plasmid) partitioning protein ParA	0	0	0	0	340.452	120.258	0	0	0	0	0	0	2.825392
Chromosome (plasmid) partitioning protein ParB-2	0	0	0	0	0	0	0	0	68.7394	0	0	0	0
Chromosome partitioning protein parB / Adenine-specific methyltransferase(EC:2.1.1.72)	0	0	0	0	0	0	81.8589	0	0	0	0	0	0
Circadian phase modifier	0	0	0	0	0	0	0	25.4563	12.6752	0	0	0	0
Circumsporozoite protein	0	0	0	8.55819	0	4.97562	4.95697	0	2.42421	0	0	65.835	0

Citrate lyase gamma chain, acyl carrier protein (EC 4.1.3.6)	0.218083	0	0.114376	0	0	0	0	0	0	0	0	0	0
Clostripain	0	86.1892	40.83197	0	0	0	0	0	0	0	0	0	0
CO dehydrogenase accessory protein CooC (nickel insertion)	0	0	0	0	0	0	14.5714	0	6.09947	0	0	0	0
CO dehydrogenase/acetyl-CoA synthase subunit alpha, CO dehydrogenase subcomplex (EC 1.2.99.2)	0	0	0	0	0	0	0	619.437	315.496	0	0	0	0
CO dehydrogenase/acetyl-CoA synthase subunit beta, acetyl-CoA synthase (EC 2.3.1.169)	0	3.6825	1.75118	0	0	0	0	39.966016	20.343119	0	0	0	0
CoB--CoM heterodisulfide reductase 2 iron-sulfur subunit D (EC 1.8.98.1)	0	0	0	0	0	0	0	163.381	83.6148	0	0	0	0
CoB--CoM heterodisulfide reductase subunit A (EC 1.8.98.1)	0	109.046	53.1183	0	0	0	0	157.541624	87.241651	0	0	0	0
CoB--CoM heterodisulfide reductase subunit C (EC 1.8.98.1)	0	2116.92	949.974	0	0	0	0	98.7043	50.2415	0	0	0	0
CoB--CoM heterodisulfide reductase subunit D (EC 1.8.98.1)	0	6.66971	3.17172	0	0	0	0	25.4299	12.7755	0	0	0	0
Cobalamin B12-binding domain-containing protein	0	0	0	0	0	0	0	361.863	184.126	0	0	0	0
Cobalamin biosynthesis protein CbiG	478.015	0	0	0	0	0	0	97.6544	10.3891	0	0	0	0
Cobalt-precorrin-6y C5-methyltransferase (EC 2.1.1.-)	0	101.226	49.4902	0	0	0	0	0	0	0	0	0	0
Cobalt-zinc-cadmium resistance protein CzcA; Cation efflux system protein CusA	5492.1399	0	2853.465707	708.336839	1726.523	1144.12327	0	205.0753	104.3854	1412.473	0	733.922	18.254446
Cobyrinic acid A,C-diamide synthase	0	0	0	0	0	0	0	221.888	106.97	0	0	0	0
Cocaine esterase (EC 3.1.1.-)	0	0	0	9.12872	0	5.30731	0	0	0	0	0	0	0
Cold shock protein CspA	0	0	0	0	0	0	0	0.491379	0.250117	0	0	0	0
Cold shock protein CspC	0	0	0	0	0	0	0	0	0	0	82.6314	40.1905	0
Cold shock protein CspG	492.941	0	258.851	0	0	0	0	0	0	0	0	0	0
cold-active alkaline serine protease	0	0	0.992224	0	0	0	0	0	0	0	0	0	0
Colicin I receptor precursor	0	0	0	0	0	0	5.43563	0	2.7048	0	0	0	0
Colicin V production protein	0.172789	0	0.0906207	0	0	0	0	0	0	0	0	0	0
Colicin V secretion protein	0	0	0	0	0	0	0	0	0	0	8.62665	2.03126	0
Competence protein F	0	0	0	0	0	0	0	0	0	3.32882	0	1.78163	0
Conjugative transfer protein TrbB	0	16.5813	7.5096	0	0	0	0	0	0	0	0	0	0.517139
Conserved hypothetical membrane spanning protein, MFS member	0	0	0	59.7043	0	34.7719	0	0	0	0	0	0	0
Copper amine oxidase domain protein	0	0	0	0	0	0	0	55.0772	28.3284	0	0	0	0
Creatinine amidohydrolase, putative	22.4103	0	11.7533	0	0	0	0	0	0	0	0	0	0
CRISPR-associated protein, Cse4 family	0.399432	0	0.2094855	0	0	0	0	0	0	0	0	0	0
Cyanate hydratase (EC 4.2.1.104)	12476.9	0	0	0	0	0	0	0	0	0	0	0	0.585977
Cyclin	0	0	0	0	760.114	314.179	0	0	0	0	0	0	0
Cys regulon transcriptional activator CysB	5.31135	0	5.39706	0	0	0	0	0	0	0	0	0	0.487117
Cys repeats; part of IE-A	0	0	0	0	0	27867.1	0	0	0	0	0	0	0
Cystathionine gamma-lyase (EC 4.4.1.1)	0	0	0	0	0	0	0	25.5037	12.9646	0	0	0	0
Cysteine desulfurase (EC 2.8.1.7)	0	15.7104	7.51644	0	0	0	4.8326	25.4076	15.25716	0	0	0	2.318982
Cysteine desulfurase (EC 2.8.1.7), IscS subfamily	0	0	0	66.0006	5456.43	2324.6126	0	0	0	0	0	0	86.846609
Cysteine desulfurase CsdA-CsdE (EC 2.8.1.7), main protein CsdA	0.256227	0	0.134381	0	0	0	0	0	0	0	0	0	0
Cysteine synthase (EC 2.5.1.47)	0	0	0	3.13623	0	1.82336	0	0	0	0	0	0	0.150466
Cysteinyl-tRNA synthetase related protein	0	0	0	0	0	0	0	0	0	2.94045	0	1.50953	0
Cystine ABC transporter, permease protein	0	0	0	115.111	0	66.9243	0	0	0	0	0	0	0
Cytidine deaminase (EC 3.5.4.5)	0	102.4171	48.8056	0	0	0	0	0	0	0	0	0	0
Cytochrome c family protein	0	8.43446	4.30889	0	0	0	0	0	0	0	0	0	0
Cytochrome c oxidase (EC 1.9.3.1)	0	122.9166	58.452	0	0	0	0	0	0	0	0	0	1.188795
Cytochrome c-type biogenesis protein DsbD, protein-disulfide reductase (EC 1.8.1.8)	59.1386	0	30.7517	0	0	0	0	0	0	0	0	0	0.57526
Cytochrome cd1 nitrite reductase (EC:1.7.2.1)	53.7166	0	28.1315	422.984	0	244.984	0	0	0	0	0	0	0
Cytochrome O ubiquinol oxidase subunit I (EC 1.10.3.-)	0	0	0	0	142.736	59.7513	0	0	0	0	0	0	1.814203
D-alanine--poly(phosphoribitol) ligase subunit 1 (EC 6.1.1.13)	0	0	0	0	0	0	0	0	0	2.42082	0	1.24277	0
D-cysteine desulfhydrase (EC 4.4.1.15)	0	0	0	0	0	0	0	523.762	268.547	0	0	0	0
D-glycero-D-manno-heptose 1,7-bisphosphate phosphatase (EC 3.1.1.-)	0	129.59	61.8486	0	0	0	0	0	0	0	0	0	0
D-serine/D-alanine/glycine transporter	0	0	0	0	0.39866	0.166885	0	0	0	0	0	0	0
D-tyrosyl-tRNA(Tyr) deacylase	0	0	0	0	0	0	0	0	0	0.27493	0	0.14114	0
DcrB protein precursor	2.59177	0	1.359279	0	0	0	0	0	0	0	0	0	0
DEAD-box ATP-dependent RNA helicase CshA (EC 3.6.4.13)	0	31.96523	14.747611	0	0	0	0	0	0	0	0	0	0.444173
Deblocking aminopeptidase (EC 3.4.11.-)	0	0	0	0	0	8.92495	0	0	0	0	0	0	0
Degenerated pectate lyase	0	0	0	89.1509	0	52.9748	0	0	0	0	0	0	0
DegV family protein	0	9.24447	4.39613	0	0	0	0	0	0	0	0	0	0.392113
Deoxyribodipyrimidine photolyase (EC 4.1.99.3)	105.5563	0	54.9609	0	0	0	0	0	0	0	0	0	0
Deoxyribonuclease TatD	0	0	0	0	2297.21	960.837	0	0	0	0	0	0	0
Diacylglucosamine hydrolase like	0	598.028	284.489	0	0	0	0	0	0	0	0	0	0.201586
Diacylglycerol kinase (EC 2.7.1.107)	0	0	0	0	0	0	14.9922	0	7.36102	50.1682	0	25.8074	0
Diaminopimelate epimerase (EC 5.1.1.7)	0	0	0	0	5.66575	2.37176	0	0	0	0	0	0	0.157631
Dienelactone hydrolase family protein	0	0	0	0	0	0	0	0	0	0	82.4278	40.1121	0
Dihydrolipoamide succinyltransferase component (E2) of 2-oxoglutarate dehydrogenase complex (EC 2.3.1.	0	0	0	0	0	0	0	0	0	0.737588	0	0.378654	0
Dihydroorotate dehydrogenase, catalytic subunit (EC 1.3.3.1)	0	0	0	0	0.341519	0.142964	0	25.159	12.8062	0	0	0	0
Dinitrogenase iron-molybdenum cofactor protein	0	0	0	0	0	0	82.0377	0	0	0	0	0	0
Dipeptide transport system permease protein DppB (TC 3.A.1.5.2)	4.55924	0	2.39113	72.3986	0	41.1023	13.8574	1086.6676	670.6921	78.3498	0	40.2911	2.377877
Dipeptide transport system permease protein DppC (TC 3.A.1.5.2)	0	129.434	60.6598	0	0	0	0	0	0	0	0	0	1.48146
Dipeptide-binding ABC transporter, periplasmic substrate-binding component (TC 3.A.1.5.2); Putative hemi	0	0	0	0	0	0	0	0.728969	0.371052	0	0	0	0
Dipeptidyl anminopeptidase	0	0	0	0	0	0	0	69.4578	35.3547	0	0	0	0
Disulfide bond formation protein	0.200559	0	0.105185	0	0	0	0	0	0	0	0	0	0
DNA cytosine methyltransferase family protein	0	112.937	53.7061	0	0	0	0	0	0	0	0	0	0
DNA double-strand break repair protein Mre11	0	0	0	0	0	0	0	2.74553	1.3975	0	0	0	0
DNA double-strand break repair Rad50 ATPase	0	0	0	0	0	0	0	1.99022	4.64602	0	0	0	0
DNA gyrase subunit A (EC 5.99.1.3)	85.1935	5.54019	47.66389	0	0	0	0	820.3108	445.80664	393.678	0	246.081	2.553965
DNA gyrase subunit B (EC 5.99.1.3)	0	0	0	10.64552	0	6.18915	0	0	0	0	0	0	1.757821
DNA ligase (EC 6.5.1.2), LigB	0.195327	0	0.102441	0	0	0	0	0	0	0	0	0	0
DNA polymerase I (EC 2.7.7.7)	27.1089	163.2468	93.8956	0	0	0	0	0	0	0	0	0	1.022783
DNA polymerase III beta subunit (EC 2.7.7.7)	27.3313	0	14.27611	0	0	0	0	0	0	0	0	0	0.706456
DNA polymerase III polC-type (EC 2.7.7.7)	0	0	0	0	0	0	0	0	0	125.529	0	66.9787	0
DNA polymerase IV (EC 2.7.7.7)	0	0	0	124.5968	0	80.9364	0	0	0	0	0	0	0.45576
DNA primase large subunit (EC 2.7.7.-)	0	4.645893	1.982821	0	0	0	0	0	0	0	0	0	0
DNA recombination and repair protein RecF	0	0	0	5.43519	0	3.15994	0	0	0	0	0	0	0
DNA repair protein RAD51	0	0	0	0	0	0	0	5.39891	2.7481	0	0	0	0
DNA replication helicase protein MCM	0	0	0	0	0	0	0	10.6569	5.86707	0	0	0	0
DNA replication licensing factor, MCM5 component	0	0	0	0	0	0	0	12.7485	6.00429	0	0	0	0
DNA replication protein DnaD	0	8.47084	4.37025	3.96009	0	2.302344	0	0	0	0	0	0	0
DNA topoisomerase I (EC 5.99.1.2)	0	0	0	0	0	0	0	31.0055	15.56066	0	0	0	0
DNA topoisomerase III, Burkholderia type (EC 5.99.1.2)	21.4097	0	11.22847	0	0	0	0	0	0	0	0	0	0.208261
DNA topoisomerase VI subunit A (EC 5.99.1.3)	0	3.72021	2.11087	0	0	0	0	0	0	0	0	0	0
DNA topoisomerase VI subunit B (EC 5.99.1.3)	0	0	0	0	0	0	0	76.8386	39.2041	0	0	0	0
DNA-binding protein	0	0	0	0	0	0	0	0	0	211.284	0	108.1179	0
DNA-binding protein MTH1615	0	392.267	186.291	0	0	0	0	20.6166	10.2765	0	0	0	0
DNA-binding protein, putative	0	13.0705	6.19751	0	0	0	0	0	0	0	0	0	0
DNA-directed RNA polymerase alpha subunit (EC 2.7.7.6)	0	71.6375	38.6583	0	0	0	452.418	16.6932	230.24999	511.1964	0	306.0613	0.20965
DNA-directed RNA polymerase beta subunit (EC 2.7.7.6)	0	80.8871	38.4652	0	0	0	0	0	0	223.6372	0	247.9501	1.269081
DNA-directed RNA polymerase beta' subunit (EC 2.7.7.6)	0	346.43422	168.50612	0	0	0	222.072	84.0569	151.2682	295.354	0.223867	296.270601	1.45993
DNA-directed RNA polymerase gamma subunit (EC 2.7.7.6)	0	0	0	0	0.634794	0.265733	0	0	0	0	0	0	0
DNA-directed RNA polymerase II largest subunit (EC 2.7.7.6)	0	388.867	305.3213	0	0	0	0	55.97691	39.51415	0	0	0	0
DNA-directed RNA polymerase II second largest subunit (EC 2.7.7.6)	0	0	0	0	0	0	0	0	5.31254	0	0	0	0
DNA-directed RNA polymerase III largest subunit (EC 2.7.7.6)	0	413.653	0	0	0	0	0	0	28.876	0	0	0	0
DNA-directed RNA polymerase III second largest subunit (EC 2.7.7.6)	0	47.3549	23.3122	0	0	0	0	0	0	0	0	0	0
DNA-directed RNA polymerase subunit A' (EC 2.7.7.6)	0	50.94289	24.21297	0	0	0	0	0	0	0	0	0	0
DNA-directed RNA polymerase subunit B (EC 2.7.7.6)	0	0	0	0	0	0	0	13.0994	69.23434	0	0	0	0
DNA-directed RNA polymerase subunit B'' (EC 2.7.7.6)	0	0	0	0	0	0	0	26.7836	6.5853	0	0	0	0
DNA/RNA helicase of DEAD/DEAH box family	0	0	0	0	0	0	0	0	0	133.5235	0	1136.469	0
DNA/RNA-binding protein Alba	0	0	0	0	0	0	0	618.3071	313.0799	0	0	0	0
DnaJ-class molecular chaperone CbpA	0	0	0	0	0	0	0	2.38168	1.2123	0	0	0	0
dNTP triphosphohydrolase, putative	0	0	0	22.3623	0	13.0011	0	0	0	0	0	0	0
Dolichol-phosphate mannosyltransferase (EC 2.4.1.83) in lipid-linked oligosaccharide synthesis cluster	0	0	0	0	0	0	0	0.734046	0.373636	0	0	0	0
Double-strand break repair protein MRE11	0	2.83122	1.7671	0	0	0	0	0	0	0	0	0	0
DoxX family protein	0	0	0	0	0	0	0	2.49431	1.269628	0	0	0	0
dTDP-Rha:A-D-GlcNAc-diphosphoryl polyprenol, A-3-L-rhamnosyl transferase WbbL	27.2359	0	12.0458	0	0	0	0	0	0	44.0709	0	22.4352	0
DUF1541 domain-containing protein	0	0	0	0	475.576	198.193	0	0	0	0	0	0	0
Efflux pump antibiotic resistance protein	0	0	0	0	0	0	0	0	853.219	0	0	0	0
Efflux transporter, RND family, MFP subunit	0	0	0	0	0	0	0	0	0	4149.75	0	2068.18	0
Electron transfer flavoprotein-ubiquinone oxidoreductase (EC 1.5.5.1)	0	0	0	0	1818.347	541.991	0	0	0	0	0	0	1.362787
Endo-1,4-beta-xylanase A precursor (EC 3.2.1.8)	0	0	0	0	0	0	0	0	0	0	204.711	0	0
Endonuclease IV (EC 3.1.21.2)	0	308.394	176.9	0	0	0	0	0	0	0	0	0	0.184713
Enolase (EC 4.2.1.11)	0	0	0	0	279.343	115.9024	0	0	0	0	0	0	1.220679
Enoyl- reductase (EC 1.3.1.10)	0	0	0	0	0	0	0.662531	0	0.325296	0	0	0	0
Enoyl-CoA hydratase (EC 4.2.1.17)	0.167008	358.567	171.6905888	0	0	0	0	0	0	0	0	0	7.021473
Enoyl-CoA hydratase/carnithine racemase	0	1.68908	0.803229	0	0	0	0	0	0	0	0	0	0
Error-prone repair homolog of DNA polymerase III alpha subunit (EC 2.7.7.7)	0.285541	0	0.149755	543.284	0	302.711	0	0	0	0	0	0	1.275188
Error-prone, lesion bypass DNA polymerase V (UmuC)	1.92536	1.05418	1.511076	0	0	0	0	0	0	0	0	0	0
Erythronate-4-phosphate dehydrogenase (EC 1.1.1.290)	0	0	0	0	0	0	0	0	0	47.4694	0	24.9203	0
Esterase/lipase/thioesterase family protein	0	0	0	186.0115	0	117.872	0	0	0	0	0	0	0
Ethidium bromide-methyl viologen resistance protein EmrE	0	0	0	0	0	0	2.04872	0	1.0059	0	0	0	0
Eukaryotic peptide chain release factor subunit 1	0	0	0	0	0	0	0	343.28187	171.6777	0	0	0	0
Eukaryotic translation initiation factor 2 gamma subunit	0	371.043	176.864	0	0	0	0	0	0	0	0	0	0
Eukaryotic translation initiation factor 2-alpha kinase 4 (GCN2)	0.284337	0	0.149123	0	0	0	0	0	0	0	0	0	0

Eukaryotic translation initiation factor 3 subunit 10, putative	0	0	0	0	0	0	0	228.32	309.17	0	0	0	0
Excinuclease ABC subunit A paralog of unknown function	0	0	0	0	0	0	0	0	75.6521	0	0	0	0
Exodeoxyribonuclease III (EC 3.1.11.2)	1488.156	0	812.33	0	0	0	0	0	0	0	0	0	0.306306
Exodeoxyribonuclease V gamma chain (EC 3.1.11.5)	0	1.56418	0.743832	0	0	0	0	0	0	0	0	0	0
Exonuclease SbcD	0	0	0	0	942.588	395.428	0	33.6111	17.2013	0	0	0	0
Exported zinc metalloprotease YfgC precursor	0	101.4962	48.6942	0	0	0	0	0	0	0	0	0	1.143381
Extensin-like protein	0.187971	0	0.0985832	0	0	0	0	0	0	0	0	0	0
Extracellular deoxyribonuclease PA3909 (required for catabolism of external DNA)	0	5.27695	2.08867	0	0	0	0	0	0	0	0	0	0
Extracellular Matrix protein PelA	0	700.935	339.54	0	0	0	0	0	0	0	0	0	2.431971
extracellular solute-binding protein, family 3	0	0	0	0	0	0	0	79.9303	42.5584	0	0	0	0
extracellular solute-binding protein, family 5	0	1969.93	933.49	0	0	0	0	718.588	389.209	0	0	0	0
FAD dependent oxidoreductase	0	0	0	0	0	0	26.0888	0	12.8094	0	0	0	0
FAD linked oxidase domain protein	0	0	0	0	0	0	0	62.3263	31.7247	0	0	0	0
Fap amyloid fiber secretin	0.297518	0	0.156036	0	0	0	0	0	0	0	0	0	0
Fe-S oxidoreductase	0	0	0	0	0	0	0	1068.26158	542.66259	0	0	0	0
Fe-S oxidoreductase	0	0	0	0	0	0	0	43.351	22.1436	0	0	0	0
Fe-S-cluster-containing hydrogenase components 2	0	0	0	0	0	0	106.588	0	52.2922	0	0	0	0
Ferredoxin	0	36.1667	16.7265	0	0	0	0	0	25.2663	0	0	0	0.21134
Ferredoxin / Ferredoxin--NADP(+) reductase, actinobacterial (eukaryote-like) type (EC 1.18.1.2)	0	0	0	0	0	0	0	0	0	0	277.233	128.428	0
Ferric iron ABC transporter, iron-binding protein	0	51.1962	24.346	0	0	0	0	0	0	0	0	0	0.704504
Ferric uptake regulation protein FUR	0	0	0	0	112.513	47.463	0	35.0438	17.0051	0	0	0	1.232019
Ferrichrome-iron receptor	963.909	0	506.362	1047.1548	0	622.26547	0	0	0	0	0	0	4.953707
Fibrillarin	0	13.3713	6.21074	0	0	0	0	0	0	0	0	0	0
Fibronectin type III domain protein	299.33	904.296	587.556	0	0	0	0	222.94773	114.11502	0	0	0	0
Flagellar biosynthesis protein FliC	0	0	0	0	0	0	1.3645	0	0.669955	0	0	0	0
Flagellar hook-basal body complex protein FliE	165.752	0	87.1201	0	0	0	0	0	0	0	0	0	1.93367
Flagellar motor protein	0	0	0	0	0	0	0	62.5277	31.8754	0	0	0	0
Flagellin FlaB1	0	0	0	0	0	0	0	501.39723	251.56083	0	0	0	0
Flagellum-specific ATP synthase FliI	0	0	0	544.741	0	332.527	0	6.22048	3.16629	0	0	0	0.843103
Flap structure-specific endonuclease (EC 3.-.-.-)	0	430.468	368.714	0	0	0	0	0	0	0	0	0	0
Flavodoxin reductases (ferredoxin-NADPH reductases) family 1	263.99	0	0	0	0	0	0	0	0	0	0	0	0.601019
Fmu (Sun) /eukaryotic nucleolar NOL1/Nop2p; tRNA and rRNA cytosine-C5-methylases	0	22.1189	13.5711	0	0	0	0	0	0	0	0	0	1.18779
Formaldehyde activating enzyme / D-arabino-3-hexulose 6-phosphate formaldehyde-lyase (EC 4.1.2.43)	0	0	0	0	0	0	0	0.470219	0.239346	0	0	0	0
Formamidopyrimidine-DNA glycosylase (EC 3.2.2.23)	0.174806	0	0.0916785	151.4319	0	89.2001	0	0	0	0	0	0	0.74894
Formate dehydrogenase chain D (EC 1.2.1.2)	0	0	0	3.0833	0	1.62965	0	0	0	0	0	0	0.295976
Formate dehydrogenase O alpha subunit (EC 1.2.1.2) @ selenocysteine-containing	19.4229	0	10.1325	0	0	0	0	51.3885	26.2527	0	0	0	0
Formate dehydrogenase O beta subunit (EC 1.2.1.2)	0	0	0	0	0	0	0	246.198	124.304	0	0	0	0
Formate efflux transporter (TC 2.A.44 family)	0	0	0	148.764	0	96.612	0	0	0	0	0	0	0
Formate--tetrahydrofolate ligase (EC 6.3.4.3)	0	0	0	0	0	0	0	971.884	503.982	0	0	0	0
Formiminotetrahydrofolate cyclodeaminase (EC 4.3.1.4)	0	0	0	0	315.602	167.519	0	0	0	0	0	0	0
Formylmethanofuran dehydrogenase (tungsten) operon gene F (polyferredoxin)	0	6.93195	3.08027	0	0	0	0	0	0	0	0	0	0
Fosmidomycin resistance protein	0	0	0	0	11.3546	6.92715	0	0	0	0	0	0	0.344201
Fructokinase (EC 2.7.1.4)	0	0	0.286461	0	0	0	0	0	0	558.024	0	298.615	0
Fructosamine kinase family protein, At3g61080 homolog	0	0	0	0	0	0	0	112.42	57.9529	0	0	0	0
Fructosamine-3-kinase (EC 2.7.1.-)	0	0	0	0	0	0	0	109.591	55.9015	0	0	0	0
Fructose-1,6-bisphosphatase, Bacillus type (EC 3.1.3.11)	0	0	0	0	0	0	0	22.8885	11.6505	0	0	0	0
Fructose-1,6-bisphosphatase, type V, archaeal (EC 3.1.3.11)	0	0	0	0	0	0	632.165	0	136.06	0	0	0	0
Fructose-bisphosphate aldolase class II (EC 4.1.2.13)	0	303.866	144.8924	0	0	0	0	0	0	111.91266	0.231799	57.673991	0.619044
Fumarate reductase, flavoprotein subunit precursor (EC 1.3.99.1)	0	0	0	0	0	0	4.67949	0	2.29758	0	0	0	0
Fusobacterium membrane protein	0	0	0	0	0	0	0	17.32111	7.5035	0	0	0	0
Gag/pol polyprotein	0	0	0	0	0	0	0	0	0	13.4397	0	6.89952	0
Galactitol utilization operon repressor	0	0	0	0	0	0	0	0	0	131.358	0	67.4351	0
Galactose-1-phosphate uridylyltransferase (EC 2.7.7.10)	0	0	0	0	0	0	0	186.972	97.0636	0	0	0	0
Galactose/methyl galactoside ABC transport system, permease protein MglC (TC 3.A.1.2.3)	0	47.3463	21.2791	0	0	0	0	0	0	0	0	0	0
Gamma-glutamyl phosphate reductase (EC 1.2.1.41)	0	0	0	189.7955	0	199.118	0	0	0	0	0	0	0.951294
Gamma-glutamyl-GABA hydrolase (EC 3.5.1.94)	0.197908	0	0.103795	0	0	0	0	0	0	0	0	0	0
Gamma-glutamyl-putrescine synthetase (EC 6.3.1.11)	442.576	0	233.113	0	0	0	0	0	0	0	0	0	0
GDP-mannose 4,6 dehydratase (EC 4.2.1.47)	0	198.832	96.3596	0	0	0	0	0	0	0	0	0	0
General secretion pathway protein A	0	0	0	0	0	0	0	8.63963	4.39766	0	0	0	0
General secretion pathway protein F	0	28.3479	13.4806	0	0	0	0	0	0	0	0	0	0.147641
Geranyl-CoA carboxylase biotin-containing subunit (EC 6.4.1.5)	0	0	0	0	0	0	0	0	0	0	0.428716	0.208627	0
Geranylgeranyl diphosphate synthase (EC 2.5.1.29)	0	0	0	0	38.7168	16.5082	0	0	0	0	0	0	0
Geranylgeranyl pyrophosphate synthase	0	0	0	0	41.3796	16.1897	0	0	0	0	0	0	0
Geranylgeranyl pyrophosphate synthetase (EC 2.5.1.29)	0	0	0	0	0	0	0	0	0	113.9523	0	58.5308	0
Gfa-like protein	0.183368	0	0.0961689	0	0	0	0	0	0	0	0	0	0
GGDEF domain/EAL domain protein	0	0	0.246458	0	0	0	0	0	0	0	0	0	0
Gll4423 protein	0	0	0	0	0	0	0	567.008	288.368	0	0	0	0
Gluconate permease	0.224626	0	0.117807	0	0	0	0	0	0	0	0	0	0
Gluconate utilization system Gnt-I transcriptional repressor	1.26669	0	0.664325	0	0	0	0	0	0	0	0	0	0
Gluconokinase (EC 2.7.1.12)	0	450.193	218.81	0	0	0	0	225.59	95.2343	0	0	0	0.238238
Glucose 1-dehydrogenase (EC 1.1.1.47)	0	0	0	0	0	0	0	51.6719	26.3015	0	0	0	0
Glucose ABC transport system, inner membrane component 1	0.395816	0	0.207589	0	0	0	0	0	0	0	0	0	0
Glucose dehydrogenase, PQQ-dependent (EC 1.1.5.2)	0.206079	0	0.16212	0	0	0	0	0	0	0	0	0	0
Glutamate Aspartate periplasmic binding protein precursor GltI (TC 3.A.1.3.4)	445.319	0	229.826	0	0	0	0	0	0	0	83.2203	41.9754	5.414724
Glutamate--cysteine ligase (EC 6.3.2.2), divergent, of Alpha- and Beta-proteobacteria type	0	12.196	5.93566	0	0	0	0	0	0	0	0	0	1.192844
Glutamate-1-semialdehyde aminotransferase (EC 5.4.3.8)	6.36223	7.97158	10.4518	0	0	0	0	0	0	0	0	0	0
Glutamate-aspartate carrier protein	0.221306	0	0.116066	0	0	0	0	0	0	0	0	0	0
Glutamine synthetase type I (EC 6.3.1.2)	9.6878	0	6.45739	0	0	0	0	0	0	6.24512	0	3.20604	1.358951
Glutamine-dependent 2-keto-4-methylthiobutyrate transaminase	0	0	0	0	0	0	77.7862	0	38.1923	0	0	0	0
Glutamyl-tRNA reductase (EC 1.2.1.70)	0	0	0	3428.99	0	1992.52	0.345755	0	0.169762	0	0	0	69.744852
Glutamyl-tRNA synthetase (EC 6.1.1.17)	0	0	0	0	0	0	0	56.0609	28.5356	167.951	0	88.5251	0
Glutamyl-tRNA(Gln) amidotransferase asparaginase subunit (EC 6.3.5.7)	0	54.7857	26.0407	0	0	0	0	3.93809	2.00452	0	0	0	0
Glutamyl-tRNA(Gln) synthetase (EC 6.1.1.24)	0	0	0	0	0	0	0	0	0	13.9527	0	7.16286	0
Glutathione reductase (EC 1.8.1.7)	10.1226	0	5.3089	0	0	0	0	0	0	0	0	0	0.985397
Glutathione S-transferase family protein	399.89167	0	211.47841	0	0	0	0	0	0	0	0	0	0.75745
Glutathione synthase/Ribosomal protein S6 modification enzyme (glutaminyl transferase)-like	0	0	0	0	0	0	0	14.4913	7.37619	0	0	0	0
Glutathione synthetase (EC 6.3.2.3)	47.5232	0	24.9658	0	0	0	0	0	0	0	0	0	1.051857
Glycerate kinase (EC 2.7.1.31)	0	0	0	2.99629	0	1.5618	0	0	0	0	0	0	0
Glycerol kinase (EC 2.7.1.30)	0	280.8472	383.9734	0	0	0	605.64656	0	297.24811	0	0	0	0.47432
Glycerol-1-phosphate dehydrogenase (EC 1.1.1.261)	0	0	0	0	0	0	0	29.3015	15.376	0	0	0	0
Glycerol-3-phosphate ABC transporter, permease protein UgpA (TC 3.A.1.1.3)	155.0456	0	102.7277	212.918	0	126.5161	0	0	0	0	0	0	2.443186
Glycerol-3-phosphate acyltransferase (EC 2.3.1.15)	0.179701	0	0.0942455	0	0	0	0	0	0	0	0	0	0
Glycerol-3-phosphate regulon repressor, DeoR family	0	0	0	1.92446	0	1.11885	0	0	0	0	0	0	0.146267
Glycine cleavage system H protein	0	1302.22	622.969	0	0	0	0	0	0	0	0	0	0.579295
Glycine cleavage system transcriptional activator	41.4885	787.76	513.947	0	0	0	0	268.4	140.664	0	0	0	1.856575
Glycine dehydrogenase (glycine cleavage system P1 protein) (EC 1.4.4.2)	0.327125	0	0.171563	0	0	0	0	125.57	62.9237	0	0	0	0
Glycine dehydrogenase (glycine cleavage system P2 protein) (EC 1.4.4.2)	0	1507.55	767.472	0	0	0	0	0	0	0	0	0	0
Glycine-rich cell wall structural protein precursor	0	0	0	0	0	0	0	0	360.808	0	0	0	0
Glycine/serine hydroxymethyltransferase	0	43.2297	20.5575	0	0	0	0	0	0	0	0	0	0
Glycogen phosphorylase (EC 2.4.1.1)	0	0	0	0	0	0	0	0	0	0	432.48	212.568	0
Glycogen synthase, ADP-glucose transglucosylase (EC 2.4.1.21)	0	296.04	141.595	0	0	0	0	0	0	0	0	0	0
Glycoside hydrolase, family 9	0	28.7	14.0992	0	0	0	0	0	0	0	0	0	0
Glycosyl hydrolase, BNR repeat precursor	0	0	0	0	0	0	0	69.4517	35.3515	0	0	0	0
Glycosyl transferase family 2	0	0	0	0	0	0	0	0.972153	0.494835	0	0	0	0
Glycosyl transferase, family 9	0	0	0	18.8081	0	11.0098	0	0	0	0	0	0	0
Glycosyltransferase (EC 2.4.1.-)	0	0.77115	0	0	0	0	0	0	0	0	0	0	0
Glycyl-tRNA synthetase beta chain (EC 6.1.1.14)	0	0	0	56.9797	0	33.3406	0	0	0	0	0	0	1.067493
GTP cyclohydrolase I (EC 3.5.4.16) type 1	10.01895	0	5.26412	0	0	0	0	0	0	0	0	0	0
GTP-binding protein EngA	817.053	0	445.327	0	0	0	0	0	0	0	0	0	0.275855
GTP-binding protein related to HflX	0	0	0	86.3526	0	53.4564	0	0	0	0	0	0	0
Guanosine-5'-triphosphate,3'-diphosphate pyrophosphatase (EC 3.6.1.40)	0	0	0	0	0	0	111.8305	0	54.5669	0	0	0	0
H+ Antiporter protein	0	0	22.3384	0	0	0	0	0	0	0	0	0	0
Haloalkane dehalogenase 2 (EC 3.8.1.5)	0	0	0	0	0	0	0	0	0	0	0.277722	0.135148	0
Heat shock hsp20 (Alpha crystallin) proteins family	0	0	0	0	0	0	0	88.7325	45.1657	0	0	0	0
Heat shock protein 60 family chaperone GroEL	0	35.50343	17.34317	9.08507	0	5.42279	575.78	704.89502	682.03502	0	0	0	1.996728
Heat shock protein X	0	0	0	0	0	0	9.95793	0	4.88925	0	0	0	0
Heat-inducible transcription repressor HrcA	2.77697	0	1.4564	0	0	0	0	0	0	0	9.10598	4.4138	0.518364
Helicase (Snf2/Rad54 family)	0	1.93186	0.918678	0	0	0	0	0	0	0	0	0	0
Helicase loader DnaI	0	0	0	0	0	0	0	0	0	3.771	0	1.93591	0
Helix-turn-helix domain protein	0.224626	0	0.117807	0	0	0	0	0	0	0	0	0	0
Heme ABC transporter, ATPase component HmuV	0	0	0	0	0	0	0	0.994119	0.506016	0	0	0	0
Heme d1 biosynthesis protein NirG	0	273.264	128.3899	0	0	0	0	0	0	0	0	0	0
Hemolysin-type calcium-binding region	0	0	0	0	0	0.109973	0	0	0	0	0	0	0
Hemolysins and related proteins containing CBS domains	0.406563	0	0.213225	0	0	0	0	646.607	354.063	0	0	0	0
Heterodisulfide reductase, iron-sulfur binding subunit, putative	0	59.9512	29.2804	0	0	0	0	0	0	0	0	0	0
Heterodisulfide reductase, subunit A/methylviologen reducing hydrogenase, subunit delta	0	2123.96	1071.99	0	0	0	308.015	12.9251	201.92863	0	0	0	0
HflC protein	0	261.168481	119.339673	0	0	0	44.8578	34.2808	39.9978	0	0	0	0.428819
HflK protein	19.606776	71.54625	44.053576	0	0	0	0	6.51115	3.31424	0	0	0	1.005504
Histidine ammonia-lyase (EC 4.3.1.3)	0	137.739	65.5006	0	0	0	0	124.8289	63.5391	3.48968	0	1.805382	0.35255

Histidine kinase of the competence regulon ComD	0	0	0	0	0	0	0	0	0	730.711	0	376.778	0
Histidinol-phosphate aminotransferase (EC 2.6.1.9)	1114.2646	0	599.344807	0	0	0	0	3.56015	1.812146	0	0	0	0.689951
Histidyl-tRNA synthetase (EC 6.1.1.21)	0	0	0	656.398	0	401.484	0	0	0	0	0	0	1.389317
Histone acetyltransferase HPA2 and related acetyltransferases	0	0	0	0	64.4946	27.2182	0	0	0	0	0	0	5.423561
Hnr protein	0	0	0	2.56391	0	1.49063	0	0	0	0	0	0	0
HPr kinase/phosphorylase (EC 2.7.1.-) (EC 2.7.4.-)	11886.37	0	6267.86	0	0	0	0	0	0	0	14.7192	0	0.83338
HTH DNA-binding protein	0	57.7926	30.0045	0	0	0	0	0	0	0	0	0	0
HtrA protease/chaperone protein	0	0	0	16.5058	0	9.59625	3.2165	0	1.525594	0	0	0	2.830939
Hydantoin racemase (EC 5.1.99.-)	0	0	0	0	0	0	382.525	0	187.216	0	0	0	0
Hydrogenase metallocenter assembly protein HypF	0	0	0	0	0	0	3.29028	0	1.615495	0	0	0	0
Hydrolase (HAD superfamily)	0	0	0	0	0	0	0	29.2994	14.91367	0	0	0	0
Hydrolase, alpha/beta fold family	476.625	0	249.9408	0	0	0	0	0	0	0	0	0	0.198032
Hydrolase, CocE/NonD family	0	0	0	0	0	0	0	20.8388	11.6451	0	0	0	0
Hydrolase, HAD subfamily IIIA	0	0	0	0	0	0	0	0	0	3.81864	0	2.03576	0
Hydrolase, UxaA family	0	0	0	0	0	0	0	118.4272	60.6026	0	0	0	0
Hydroxybenzoyl-CoA reductase subunit # FIG predicted	0	0	0	0	0	0	0	0	0	2.92957	0	0	0
Hydroxylamine oxidoreductase (Fragment)	0	0	0	0	0	0	0	85.3209	42.749	0	0	0	0
Hydroxylamine reductase (EC 1.7.-.-)	0	0	0	0	0	0	0	0	0	0.475661	0	0.244189	0
Hydroxymethylglutaryl-CoA lyase (EC 4.1.3.4)	275.0808	0	143.7304	0	0	0	0	0	0	0	0	0	0.458679
Hydroxymethylglutaryl-CoA reductase (EC 1.1.1.34)	0	0	0	0	0	0	0	0	0	1911.548	0	2153.06	0
Hydroxymethylpyrimidine ABC transporter, transmembrane component	0	0	0	118.407	0	65.8723	0	0	0	0	0	0	0
Hydroxymethylpyrimidine phosphate synthase ThiC (EC 4.1.99.17)	0	41.6809	20.5222	0	0	0	0	0	0	0	0	0	0.275855
Hypothetical ATP-binding protein UPF0042, contains P-loop	199.985	0	104.649	0	0	0	0	0	0	0	0	0	0.167052
Hypothetical lipoprotein	0	0	0	0	0	0	0	3.87313	1.971458	0	0	0	0
Hypothetical lipoprotein yghG precursor	0.359401	0	0.188491	0	0	0	0	0	0	0	0	0	0
Hypothetical membrane spanning protein	0	0	0	0	0	0	1.9499	0	0.957384	0	0	0	0
hypothetical mobile element-associated protein	0	0	0	0	0	0	0	0	0	1.72357	0	0.884824	0
Hypothetical nudix hydrolase YeaB	0	27.9233	13.2787	0	0	0	0	0	0	0	0	0	0.667184
Hypothetical oxidoreductase YqhD (EC 1.1.-.-)	0	1.8007	0.856306	0	0	0	0	0	0	0	0	0	0
Hypothetical phage protein	0	0	0	82.0145	0	47.6821	0	0	0	0	0	0	0
Hypothetical protein SAV2252	0	0	0	0	0	0	0	31.9994	16.288	0	0	0	0
hypothetical protein sometimes fused to ribosomal protein S6 glutaminyl transferase / Ribosomal protein S(	0	0	0	0	0	0	0	0.302573	0.154013	0	0	0	0
Hypothetical protein UPF0052	271.941	0	142.662	0	0	0	0	0	0	0	0	0	0
Hypothetical protein YbgA	0	0	0	0	0	0	0	322.815	165.838	0	0	0	0
Hypothetical protein YbgI	277.254	0	145.6242	0	0	0	0	0	0	0	0	0	1.009579
Hypothetical protein-transmembrane prediction	0	0	0	0	0	0	0	0	0	422.148	0	215.544	0
Hypothetical protein-transmembrane region and signal peptide prediction	0	0	0	0	0	0	0	16.0705	8.18004	0	0	0	0
Hypothetical protein, putative ABC transporter associated permease	0	0	0	0	0	0	0	30.0345	15.2614	0	0	0	0
Hypothetical protein, putative adhesion or S-layer protein	0	0	0	0	0	0	0	818.58	413.706	0	0	0	0
Hypothetical protein; FIG000859	0	0	0	0	0	0	0	14.7718	7.67403	0	0	0	0
Hypothetical protein; FIG002842	0	0	0	23.6368	0	13.7421	0	0	0	0	0	0	0.328786
Hypothetical protein; FIG003307	0	0	0	0	0	0	0	736.028	376.007	0	0	0	0
Hypothetical protein; FIG00347935	0	11.4338	5.5667	0	0	0	0	0	0	0	0	0	0
Hypothetical protein; FIG00348156	0	0	1046.75	0	0	0	0	0	0	0	0	0	0
Hypothetical protein; FIG00350121	16.2812	0	8.5388	0	0	0	0	0	0	0	0	0	0
Hypothetical protein; FIG00350294	118.431	0	42.7471	0	0	0	0	0	0	0	0	0	0
Hypothetical protein; FIG00350342	0	281.313	133.7759	0	0	0	0	0	0	0	0	0	0
Hypothetical protein; FIG00351164	2.36448	0	1.24007	0	0	0	0	0	0	0	0	0	0
Hypothetical protein; FIG00351471	0	0	0.841478	0	0	0	0	0	0	0	0	0	0
Hypothetical protein; FIG00431079	0	0	0	0	0	0	0	46.1118	24.1315	0	0	0	0
Hypothetical protein; FIG00441227	1388.885	0	333.121	0	0	0	0	0	0	0	0	0	0.141655
Hypothetical protein; FIG00452163	0	0	0	0	2980.56	1288.98	0	0	0	0	0	0	0
Hypothetical protein; FIG00452747	0	0	0	0	0	0	0	0	0	79.8705	0	41.0633	0
Hypothetical protein; FIG00465763	0	0	0	0	0	0	0	0	0	0	972.139	473.051	0
Hypothetical protein; FIG00481472	0	0	0	0	0	0	0	0	0	0	13.28968	6.46719	0
Hypothetical protein; FIG00512995	0	0	0	0	0	0	0	0.203049	0	0	0	0	0
Hypothetical protein; FIG00520126	1.03873	0	0.544772	0	0	0	0	0	0	0	0	0	0
Hypothetical protein; FIG00521049	0	511.873	229.687	0	0	0	0	0	0	0	0	0	0
Hypothetical protein; FIG00530012	0	5.2709	2.50653	0	0	0	0	0	0	0	0	0	0
Hypothetical protein; FIG00536986	87.9139	0	46.1362	0	0	0	0	0	0	0	0	0	0
Hypothetical protein; FIG00537682	17.99187	0	9.43599	0	0	0	0	0	0	0	0	0	0
Hypothetical protein; FIG005429	0.165166	0	0.0866227	0	0	0	0	0	0	0	0	0	0
Hypothetical protein; FIG00544776	2.13668	0	1.0344	0	0	0	0	0	0	0	0	0	0
Hypothetical protein; FIG00545606	0	0	0	0	0	0	0	0	0	0.214657	0	0.110198	0
Hypothetical protein; FIG00547247	0	0	0	0	0	0	0	0	0	6.4089	0	3.29012	0
Hypothetical protein; FIG00577025	0	0	0	0	0	0	0	17.0331	8.67001	0	0	0	0
Hypothetical protein; FIG00577033	0	0	0	0	0	0	0	40.3166	11.8153	0	0	0	0
Hypothetical protein; FIG00577375	0	46.3151	23.4333	0	0	0	0	0	0	0	0	0	0
Hypothetical protein; FIG00581088	0	0	0	0	0	0	0	1.87073	0.952221	0	0	0	0
Hypothetical protein; FIG00581358	0	0	0	0	0	0	0	0	0	0	0.221342	0.107712	0
Hypothetical protein; FIG00581518	0	0	0	0	0	0	146.6979	0	71.6316	0	0	0	0
Hypothetical protein; FIG00581832	0	2.6903	1.57254	0	0	0	0	0	0	0	0	0	0
Hypothetical protein; FIG00582348	0	0	0	0	0	0	0	1106.016	567.491	0	0	0	0
Hypothetical protein; FIG00582897	4.80148	0	2.58387	0	0	0	0	0	0	0	0	0	0
Hypothetical protein; FIG00586707	0	0	0	0	0	0	0	0	0	0	0	2.07954	0
Hypothetical protein; FIG00607773	0	0	0	0	0	0	0	4.25662	2.166661	0	0	0	0
Hypothetical protein; FIG00627631	0	0	0	0	0	0	0	0	0	0	1020.103	490.226	0
Hypothetical protein; FIG00637996	0	0	0	0	0	0	0	0	0	0	0.262844	0.127908	0
Hypothetical protein; FIG00638088	0	0	0	0	0.46998	0.19674	0	0	0	0	0	0	0
Hypothetical protein; FIG00638504	0	0	0	492.549	0	284.167	0	0	0	0	0	0	0
Hypothetical protein; FIG00640476	0	0	0	0	0	0	24.747	0	12.1506	0	0	0	0
Hypothetical protein; FIG00640568	0.208954	0	0.109588	0	0	0	0	0	0	0	0	0	0
Hypothetical protein; FIG00646873	0	0	0	45.3812	0	84.5355	0	0	0	0	0	0	0
Hypothetical protein; FIG00648199	0	0	0	0	0	0	0	0.208211	0.105982	0	0	0	0
Hypothetical protein; FIG00648345	0	0	0	0	0	0	18.7009	0	9.18198	0	0	0	0
Hypothetical protein; FIG00653436	0	0	0	0	0	0	0	3.24661	1.65256	0	0	0	0
Hypothetical protein; FIG00659688	0	0	0	0	0	0	4.02909	0	4.02566	0	0	0	0
Hypothetical protein; FIG00663041	0	0	0	0	0	0	0	138.364	70.4471	0	0	0	0
Hypothetical protein; FIG00680164	0	0	0	0	0	0	0	33.5741	15.3408	0	0	0	0
Hypothetical protein; FIG00688519	0	0	0	0	0	0	0	11.5897	5.89928	0	0	0	0
Hypothetical protein; FIG00693527	0	0	0	0	0	0	0	0	0	25.5734	0	13.1082	0
Hypothetical protein; FIG00729359	0	0	0	0	0	0	0	0	0	153.3273	0	0	0
Hypothetical protein; FIG00731616	0	0	0	763.473	0	443.849	0	0	0	0	0	0	0
Hypothetical protein; FIG00753443	0.214953	0	0.112734	0	0	0	0	0	0	0	0	0	0
Hypothetical protein; FIG00758552	0	0	0	0.437909	0	0.254594	0	0	0	0	0	0	0
Hypothetical protein; FIG00759958	0	0	0	0	0	0	0	16.3998	7.59729	0	0	0	0
Hypothetical protein; FIG00764857	0	161.797	82.131	0	0	0	0	0	0	0	0	0	0
Hypothetical protein; FIG00772051	0	0	0	0	0	0	0	11.09944	6.35674	0	0	0	0
Hypothetical protein; FIG00772742	0	0	0	0	0	0	0	0.238534	0.121416	0	0	0	0
Hypothetical protein; FIG00772929	0	0	0	0	0	0	0	18.67302	9.49582	0	0	0	0
Hypothetical protein; FIG00775401	32.942	0	17.7203	0	0	0	0	0	0	0	0	0	0
Hypothetical protein; FIG00781076	0	0	0	0	0	0	0	0	0	57.1881	0	29.0511	0
Hypothetical protein; FIG00784450	0.18115	0	0.0950056	0	0	0	0	0	0	0	0	0	0
Hypothetical protein; FIG00795790	0	4.78685	2.27635	0	0	0	0	0	0	0	0	0	0
Hypothetical protein; FIG00796651	0	0	0	0	0	0	16.8082	0	9.2376	0	0	0	0
Hypothetical protein; FIG00809930	0	0	0	0	0	0	0	0	0	0	0.255987	0.124572	0
Hypothetical protein; FIG00818530	0	0	0	0	0	0	0	0	0	91.9087	0	47.3801	0
Hypothetical protein; FIG00818702	0	0	0	0	0	0	0	0	0	7.24728	0	3.72052	0
Hypothetical protein; FIG00818801	0	0	0	0	0	0	0	0	0	23.6389	0	12.13543	0
Hypothetical protein; FIG00820860	0	0	0	0	303.449	55.3273	0	0	0	0	0	0	0
Hypothetical protein; FIG00823043	0	0	0	0	0	0	0	88.1763	44.6009	0	0	0	0
Hypothetical protein; FIG00824483	0	0	0	0	0	0	0	0	0	0	691.43	338.423	0
Hypothetical protein; FIG00825507	0	0	0	94.4839	0	55.0522	0	0	0	0	0	0	0
Hypothetical protein; FIG00828859	0	0	0	0	364.818	158.5283	0	0	0	0	0	0	0
Hypothetical protein; FIG00839627	0	0	0	0	0	0	0	12.5048	6.36508	0	0	0	0
Hypothetical protein; FIG00841068	0	95.6748	702.564	0	0	0	0	0	0	0	0	0	0
Hypothetical protein; FIG00841528	0	0	0	0	0	0	0	4.05448	2.06377	0	0	0	0
Hypothetical protein; FIG00853390	0.206079	0	0.10808	0	0	0	0	0	0	0	0	0	0
Hypothetical protein; FIG00855974	0	0	0	0	0	0	0	2.08211	1.05982	0	0	0	0
Hypothetical protein; FIG00866958	0	0	0	0	0	0	0	0	0	0	0.252691	0.122968	0
Hypothetical protein; FIG00877732	0	0	40.7631	0	0	0	0	0	0	0	0	0	0
Hypothetical protein; FIG00883824	6.43738	0	3.37614	0	0	0	0	0	0	0	0	0	0
Hypothetical protein; FIG00897017	0	0	0	0	0	0	0	0	0	6.51939	0	3.34684	0
Hypothetical protein; FIG00898333	0	0	0	0	0	0	0	135.408	69.72	0	0	0	0
Hypothetical protein; FIG00908315	0.211911	0	0.111139	0	0	0	0	0	0	0	0	0	0
Hypothetical protein; FIG00908880	0	0	0	0	0	0	0	0.816102	0.415403	0	0	0	0
Hypothetical protein; FIG00911243	0	0	0	0	0	0	0	0	0	277.779	0	142.857	0
Hypothetical protein; FIG00933687	0	0	0	0	0	0	0	83.9924	40.2	0	0	0	0
Hypothetical protein; FIG00937153	0	0	0	38.2803	0	22.4518	0	0	0	0	0	0	0

Hypothetical protein; FIG00937668	0	0	0	0	0	0	10.9406	0	5.37171	0	0	0	0
Hypothetical protein; FIG00937904	0	0	0	0	0	0	0	0	0	0.267038	0	0.137088	0
Hypothetical protein; FIG00938297	0	0	0.219516	0	0	0	0	0	0	0	0	0	0
Hypothetical protein; FIG00944965	0	0	0	0	0	0	21.9626	0	11.00812	0	0	0	0
Hypothetical protein; FIG00945103	0	0	0	0	0	0	0	0	0	23.2267	0	18.5291	0
Hypothetical protein; FIG00945414	0.18115	0	0.0950056	0	0	0	0	0	0	0	0	0	0
Hypothetical protein; FIG00945431	0.187971	0	0.0985832	0	0	0	0	0	0	0	0	0	0
Hypothetical protein; FIG00945491	0	0	0	0	0	0	0	0	0	0	0	0.14114	0
Hypothetical protein; FIG00945575	0	0	0	0.890714	0	0.550097	0	0	0	0	0	0	0
Hypothetical protein; FIG00945969	0	0	0	0	0	0	0	0.939401	0.478164	0	0	0	0
Hypothetical protein; FIG00946062	0	0	0	0	0	0	4.513	0	2.64079	0	0	0	0
Hypothetical protein; FIG00946150	0.195327	0	0.102441	808.069	0	470.161	0	0	0	0	0	0	0
Hypothetical protein; FIG00946322	42.5736	0	22.4385	0	0	0	0	0	0	0	0	0	0
Hypothetical protein; FIG00946491	0	0	0	0	0	0	0	0	8.7685	0	0	0	0
Hypothetical protein; FIG00947995	0	0	0	25.1629	0	14.6686	0	0	0	0	0	0	0
Hypothetical protein; FIG00953333	0	0	0	0	0.44546	0.186475	0	0	0	0	0.270078	0.131429	0
Hypothetical protein; FIG00953536	0.200559	0	0.105185	0	0	0	0	0	0	0	0	0	0
Hypothetical protein; FIG00955429	0.187971	0	0.0985832	0	0	0	0	0	0	0	0	0	0
Hypothetical protein; FIG00955932	0	0	0	0	0	0	0	80.8774	41.0259	0	0	0	0
Hypothetical protein; FIG00965493	0	0	0	0	0	0	0	0	0	0	0.224721	0.109357	0
Hypothetical protein; FIG00967478	0.816821	0	0.428389	0	0	0	0	0	0	0	0	0	0
Hypothetical protein; FIG00970189	6372.66	0	3403.34	0	0	0	0	0	0	0	0	0	0.958764
Hypothetical protein; FIG00972990	0	0	0	0	0	0	0	801.732	428.916	0	0	0	0
Hypothetical protein; FIG00974280	0	0	0	0	836.85	348.866	0	0	0	0	0	0	0.987987
Hypothetical protein; FIG00974943	0	0	0	0	0	0	0	0	0	0.869574	0	0.446411	0
Hypothetical protein; FIG00975217	0	0	0	0	0	0	227.69	0	110.275	0	0	0	0
Hypothetical protein; FIG00975518	0	0	0	0	0	0	0	0	0	0	977.158	474.591	0
Hypothetical protein; FIG00975603	31.1413	0	0	0	0	0	0	0	0	0	0	0	0
Hypothetical protein; FIG00986611	0	0	0	0	0	0	0	0	0	2.06257	0	1.05886	0
Hypothetical protein; FIG00990916	0.208954	0	0.109588	0	0	0	0	0	0	0	0	0	0
Hypothetical protein; FIG01015257	0	0	0	202.223	0	120.069	0	0	0	0	0	0	0
Hypothetical protein; FIG01019539	0	0	0	0	0	0	0	0	0	21.6105	0	14.0925	0
Hypothetical protein; FIG01019723	0	0	0	0	0	0	0	4.59446	2.33862	0	0	0	0
Hypothetical protein; FIG01020158	0	0	0	0	0	0	0	16.9038	8.6559	0	0	0	0
Hypothetical protein; FIG01023765	0.126669	0	0.0664325	0	0	0	0	0	0	0	0	0	0
Hypothetical protein; FIG01035833	0	0	0	0	0	0	0	0	424.529	0	0	0	0
Hypothetical protein; FIG01041357	0	0	0	0	0	0	0	2.4569	1.25058	0	0	0	0
Hypothetical protein; FIG01055645	0	0	0	0	0	0	0	0	0	0	0	1.92959	0
Hypothetical protein; FIG01057559	0	0	0	0	0	0	0	13.3005	6.95813	0	0	0	0
Hypothetical protein; FIG01059430	0	0	0	0	0.304928	0.127647	0	0	0	0	0	0	0
Hypothetical protein; FIG01066692	0	0	0	2411.84	0	959.009	0	0	0	0	0	0	0
Hypothetical protein; FIG01081110	0	0	0	0	0	0	0	0.677097	0.344649	0	0	0	0
Hypothetical protein; FIG01082957	0	1345.546	640.064	0	0	0	0	0	0	0	0	0	0
Hypothetical protein; FIG01100088	0	393.393	193.414	0	0	0	0	0	0	0	0	0	0
Hypothetical protein; FIG01107839	0	0	0	0	0	0	0	0	0	685.833	0	344.733	0
Hypothetical protein; FIG01107902	912.702	0	478.983	0	0	0	0	0	0	0	0	0	0
Hypothetical protein; FIG01107905	0	0	0	0	0	0	0	0	0	0.882773	0	0.453187	0
Hypothetical protein; FIG01108163	971.285	0	503.598	0	0	0	0	0	0	0	0	0	0
Hypothetical protein; FIG01108661	0	0	0	0	0	0	0	0	0	39.9889	0	20.529	0
Hypothetical protein; FIG01108691	0	0	0	0	0	0	0	0	0	29.1365	0	20.3012	0
Hypothetical protein; FIG01111044	0	0	0	0	0	0	0	0	0	156.3011	0	93.7522	0
Hypothetical protein; FIG01111551	0	0	0	0	2.77376	1.12485	0	0	0	0	0	0	0
Hypothetical protein; FIG01111578	0.18115	0	0.0950056	0	0	0	0	0	0	0	0	0	0
Hypothetical protein; FIG01112244	0	0	0	0	2.17701	0.911328	0	0	0	0	0	0	0
Hypothetical protein; FIG01113078	0	0	0	291.453	0	167.3079	0	0	0	0	0	0	0
Hypothetical protein; FIG01114020	0	0	0	0	0	0	24.8593	0	13.5063	0	0	0	0
Hypothetical protein; FIG01119252	0	0	0	0	0	0	0	0	0	5.72561	0	1.54101	0
Hypothetical protein; FIG01145159	0	19.2822	10.6803	0	0	0	0	0	0	0	0	0	0
Hypothetical protein; FIG01161427	0	0	0	0	0	0	0	3.52664	1.61559	0	0	0	0
Hypothetical protein; FIG01164557	0	0	0	0	0	0	0	0	0	49.1091	0	25.9724	0
Hypothetical protein; FIG01179327	0	1.90563	0.906207	0	0	0	0	0	0	0	0	0	0
Hypothetical protein; FIG01186781	0	0	0	98.8701	0	57.2996	0	0	0	0	0	0	0
Hypothetical protein; FIG01196959	0	0	0	0	0	0	0	0.3578	0.182124	0	0	0	0
Hypothetical protein; FIG01197580	0	0	0	0	0	0	0	0.457401	0.166301	0	0	0	0
Hypothetical protein; FIG01198452	0	0	0	0	0	0	0	0	0	0	0.822304	0.40016	0
Hypothetical protein; FIG01239737	0	0	0	0	0	0	0	1.1812	0.601242	0	0	0	0
Hypothetical protein; FIG01287761	0	482.621	229.579	0	0	0	0	0	0	0	0	0	0
Hypothetical protein; FIG01289284	0	0	0	0	0	0	0	16.7417	8.52167	0	0	0	0
Hypothetical protein; FIG032766	0	0	0	0	0	0	0	0	0	0	21.9533	10.6832	0
Hypothetical protein; FIG062788	0	0	0	0	0	0	0	1.567237	0.791624	0	0	0	0
Hypothetical protein; FIG110192	0	0	0	0	21.59509	18.06133	0	0	0	0	0	0	0.704684
Hypothetical protein; putative membrane protein	0	183.3517	85.2675	0	0	0	0	0	0	0	0	0	0
Hypothetical sugar ABC transporter TM0460, substrate-binding protein	254.452	233	112.231	0	0	0	0	0	0	0	0	0	0
Hypothetical sugar ABC transporter TM1150, substrate-binding protein	0	88.6017	30.982	0	0	0	0	29.1399	15.2687	0	0	0	0
Hypothetical YciO protein, TsaC/YrdC paralog	0	58.0807	25.7033	0	0	0	0	0	0	0	0	0	0.22788
Hypothetical, related to broad specificity phosphatases COG0406	0.29556	0	0.155009	0	0	0	0	1.4	0.712612	0	0	0	0
Ig domain protein, group 1 domain protein	0	1.31415	0.624935	0	0	0	0	0	0	0	0	0	0
IgaA: a membrane protein that prevents overactivation of the Rcs regulatory system	0	0	0	192.8953	0	113.174	0	0	0	0	0	0	0
immunity protein, probable	0	0	0	0	0	0	0	3.0926	1.35553	0	0	0	0
Immunodominant staphylococcal antigen A precursor	0	0	0	0	0	0	0	0	0	337.4222	0	221.4136	0
IncF plasmid conjugative transfer regulator TraY	0	0	0	0	0	0	0	0	0	105.914	1261.86	69.9565	0
IncN plasmid KikA protein	0	0	0	197.025	0	114.422	0	0	0	0	0	0	0
IncP-type conjugative transfer protein TrbB	0	21.9077	10.9015	0	0	0	0	0	0	0	0	0	0
Indolepyruvate oxidoreductase subunit IorA (EC 1.2.7.8)	0	0	0	0	0	0	0	32.4903	16.53789	0	0	0	0
Inhibitor of vertebrate lysozyme precursor	0	0	0	10.6595	0	6.16452	0	0	0	0	0	0	0
Inner membrane component of tripartite multidrug resistance system	0	44.9167	22.8854	0	0	0	0	0	0	0	0	0	1.71956
Inner membrane metabolite transport protein YhjE	0	0	0	0	275.159	115.3221	0	0	0	0	0	0	0
Inner membrane protein CreD	0	0	0	0	0	0	2.39517	0	1.17601	0	0	0	0
Inner membrane protein YccF	0	0	0	0	0	0	0.207923	0	0.102088	0	0	0	0
Inner membrane protein YeaI	0.211911	0	0.111139	0	0	0	0	0	0	0	0	0	0
Inosine-5-monophosphate dehydrogenase	0	2122.02	1022.99	0	0	0	0	0	0	0	0	0	0
Inosine-uridine preferring nucleoside hydrolase (EC 3.2.2.1)	31.7588	0	16.6561	0	0	0	0	0	0	0	0	0	0.192339
Inosine/xanthosine triphosphatase (EC 3.6.1.-)	0	0	0	11.17427	0	8.1707	0	0	0	0	0	0	0
Inositol-1-phosphate synthase (EC 5.5.1.4)	0	0	0	0	0	0	0	1285.48138	659.67186	0	0	0	0
Inosose isomerase (EC 5.3.99.-)	0	0	0	0	0	0	0	59.4626	30.267	0	0	0	0
Integral membrane protein	0	0	0	0	0	0	0	0	0	54.1341	0	28.0981	0
integral membrane protein MviN	1931.704	0	1031.062	0	0	0	0	95.0429	49.6226	0	0	0	0
Integral membrane protein YfiB	0.197908	0	0	0	0	0	0	0	0	0	0	0	0
Integral membrane protein YggT, involved in response to extracytoplasmic stress (osmotic shock)	0	0	0	0	0	0	0	243.632	131.859	0	0	0	0
Integrase/recombinase	0	0.755281	0.359167	0	0	0	0	0	0	0	0	0	0
Integron integrase IntIPac	0	0	0	0	0	0	0	200.911	102.719	0	0	0	0
Intracellular PHB depolymerase (EC 3.1.1.-)	4186.42	0	25371	0	0	0	0	0	0	0	0	0	0.816945
Ion transport 2 domain protein	0	0	0	0	0	0	0	213.046	109.88	0	0	0	0
Iron binding protein IscA for iron-sulfur cluster assembly	10.291	0	5.3972	0	0	0	0	0	0	0	0	0	0
iron-chelator utilization protein	0	0	0	265.917	0.34672	183.795142	0	0	0	0	0	0	0
Iron-sulfur cluster assembly protein SufB	0	0	0	0	1823.02	762.679	0	1.64577	0.837711	0	0	0	0
Iron-sulfur cluster assembly protein SufD	0	0	0	0	0	0	0	0	0	158.8738	0	81.5625	0
Iron-sulfur cluster assembly scaffold protein IscU	11.9038	0	6.24306	0	0	0	0	0	0	0	0	0	0.484739
Iron-sulfur cluster assembly scaffold protein IscU/NifU-like	5526.83	0	0	0	0	0	0	0	0	0	0	0	0
IS, phage, Tn; Transposon-related functions	0	0	0	0	0.449367	0.188111	0	0	0	0	0	0	0
Isochorismatase family protein	0	0	0	0	8.53797	3.52077	0	0	0	0	0	0	0
Isochorismate synthase (EC 5.4.4.2) siderophore	0	0	0	7.11124	0	4.44349	0	0	0	0	0	0	0
Isocitrate dehydrogenase (EC 1.1.1.41)	0	0	0	0	0	0	0	29.6837	15.0628	0	0	0	0
Isocitrate dehydrogenase (EC 1.1.1.42)	0	40.7392	19.418	0	70.6951	31.83768	0	34.4993	17.6912	0	0	0	1.599617
Isocitrate dehydrogenase (EC 1.1.1.42); Monomeric isocitrate dehydrogenase (EC 1.1.1.42)	0.694209	0	0.364084	0	0	0	0	0	0	0	0	0	0
Isocitrate dehydrogenase phosphatase (EC 2.7.11.5)/kinase (EC 3.1.3.-)	0	0	7.20846	0	0	0	0	0	0	0	0	0	0.739978
Isocitrate lyase (EC 4.1.3.1)	107.4227	0	56.5078	0	0	0	0	0	0	0	0	0	2.45292
Isoleucyl-tRNA synthetase (EC 6.1.1.5)	0.412915	1279.891266	551.560352	0	0	0	0	36.58472	18.72239	0	0	0	7.953644
Isopentenyl phosphate kinase	0	0	0	0	0	0	166.196	0	0	0	0	0	0
ISPpu14, transposase Orf2	0.118224	0	0.0620036	0	81.8036	0	0	0	0	0	0	0	0
ISSpo9, transposase	62.733	0	31.1763	0	0	0	0	0	0	0	0	0	0
Junctophilin 1	513.864	0	269.679	0	0	0	0	0	0	0	0	0	0
KEKE-like motif-containing transcription regulator (Rlr1)/suppressor of sin4	0.224626	0	0.117807	0	0	0	0	135.0119	69.3994	0	0	0	0
Ketoglutarate semialdehyde dehydrogenase (EC 1.2.1.26)	0	0	0	30.0619	0	0	0	0	0	0	0	0	1.276985
Ketol-acid reductoisomerase (EC 1.1.1.86)	0	25.6297	12.2507	0	0	0	0	386.447	198.075	0	0	0	1.14549
Kinase interacting family protein	0	0	0	0	0	0	184.599	0	63.7546	0	0	0	0
Kup system potassium uptake protein	1.42083	28.6216	14.373514	0.9068	0	0.527201	0	0	0	0	0	0	3.519817
L-2,4-diaminobutyrate decarboxylase (EC 4.1.1.-)	0	0	0	0	0	0	0	311.068	151.406	0	0	0	0
L-arginine:glycine amidinotransferase	0	0	0	0	0	0	0	0	0	0.279054	0	0.143257	0

L-fucose isomerase (EC 5.3.1.25)	0	0	0	0	0	0	0	12.4128	6.93861	0	0	0	0
L-fucose mutarotase, type 2	0	0	0	0	0	0	0	0.529503	0.700758	0	0	0	0
L-lactate dehydrogenase (EC 1.1.1.27)	0	0	0	0	0	0	0	0	0	40.0114	0	20.5406	0
L-lactate dehydrogenase (EC 1.1.2.3)	0	512.923	244.339	0	256.884	103.3453	0	0	0	0	0	0	0
L-proline glycine betaine ABC transport system permease protein ProV (TC 3.A.1.12.1)	0	0	0	0	0	0	57.9145	0	28.5075	0	0	0	0
L-proline glycine betaine ABC transport system permease protein ProW (TC 3.A.1.12.1)	0	0	0	0	0	0	0	0	0	252.823	0	129.869	0
L-seryl-tRNA(Sec) selenium transferase (EC 2.9.1.1)	0.206079	0	0.10808	0	0	0	0	0	0	0	0	0	0
L-threonine 3-O-phosphate decarboxylase (EC 4.1.1.81)	365.257	0	192.7315	0	0	0	0	0	0	0	0	0	0.264264
L-threonine 3-O-phosphate decarboxylase (EC 4.1.1.81) / Cobyrinic acid A,C-diamide synthase	0.412157	0	0.216159	0	0	0	0	0	0	0	0	0	0
Lactose permease	0.247385	0	0.129743	0	0	0	0	0	0	0	0	0	0
Lanthionine biosynthesis protein LanB	0	0	0	0	0	0	0	0	0	152.90088	0	78.49437	0
Lead, cadmium, zinc and mercury transporting ATPase (EC 3.6.3.3) (EC 3.6.3.5); Copper-translocating P-type	577.09536	1944.7	1579.567362	3.06477	0	1.78182	142.096	0	73.6906	0	0	0	2.614193
LemA protein	0	0	0	0	0	0	123.6203	0	54.174	0	0	0	0
Leucyl aminopeptidase	0	336.317	159.948	0	0	0	0	11.3017	5.65889	0	0	0	0
Lipopolysaccharide ABC transporter, ATP-binding protein LptB	0	0	0	0	0	0	0	0	0	0	0	0	0
Lipopolysaccharide cholinephosphotransferase LicD3 (EC 2.7.8.-)	0	0	0	0	0	0	0	0.279193	0.142112	0	0	0	0
Lipoprotein, putative	0	0	0	0	0	77654.1	0	0	0	0	0	0	1.686073
LmbE family protein	0	0	0	0	0	0	0	0	0	60.4906	0	31.0984	0
Low-specificity D-threonine aldolase	0	0	0	0	108.3187	45.0062	0	0	0	0	0	0	0.715692
LSU ribosomal protein L10p (P0)	0	0	0	0	6037.54	2048.07	0	0	0	0	0	0	0
LSU ribosomal protein L11e (L5p)	0	0	0	0	0	0	0	60.2769	33.8137	0	0	0	0
LSU ribosomal protein L12a (P1/P2)	0	68.0466	468.093	0	0	0	0	0	0	0	0	0	0
LSU ribosomal protein L14p (L23e)	0	0	0	0	0	0	0	366.443	187.521	201.127	0	103.862	0
LSU ribosomal protein L15e	0	0	0	0	0	0	0	25.3255	13.0743	0	0	0	0
LSU ribosomal protein L15p (L27Ae)	0	0	0	0	0	0	0	0	0	2.1363	0	1.09671	0
LSU ribosomal protein L16p (L10e)	0	263.77508	125.48276	0	0	0	0	0	0	93.1496	0	47.8568	0.53954
LSU ribosomal protein L17e (L22p)	0	311.4333	148.20293	0	0	0	0	7.60676	3.69389	0	0	0	0
LSU ribosomal protein L17p	0	0	0	0	0	0	0	0	0	46.0726	0	23.6522	0
LSU ribosomal protein L18p (L5e)	0	0	0	0	0	0	0	0	0	315.246	0	161.255	0
LSU ribosomal protein L20p	0	0	0	0	0	0	2.04246	0	1.00283	9.88091	0	5.07253	0
LSU ribosomal protein L21e	0	1335.84	636.461	0	0	0	0	0	0	0	0	0	0
LSU ribosomal protein L24p (L26e)	0	189.086	91.0965	0	0	0	0	114.607	59.1316	105.193	0	53.3181	0
LSU ribosomal protein L26e (L24p)	0	0	0	0	0	0	0	0	0	4.0718	0	2.09033	0
LSU ribosomal protein L30e	0	35.8147	17.0847	0	0	0	0	0	0	0	0	0	0
LSU ribosomal protein L31p	0	0	0	0	0	0	0	0.171811	0.0874534	0	0	0	0
LSU ribosomal protein L35e (L29p)	0	0	0	0	0	0	0	34.4867	17.5541	0	0	0	0
LSU ribosomal protein L36e	0	0	0	0	0	0	0	0	0	1.2809	0	0.657574	0
LSU ribosomal protein L36p	0	0	0	0	0	0	0	0	0	114.749	0	61.9341	0
LSU ribosomal protein L3e (L3p)	0	0	0	0	0	0	0	30.1795	13.4391	0	0	0	0
LSU ribosomal protein L6p (L9e)	0	169.7396	128.8363	0	0	0	0	15.0108	7.64064	0	0	0	0.241532
Lysine-arginine-ornithine-binding periplasmic protein precursor (TC 3.A.1.3.1)	0	19.0998	9.08274	0	0	0	0	0	0	0	0	0	0
Lysine-epsilon oxidase (EC 1.4.3.20) antimicrobial protein LodA	0	0	0	0	0	0	0	0	0	0	1.122654	0.54632	0
LysR family transcriptional regulator	0.264266	0	0.207895	0	206.51	68.6652	0	0	5.95312	0	0	0	1.67562
Lysyl endopeptidase (EC 3.4.21.50)	0	182.445	87.6218	0	0	0	0	0	0	0	0	0	0
Lysyl-tRNA synthetase (class I) (EC 6.1.1.6)	0	0	0	0	0	0	2353.71	0	1916.6	0	0	0	0
Lysyl-tRNA synthetase (class II) (EC 6.1.1.6)	0.200559	0	0.105185	0.850196	44.9154	19.295222	0	0.463565	0.235959	0	759.445	369.571	0.2396
Macrolide export ATP-binding/permease protein MacB (EC 3.6.3.-)	10567.4	16.5783	5543.64407	0	0	0	0	0	0	0	0	0	0
Major facilitator:Oxalate:Formate Antiporter	0.222402	0	0.116641	0	0	0	0	0	0	0	0	0	0
Malate Na(+) symporter	0	0	0	0	0	0	0	0.316338	0.161019	0	0	0	0
Malate:quinone oxidoreductase (EC 1.1.5.4)	0	0	0	0	0	0	0	0	0	250.253	0	129.5539	0
Malonyl CoA-acyl carrier protein transacylase (EC 2.3.1.39)	129.27717	0	58.6531689	0	0	0	0	0	0	0	0	0	1.293696
Maltodextrin phosphorylase (EC 2.4.1.1)	0	0	0	0	0	0	0	0	0	208.169	0	109.33	0
Maltose phosphorylase (EC 2.4.1.8) / Trehalose phosphorylase (EC 2.4.1.64)	0.313431	0	0.164382	0	0	0	0	0	0	0	0	0	0
Maltose/maltodextrin ABC transporter, permease protein MalG	0	0	0	9.40816	0	5.50927	0	0	0	0	0	0	0.179957
Maltose/maltodextrin ABC transporter, substrate binding periplasmic protein MalE	0	0	0	0	0	0	0	175.408	90.6673	0	0	0	0
Maltose/maltodextrin transport ATP-binding protein MalK (EC 3.6.3.19)	0	634.869	310.081	6.41229	0	3.74513	0	0	0	0	0	0	0.786916
Manganese superoxide dismutase (EC 1.15.1.1)	0	0	0	0	0	0	0	0.964484	0.490931	300.668	0	970.632	0
Manganese-dependent inorganic pyrophosphatase (EC 3.6.1.1)	0	0	0	0	0	0	0	0	0	81.688	0	42.1951	0
Mannitol-1-phosphate 5-dehydrogenase (EC 1.1.1.17)	0	16.57694	10.2671	0	0	0	0	0	0	0	0	0	0
Mannosyltransferase	3.35077	0	0	0	0	0	0	0	0	0	0	0	0
MCE-FAMILY PROTEIN MCE4B	0	0	0	0	0	0	0	0	0	0	3.23273	1.57315	0
Mediator of hyperadherence YidE	0	0	0	0	77.2725	32.3474	0	0	0	0	0	0	0
Membrane bound hydrogenase, MbhH subunit	0	0	0	0	0	0	120.052	0	58.9445	0	0	0	0
Membrane protein ydfJ	0	0	0	0	0	0	0	0	0	1.38462	0	3.55409	0
Membrane protein, suppressor for copper-sensitivity ScsD	0	0	0	0	0	0	0.323779	0	0	0	0	0	0
Membrane-associated phospholipid phosphatase	0	0	0	0	0	0	0	4.21581	2.14589	0	0	0	0
Membrane-bound lytic murein transglycosylase B precursor (EC 3.2.1.-)	0	0.829725	0.394569	0	0	0	0	76.4082	39.6127	0	0	0	0.161269
Metal-dependent protease	0	776.319	385.239	0	0	0	0	15.7067	7.82231	0	0	0	0
Metallo-beta-lactamase family protein, RNA-specific	0	599.74	285.546	0	0	0	0	0	0	0	0	0	0.415825
Metalloprotease, insulinase family	1330.159	0	658.489	0	0	0	0	0	0	0	0	0	0
Methanol:corrinoid methyltransferase	0	121.234	58.1856	0	0	0	0	0	0	0	0	0	0
Methionine ABC transporter ATP-binding protein	0	10.7208	5.11851	0	0.342661	0.143443	0	0	0	0	0	0	1.243278
Methionine biosynthesis and transport regulator MtaR, LysR family	0	0	0	0	0	0	18.1478	0	8.72282	0	0	0	0
Methionine gamma-lyase (EC 4.4.1.11)	0	296.238	141.368	0	0	0	0	0	0	0	0	0	17.337988
Methionyl-tRNA synthetase, clostridial paralog	0	0.222781	0.105941	0	0	0	0	0	0	0	0	0	0
Methyl-accepting chemotaxis protein	0.65425	0	0.343127	0	0	0	0	0	0	0	0	0	0
Methylated-DNA--protein-cysteine methyltransferase (EC 2.1.1.63)	180.389	0	96.5794	0	0	0	0	0	0	0	0	0	0.161269
Methylmalonyl-CoA mutase (EC 5.4.99.2)	0	0	0	0	0	0	7.02571	19.71192	3.31652	0	0	0	0
Methyltransferase	0	0	0	0	0	0	0	49.37018	25.18656	0	0	0	0
Methyltransferase FkbM	0	0	0	0	0	0	0	3.70652	1.88666	0	0	0	0
Methyltransferase TM1293	0	0	0	0	0	0	0	1.40703	0.550918	0	0	0	0
Mevalonate kinase (EC 2.7.1.36)	0	7.27366	3.46326	0	0	0	0	0.289046	0.147127	0	0	0	0
MFS permease protein	0	0	0	4.94341	0	2.87403	0	0	0	0	0	0	0.927154
Mg-chelatase subunit ChlD	0.529013	0	0.277445	0	0	0	0	0	0	0	0	0	0
Mg/Co/Ni transporter MgtE / CBS domain	0.463146	0	0.242901	0	0	0	0	0	0	0	0	0	0
Mg++ transporter	0	0	0	0	0	0	0	1.31619	0.669955	0	0	0	0
Microbial collagenase, secreted (EC 3.4.24.3)	0	230.6572	202.0411	0	0	0	0	0	0	0	0	0	0
MinD superfamily P-loop ATPase containing an inserted ferredoxin domain	0	0.253651	0.120621	0	0	0	1.36979	126.402	66.614952	0	0	0	0
Mll5128 protein	0	0	0	0	0	0	0	51.6809	28.2178	0	0	0	0
Mlr3099 protein	0	0	485.047	0	0	0	0	0	0	0	0	0	0
Mlr6856 protein	0	0	0	0	0	0	66.2179	0	32.7367	0	0	0	0
MlrC	0	3.1665	1.32865	0	0	0	0	0	0	0	0	0	0.154154
MmgE/PrpD family protein	0	0	0	0	0	0	0	1037.19	440.127	0	0	0	0
Molecular chaperone (small heat shock protein)	0	0	0	0	0	0	0	0	0	4.27124	0	12.6008	0
MORN repeat family protein	0	538.166561	265.0606163	0	0	0	0	0	0	0	0	0	0
MOSC domain protein	0	0	0	0	362.864	155.6893	0	0	0	0	0	0	0
MoxR-like ATPase in aerotolerance operon	0	0	0	0	0	0	0	172.242	87.4307	0	0	0	0
MSHA biogenesis protein MshM	0	0	0	0	0	0	0	88.5613	45.0262	0	0	0	0
Multi antimicrobial extrusion protein (Na(+)/drug antiporter), MATE family of MDR efflux pumps	0	0	0	0	0.265774	0.111256	0	0	0	0	1166.06	549.409	0
Multi-sensor hybrid histidine kinase	0	0	0	0	0	0	0	16.4423	8.36929	0	0	0	0
Multidrug efflux membrane fusion protein MexE	0	0	0	0	0	0	4.40117	0	2.16093	0	0	0	0
Multidrug resistance protein 1	0	0	0	12.93819	0	7.52208	0	0	0	0	0	0	0
Multidrug resistance protein B (ErmB)	0.136551	0	0.0716151	0	0	0.19674	0	0	0	0	0	0	0
Multidrug transporter MdtD	0	0	0	0	241.054	101.325	0	0	0	0	0	0	0
Multimodular transpeptidase-transglycosylase (EC 2.4.1.129) (EC 3.4.-.-) / Penicillin-binding protein 1A/1B (I	0	0	0	0	0	0	0	0	0	1214.199	0	737.909	0
Multiple antibiotic transporter	0	0	0	10.8719	0	6.91838	0	0	0	0	0	0	0.46815
Multiple sugar ABC transporter, ATP-binding protein	0	363.22	196.961	0	0	0	0	0	0	0	0	0	0
Muramoyltetrapeptide carboxypeptidase (EC 3.4.17.13)	4465.27	0	2340.92	0	0	0	0	4.35324	2.21584	0	0	0	0.292534
MutT/nudix family protein	0	0	0	0	0	0	0	10.3629	5.27481	0	0	0	0
Myosin-crossreactive antigen	0	0	0	0	8072.46	3364.18	0	0	0	0	0	0	0
N-Acetyl-D-glucosamine ABC transport system ATP-binding protein	0	341.33	153.677	0	0	0	0	0	0	0	0	0	0
N-Acetyl-D-glucosamine ABC transport system, sugar-binding protein	0	963.0771	533.9177	0	0	0	0	9.37102	4.602449	0	0	0	0
N-acetylglucosamine deacetylase (EC 3.5.1.-)	0	0	0	0	0	0	0	150.956	73.0864	0	0	0	0
N-acetylglucosamine-1-phosphate uridyltransferase eukaryotic (EC 2.7.7.23)	0	0	0	0	0	0	0	2.95944	1.50638	0	0	0	0
N-acetylglucosamine-regulated TonB-dependent outer membrane receptor	0	0	0	0	0	0	0	0	0	0.725864	0	0.372635	0
N-acetylmuramoyl-L-alanine amidase (EC 3.5.1.28)	0.313431	6.64073	0.164382	0	0	0	0	0	0	0	0	0	0
N-acetylneuraminate lyase (EC 4.1.3.3)	0.18115	0	0.0950056	0	0	0	0	0	0	0	0	0	0
N-acetyltransferase	0.673877	0	0.353421	0	124.935	52.3359	0	0	0	0	0	0	0
N-hydroxyarylamine O-acetyltransferase (EC 2.3.1.118)	0	0	0	0	0	0	0	0	0	42.2369	0	0	0
N-methyl-D-aspartate receptor glutamate-binding subunit	0	0	0	0	0	0	0	0	0	0.345935	0	0.177592	0
N-methylhydantoinase A (EC 3.5.2.14)	0.190361	0	0.0998364	0	0	0	0	90.3242	46.0642	0	0	0	0
N-methylhydantoinase B (EC 3.5.2.14)	0	74.7673	36.3356	0	0	0	1818.248	0	883.213	0	0.548543	0.266939	0
n-type ATP pyrophosphatase superfamily / TilS and TtcA-like	0	28.9758	21.4314	0	0	0	0	0	0	0	0	0	0
Na(+) H(+) antiporter subunit A; Na(+) H(+) antiporter subunit B	0	0	0	0	0	0	0	0	0	39.2525	0	19.6547	0
Na(+) H(+) antiporter subunit C	0	0	0	4808.3	0	20700.4	0	0	0	0	0	0	0
Na(+) H(+) antiporter subunit D	0	0	0	0	0	0	0	0	0	122.9026	0	73.8304	0
Na+/H+ antiporter NhaC	0	0	0	0	0	0	0	0.699472	0.356038	0	0	0	0
Na+/H+-dicarboxylate symporters	0	0	0	0	0	0	0	154.051	78.4694	0	0	0	0
NAD-dependent formate dehydrogenase alpha subunit; selenocysteine-containing	0	0	0	0	0	0	0	22.2819	0	0	0	0	0

NAD-dependent formate dehydrogenase beta subunit	0	0	0	0	0	0	0	0	0	82.415	0	56.417	0
NAD-dependent glyceraldehyde-3-phosphate dehydrogenase (EC 1.2.1.12)	0	40.3146	19.27039	1.94703	0	1.13198	0	0	0	2729.02443	0	1391.56817	0.991568
NAD-reducing hydrogenase subunit HoxF (EC 1.12.1.2)	0	0	0	0	0	0	0	14.1161	7.18523	0	0	0	0
NAD-reducing hydrogenase subunit HoxH (EC 1.12.1.2)	0	19.3711	9.62695	0	0	0	0	244.3217	127.8756	0	0	0	0
NAD(FAD)-utilizing dehydrogenases	1697.128	2.30602	891.27361	0	0	0	0	0	0	0	0	0	2.030931
NAD(P)H-quinone oxidoreductase chain I (EC 1.6.5.2)	0	0	0	0	0.336472	0.140852	0	0	0	0	0	0	0
NAD(P)HX epimerase / NAD(P)HX dehydratase	0.214953	0	0.112734	0	0	0	0	0	0	0	0	0	0
NADH dehydrogenase (quinone)	0	9.32596	4.43488	0	0	0	0	0	0	0	0	0	0
NADH dehydrogenase subunits 2, 5, and related proteins	0	0	0	0	0	0	56.293	0	27.6637	0	0	0	0
NADH ubiquinone oxidoreductase, 20 kDa subunit	0	0	0	0	0	0	0	22.9796	11.8148	0	0	0	0
NADH-dependent dehydrogenase	0	0	0	0	0	0	0	0	0	0	834.276	395.314	0
NADH-ubiquinone oxidoreductase chain C (EC 1.6.5.3)	9.18036	0	4.81472	0	0	0	0	0	0	0	0	0	0
NADH-ubiquinone oxidoreductase chain G (EC 1.6.5.3)	57.1958	0	30.0495	0	0	0	0	0	0	0	0	0	1.10428
NADH-ubiquinone oxidoreductase chain H (EC 1.6.5.3)	1063.405	0	558.666	21.3196	3.1282	13.704507	0	0	0	0	0	0	0.814864
NADH-ubiquinone oxidoreductase chain I (EC 1.6.5.3)	0	0	0	21.143	0	12.2923	0	0	0	0	0	0	0.628949
NADP-dependent malic enzyme (EC 1.1.1.40)	0	21.06	10.0416	0	0	0	0	0	0	0	0	0	1.473543
NADP-specific glutamate dehydrogenase (EC 1.4.1.4)	0	0	0	0	0	0	209.884	0	103.066	0	0	0	0
Nascent polypeptide-associated complex protein	0	0	0	0	0	0	0	16.2879	8.90482	0	0	0	0
Nef attachable protein	0	0	0	0	0	0	0	0	0	0	205.835	100.013	0
NfuA Fe-S protein maturation	0	0	0	0	0.317692	0.13299	0	0	0	0	0	0	0
Nickel ABC transporter, periplasmic nickel-binding protein NikA (TC 3.A.1.5.3)	0	0	0	4.1059	0	2.38711	0	0	0	0	0	0	0
Nickel-Cobalt-Cadmium resitance protein NccN	0	185.86	0	0	0	0	0	0	0	0	0	0	0
Nicotinate-nucleotide adenylyltransferase (EC 2.7.7.18)	0	0	0	0	0	0	0	0	0	0	24.4557	11.90095	0
Nitric-oxide reductase subunit B (EC 1.7.99.7)	0	0	0	0	0	0	0	29.2524	14.88976	0	0	0	0
Nitrite reductase large subunit (EC 1.7.1.4)	0	217.29	108.518	0	0	0	0	0	0	0	97.5023	38.6844	3.300591
Nitrogen regulatory protein P-II	0	0	0	10.28777	0	5.98117	0	0	0	0	0	0	0.342285
Nitrous oxide reductase maturation protein NosR	16.5446	1.40668	9.391175	0	0	0	0	0	0	0	0	0	1.298465
nucleoprotein/polynucleotide-associated enzyme	0	43.6909	20.7769	0	0	0	0	0	0	0	0	0	0
Nucleoside ABC transporter, periplasmic nucleoside-binding protein	0	40.4884	19.2539	0	0	0	0	0	0	0	0	0	0
Nucleoside triphosphate pyrophosphohydrolase MazG (EC 3.6.1.8)	0	0	0	0	0	0	0	9.03731	4.60007	0	0	0	0
Nucleoside-diphosphate-sugar epimerase	0	0	0	0	0	0	0	0.605147	0.308025	0	0	0	0
NUDIX hydrolase	0	0	0	0	0	0	0	0.401829	0.204535	0	0	0	0
Nudix hydrolase family protein PA3470	0	0	0	0	25.6616	9.90369	0	0	0	0	0	0	0
NusA protein homolog, archaeal	0	36.2708	17.5865	0	0	0	0	0	0	0	0	0	0
Oar protein	0	0.209942	0.0998364	0	0	0	0	0	0	0	0	0	0
OB-fold nucleic acid binding domain protein # COG1571	0	0	0	0	0	0	0	39.6023	20.05898	0	0	0	0
Octanoate--protein-N-octanoyltransferase	0	0	0	27.0205	0	15.67871	0	0	0	0	0	0	0.325318
Oligopeptide ABC transporter, oligopeptide binding protein	0	198.5041	94.3181	0	0	0	0	0	0	0	0	0	0
Oligopeptide ABC transporter, periplasmic oligopeptide-binding protein OppA (TC 3.A.1.5.1)	0	813.1172	388.85542	113.6171	0.275419	65.995694	93.57343	408.85217	248.35634	0	0	0	3.485597
Oligopeptide transport ATP-binding protein OppD (TC 3.A.1.5.1)	0	1060.73823	529.98384	0	0	0	1.19394	99.71	55.291911	0	0	0	0
Oligopeptide transport ATP-binding protein OppF (TC 3.A.1.5.1)	0.200559	52.8154	25.136485	0	0	0	353.209	37.1432	194.6612	0	0	0	0.224946
Oligopeptide transport system permease protein OppB (TC 3.A.1.5.1)	0	30.637	14.5692	0	0	0	0	429.97	221.3528	698.581	0	403.306	2.147311
Oligopeptide transport system permease protein OppC (TC 3.A.1.5.1)	0.197908	14.4585	7.150795	0	0	0	0	92.6479	47.1587	0	0	0	1.237566
Omega-amino acid--pyruvate aminotransferase (EC 2.6.1.18)	22.9118	0	0	0	0	0	0	0	0	0	0	0	0.458431
OmpA/MotB domain protein	0	0	0	0	0	0	3.16437	0	1.55368	0	0	0	0
Organosulfonate utilization protein SsuF	44.6556	0	23.42	0	0	0	0	0	0	0	0	0	0
Ornithine carbamoyltransferase (EC 2.1.3.3)	90.33	90.0022	90.5975	0	0	0	0	113.7417	57.7673	0	0	0	1.357569
OsmC-like protein	0	0	0	0	0	0	0	13.43614	6.83912	0	0	0	0
Osmolarity sensor protein EnvZ (EC 2.7.3.-)	0.336939	0	0.17671	0	0	0	0	0	0	0	0	0	0
Osmotically inducible lipoprotein B precursor	0	0	0	0	0	0	0	0	0	0	0.372639	0.181338	0
Outer membrane efflux family protein	0.510513	0	0.267743	0	0	0	0	0	0	0	0.359006	0.174704	0
Outer membrane hemin receptor	0	0	0	0	0	0	0	0	0	0	12.2274	5.95025	0
Outer membrane lipoprotein omp16 precursor	0	0	0	0	0	0	0	4.50133	2.294701	0	0	0	0
Outer membrane porin, OprD family	0	0	0	0	0	0.0901984	0	0	0	0	0	0	0
Outer membrane protein (porin)	504.44143	0	264.78828	0	0	0	0	0	0	0	0	0	9.097231
Outer membrane protein A precursor	0	0	0	0	0	0	0	4.48923	2.69247	95.7759	0	45.8567	0
Outer membrane protein assembly factor YaeT precursor	55.7283	0	29.659	0	0	0	0	0	0	0	0	0	1.630004
Outer membrane protein H precursor	39.3651	0	21.9867	0	0	0	9.7077	1.783768	5.690468	0	0	0	0.164646
Outer membrane protein Imp, required for envelope biogenesis / Organic solvent tolerance protein precurs	17.3297	0	9.08872	0	0	0	0	0	0	0	0	0	0.406136
Outer membrane protein W precursor	0	0	0	32.3556	0	58.2607	0	0	0	0	0	0	1.501355
Outer membrane usher protein FIMD	0	0	0	243.889	0	140.524	0	0	0	0	0	0	0
Oxaloacetate decarboxylase alpha chain (EC 4.1.1.3)	0	0	0	0	0	0	0	0	0	0	767.477	373.578	0
Oxidoreductase FAD/NAD(P)-binding:Oxidoreductase FAD-binding region precursor	2.24554	0	1.177693	0	0	0	0	0	0	0	0	0	0
Oxidoreductase of aldo/keto reductase family, subgroup 1	0	0	0	0	0	0	0	0	0	32.2149	0	17.7349	0
Oxidoreductase, Gfo/Idh/MocA family	0	0	0	0	0	0	0	40.815	20.7752	0	0	0	0
Para-aminobenzoate synthase, aminase component (EC 2.6.1.85) / Aminodeoxychorismate lyase (EC 4.1.3.3	0	0	0	219.724	0	127.745	0	0	0	0	0	0	0.241671
PAS/PAC sensor hybrid histidine kinase	0	0	0	0	0	0	0	0	0	1.47518	0	0.757307	0
PblB	76.617	0	59.6717	0	0	0	0	0	0	0	0	0	0
PCTP-like protein	0	0	0	0	0	0	0	44.4752	22.547	0	0	0	0
PE-PGRS family protein	4472.69	0	0	0	0	0	0	0	0	0	0	0	0
PEP-utilizing enzyme, TIM barrel domain protein	0	0	0	0	0	0	0	12.8065	6.51862	0	0	0	0
Peptidase C1A, papain	0	0	0	0	0	0	0	1.12464	0	0	0	0	0
Peptidase M20	0	0	0	0	0	0	0	0	0	0	0.240314	0.116945	0
Peptidase M23B precursor	0	0	0	0	0	0	0	9.07643	4.61999	0	0	0	0
Peptidase M28	0	57.9002	27.9835	0	0	0	0	0	0	0	0	0	0
Peptidase S8 and S53, subtilisin, kexin, sedolisin	0	0	0	0	0	0	683.822	105.39679	54.108	0	0	0	0
Peptidase T (EC 3.4.11.4)	0	0	0	0	0	0	0	0	0	169.642	0	89.7354	0
Peptidase, M16 family	0	0	0	0	0	0	0	0	0	1581.658	0	223.6029	0
Peptidase, M23/M37 family	0	0	0	0	1799.661	750.133	0	31.3933	16.07074	0	0	0	0
Peptidase, M42 family	0	31.9902	15.1153	0	0	0	0	0	0	0	0	0	0
Peptide chain release factor 2; programmed frameshift-containing	0	61.5374	29.2636	0	0	0	0	0	0	0	0	0	1.01683
Peptide peptide ABC transporter, periplasmic peptide-binding protein	0	4.54368	2.25705	0	0	0	0	0	0	0	0	0	0
Peptide/opine/nickel uptake family ABC transporter, periplasmic substrate-binding protein	543.075	0	284.953	0	0	0	0	0	0	0	0	0	0
Periplasmic alpha-galactoside-binding protein precursor	0	67.6942	33.1229	0	0	0	0	0	0	0	0	0	0
Periplasmic aromatic aldehyde oxidoreductase, FAD binding subunit YagS	0	0	0	0	0	0	295.181	0	144.779	0	0	1.50395	0
Periplasmic aromatic aldehyde oxidoreductase, molybdenum binding subunit YagR @ 4-hydroxybenzoyl-Co/	0	0	0	0	0	0	0	0	0	221.312	0	114.6404	0
Periplasmic binding protein	0	0	0	0	0	0	0	220.884	112.48	0	0	0	0
Periplasmic nitrate reductase precursor (EC 1.7.99.4)	0	0	0	0	0	0	0	1.64198	0.897696	0	0	0	0
Periplasmic serine endoprotease	0	0	0	0	0	0	0	0	0	0	0	0	0
Periplasmic sugar-binding protein, putative	0.218083	0	0.114376	0	0	0	0	0	0	0	0	0	0
Permease of the drug/metabolite transporter (DMT) superfamily	874.886	0	458.401	0	0	0	0	1.67788	0.854057	0	0	0	7.856286
Permease of the major facilitator superfamily	0	0	0	0	0	0	1595.39	0	206.938	0	0	0	0
Permeases of the major facilitator superfamily / Thymidylate kinase (EC 2.7.4.9)	0	0	0	429.595	0	251.042	0	0	0	0	0	0	0
Peroxidase	0	0	0	0	0	0	0	0	0	54.3675	0	27.9105	0
PF00070 family, FAD-dependent NAD(P)-disulphide oxidoreductase	0	12.6381	6.00995	0	0	0	0	0	0	0	0	0	1.657131
pH adaptation potassium efflux system a	0	0	0	0	0	0	0	0	0	53.1756	0	27.6866	0
Phage capsid protein	0	0	0	8.48398	0	4.93247	0	0	0	0	0	0	0
Phage integrase family protein	74.9892	0	39.4183	0	0	0	0	0	0	0	0	0	0.347305
Phage lysin, glycosyl hydrolase, family 25	0	0	0	0	0	0	0	0	0	15.2306	0	7.81887	0
Phage shock protein C	0	0	0	0	0	0	0	0	0	2153.4	0	1478.62	0
Phage T7 exclusion protein	0	0	0	0	0	0	0	27.1429	14.31918	0	0	0	0
Phage tail length tape-measure protein	0	0	0	377.901	0	218.122	0	0	0	0	0	0	0
Phage tail tape measure	0	0	0	12.9572	0	7.91803	0	0	0	0	0	0	0
Phage terminase, large subunit	0.313431	0	0.164382	0	0	0	0	0	0	0	0	0	0
phage-related integrase	0	0	0	0	0	0	0	0	0	0	65.7402	32.7174	0
Phage-related protein	0	0	0	0	0	81.0979	6.71499	0	3.29699	0	0	0	0.403172
Phenylacetate-coenzyme A ligase (EC 6.2.1.30)	0	14.8639	7.00748	0	0	0	0	0	0	0	0	0	0.811253
Phenylalanyl-tRNA synthetase alpha chain (EC 6.1.1.20)	217.24	0	114.156	0	0	0	0	2.53097	1.33542	0	0	0	0.346528
Phenylalanyl-tRNA synthetase domain protein (Bsu YtpR)	0	0	0	0	0	0	0	0	0	49.0897	0	22.9299	0
Phosphatidylserine decarboxylase (EC 4.1.1.65)	0	138.2144	67.5924	0	0	0	0	0	0	0	0	0	1.538942
Phosphatidylserine/phosphatidylglycerophosphate/ cardiolipin synthases and related enzymes-like protein	0	0	0	0	0	0	3.63043	0	3.28207	0	0	0	0
Phosphoenolpyruvate synthase (EC 2.7.9.2)	0	343.402	170.0092	0	0	0	1052.24	241.25241	652.05263	0	0	0	1.425262
Phosphoenolpyruvate-dihydroxyacetone phosphotransferase (EC 2.7.1.121), ADP-binding subunit DhaL	0	2.68164	1.27523	0	0	0	0	326.636	167.236	0	0	0	0
Phosphoenolpyruvate-dihydroxyacetone phosphotransferase (EC 2.7.1.121), dihydroxyacetone binding subu	0	285.064	135.665	0	0	0	188.8944	0	98.9639	0	0	0	0
Phosphoenolpyruvate-protein phosphotransferase of PTS system (EC 2.7.3.9)	0.154914	6.66971	3.2529661	0	0	0	0	19.0635	9.32709	87.5592	0	44.95	0.482457
Phosphoesterase	0	0	0	0	0	0	0	2.38619	1.2146	0	0	0	0
Phosphoglucomutase (EC 5.4.2.2)	0	0	0	0	0	0	0	0	0	1283.423	0	1739.507	0
Phosphogluconate dehydratase (EC 4.2.1.12)	0	0	0	0	32.8712	13.6889	0	0	0	0	0	0	0.161269
Phosphoglycerate mutase (EC 5.4.2.1)	0	0	0	259.923	0	2350.79	0	0	0	0	0	0	0.573497
Phosphoglycolate phosphatase (EC 3.1.3.18)	44.8505	0	23.5223	0	0	0	0	0	0	0	0	0	0.363554
Phosphohydrolase	0	0	0	0	0	0	0	0	0	0.225044	0	0.11553	0
Phosphomannomutase (EC 5.4.2.8)	1.96883	0	1.03257	0	478.458	191.024	0	6.07053	3.08996	0	0	0	0
Phosphonate ABC transporter phosphate-binding periplasmic component	2139.1392	0	1120.045	1281.307	0	743.967	0	0	0	0	0	0	1.206184
Phosphoribosyl-AMP cyclohydrolase (EC 3.5.4.19) / Phosphoribosyl-ATP pyrophosphatase (EC 3.6.1.31)	0	0	0	2.57391	0	1.571199	0	0	0	0	0	0	0
Phosphoserine phosphatase (EC 3.1.3.3)	0.218083	156.009	73.242976	0	0	0	0	0	0	374.953	0	192.489	0.502712
Phytoene dehydrogenase (EC 1.14.99.-)	0	0	0	0	0	0	0	0	0	0	110.0304	58.6956	0
Pirin family protein	0	0	0	0	8.30928	3.47838	0	0	0	0	0	0	0
PKD repeat protein	0	0	0	0	0	0	0	65.2365	33.206	0	0	0	0
Plasmid conjugative transfer endonuclease	0	0	0	0	0	0	2.99654	0	0	0	0	0	0

Plasmid replication protein RepA	0	0	0	0	0.389565	0.163077	0	0	0	0	0	0	0
Pol protein	0	0	0	0	0	0	0	0	0	188.692	0	157.563	0
Poly-gamma-glutamate synthase subunit PgsC/CapC (EC 6.3.2.-)	0	0	0	0	0	0	0	0	0	501.446	0	257.558	0
Poly(A) polymerase (EC 2.7.7.19)	0	0	0	79.5018	0	45.6693	0	0	0	0	0	0	0.683679
Poly(glycerol-phosphate) alpha-glucosyltransferase (EC 2.4.1.52)	0	0	0	0	0	0	0	0	0	0	0	309.59	0
Polyprotein	0	0	0	0	1047.95	0	0	0	0	0	0	0	0
Possible hydrolase or acyltransferase RutD in novel pyrimidine catabolism pathway	32.4812	0	17.0351	0	0	0	0	0	0	0	0	0	0
Possible linoleoyl-CoA desaturase	304.259	0	158.803	0	0	0	0	0	0	0	0	0	0
Possible periplasmic aspartyl protease	0.187971	0	0.0985832	0	0	0	0	0	0	0	0	0	0
Possible protein-tyrosine phosphatase	0	0	0	0	0	0	0	0	0	0	0.416582	0.202723	0
Possible transcriptional regulator, ArsR family	0	0	0	25.3135	0	14.7169	0	0	0	0	0	0	0
Potassium efflux system KefA protein / Small-conductance mechanosensitive channel	902.449	0	280.018	0	0	0	0	0	0	0	0	0	18.896444
Potassium-transporting ATPase A chain (EC 3.6.3.12) (TC 3.A.3.7.1)	6.27128	443.717	216.86706	0	0	0	0	0	0	26.8057	0	31.3331	1.153653
Predicted 2-keto-4-pentenoate hydratase/2-oxohepta-3-ene-1,7-dioic acid hydratase	0	0	0	0	0	0	166.955	0	81.8018	0	0	0	0
Predicted amidohydrolase	0	0	0	0	0	0	0	13.7104	7.22993	0	0	0	0
Predicted ATPase	0	0	0	0	0	0	0	0	0	624.464	0	2006.142	0
Predicted cell-wall-anchored protein SasF (LPXAG motif)	0	0	0	0	0	0	0	0	0	53.8639	0	27.4854	0
Predicted chaperone lipoprotein YacC, potentially involved in protein secretion	0	0	0	0	0	0	0	0	0	0	0.247382	0.120384	0
Predicted deacylase	0	0	0	0	0	0	0	0.475528	0.242048	0	0	0	0
Predicted dye-decolorizing peroxidase (DyP), YfeX-like subgroup	0.211911	0	0.111139	0	0	0	0	0	0	0	0	0	0
Predicted L-rhamnose ABC transporter, substrate-binding component	0	8.41808	4.00315	0	0	0	0	0	0	0	0	0	0
Predicted membrane protein	0	0	0	0	9.77451	4.09174	0	0	0	1.99324	0	1.02327	0
Predicted membrane protein hemolysin III homolog	0	0	0	0	0	0	0	76.3556	39.1911	0	0	0	0
Predicted metal-dependent hydrolase	0	0	0	0	0	0	0	2.77557	1.38663	0	0	0	0
Predicted metal-dependent hydrolase of the TIM-barrel fold	0	0	0	1.14373	0	0.664951	0	0	0	0	0	0	0
Predicted methyltransferase	0	0	0	0	0.418187	0.175059	0	0	0	0	0	0	0
Predicted nucleotidyltransferase of the DNA polymerase beta superfamily	0	0	0	0	0	0	0	30.417	15.54458	0	0	0	0
Predicted O-methyltransferase	0	0	0	0	0.268913	0.11257	0	0	0	0	0	0	0
Predicted Permease	0	11.5744	5.54272	0	0	0	0	0	0	0	0	0	0.590871
Predicted phosphohydrolase	0	0	0	0	0	0	0	247.655	126.496	0	0	0	0
Predicted reductase RutE in novel pyrimidine catabolism pathway	33.0868	0	17.3526	0	0	0	0	0	0	0	0	0	0
Predicted RNA methylase COG2263	0	0	0	0	0	0	0	46.60106	25.29631	0	0	0	0
Predicted signal transduction protein	1.421269	1.610957	1.511473	0	0	0	0	0	0	0	0	0	0
Predicted signal-transduction protein containing cAMP-binding and CBS domains	2.50912	0	1.31593	0	0	0	0	40.590785	20.666152	0	0	0	0
Predicted steroid reductase	0	0	0	19.35586	0	17.16595	0	0	0	0	0	0	0
Prepro-subtilisin sendai, putative	0	71.6101	0	0	0	0	0	0	0	0	0	0	0
Preprotein translocase subunit SecE (TC 3.A.5.1.1)	184.631	0	95.1486	0	0	0	0	0	0	0	0	0	0
Pro-pol-dUTPase polyprotein - murine endogenous retrovirus ERV-L (fragment)	0	0	0	0	0	0	0	0	0	496.819	0	259.288	0
Probable activation/secretion signal peptide protein	0	60.1717	29.3436	0	0	0	0	0	0	0	0	0	0.723591
Probable ATP-dependent RNA helicase	0	0	0	0	0	0	0	0	0	0	1.96649	0.956959	0
Probable c-type cytochrome biogenesis protein	0	0	0	0	0	0	0	10.0908	5.13632	0	0	0	0
Probable cation-transporting ATPase	0	0	0	0	0	0	0	4.11935	2.09679	0	0	0	0
Probable Co/Zn/Cd efflux system membrane fusion protein	0	0	0	22.17262	4.99391	15.82613	273.088411	0	134.151984	0	0	0	4.320987
Probable dinitrogenase reductase activating glycohydrolase (draG)(EC:3.2.2.24)	0	0	0	0	0	0	0	0.296011	0.150673	0	0	0	0
Probable exosome complex exonuclease 2 (EC 3.1.13.-)	0	0	0	0	0	0	0	13.48146	6.86359	0	0	0	0
Probable exosome complex RNA-binding protein 1	0	0	0	0	0	0	21.9241	0	10.7645	0	0	0	0
Probable HesA/MoeB/ThiF family protein	0	55.0696	26.1106	0	0	0	0	0	0	0	0	0	0
Probable hippurate hydrolase(EC:3.5.1.32)	0	0	0	0	0	0	0	0	0	0	1.70165	0.828077	0
Probable iron-sulfur binding protein YPO1417	0.192812	0	0.101122	0	0	0	0	0	0	0	0	0	0
Probable membrane protein YPO1482	0	0	0	1.083937	0	0.630187	0	0	0	0	0	0	0
Probable NADH-dependent dehydrogenase	0	0	0	0	0	0	0	148.36133	85.64791	0	0	0	0
Probable oxidoreductase/Short-chain dehydrogenase	0	0	0	43.2313	0	25.1341	0	0	0	0	0	0	0
Probable phage replication protein NMA0782	0	0	0	0	0	0	0	0	0	0	984.957	479.312	0
Probable phospholipase	0	0	0	0	0	0	0	0	0	0	54.6926	26.8068	0
Probable poly(beta-D-mannuronate) O-acetylase (EC 2.3.1.-)	0.429906	0	0.225468	0	0	0	0	0	0	0	0	0	0
Probable protease HtpX (EC 3.4.24.-)	3145.7	0	1638.24	155.891	0	90.2597	0	0	0	0	0	0	0.314717
Probable ribonuclease inhibitor YPO3690	0	0	0	0	0	0	0	11.9258	9.35655	0	0	0	0
Probable secreted glycosyl hydrolase	0	0	0	0	0	0	0	0	0	233.854	0	119.973	0
Probable serine peptidase (U7 family) protein	0	6.6578	3.31332	0	0	0	0	0	0	0	0	0	0
Probable serine proteinase	0	0	0	0	0	0	295.717	0	145.922	0	0	0	0
Probable serine/threonine-protein kinase pknL (EC 2.7.11.1)	0	0	0	11.3396	0	0	0	0	0	0	0	0	0
probable streptomycin biosynthesis protein strI	0	0	0	0	0	0	0	47.5572	24.6668	0	0	0	0
Probable tonB-dependent receptor yncD precursor	0.218083	0	0.114376	0	0	0	0	0	0	0	0	0	0
Probable transmembrane protein	280.3554	275.626	219.7352	3416.06	0	2005	0	37.7415	19.2108	0	0	0	41.251898
Probable transporter	0	0	0	0	0	0	0	0	0	0	0.455234	0.221532	0
Probable transposase for insertion sequence element IS702	0	0	0	0	0	0	0	503.447	33.1381	0	0	0	0
Proline/sodium symporter PutP (TC 2.A.21.2.1) @ Propionate/sodium symporter	0	0	0	0	0	0	25.1787	0	16.56926	0	0	0	0
prolyl-tRNA synthetase ProS	24136	0	12659.7	0	0	0	0	0	0	0	0	0	0
Prophage LambdaCh01, recombination protein Bet	0.371282	0	0.194722	0	0	0	0	0	0	0	0	0	0
Propionyl-CoA carboxylase beta chain (EC 6.4.1.3)	0	33.4566	15.91	0	0	0	0	0	0	0	0	0	0
Proposed lipoate regulatory protein YbeD	0	0	0	35.6095	0	20.70286	0	0	0	0	0	0	0.559744
Proteasome subunit alpha (EC 3.4.25.1), archaeal	0	41.1306	19.503	0	0	0	0	0	0	0	0	0	0
Proteasome subunit beta (EC 3.4.25.1), archaeal	0	0	0	0	0	0	0	73.9903	37.6618	0	0	0	0
Protein containing plastocyanin/azurin family domain	0	1.91545	0.971603	0	0	0	0	0	0	0	0	0	0
Protein export cytoplasm chaperone protein (SecB, maintains protein to be exported in unfolded state)	0	0	0	0	0	0	0	0	0	0	3870.94	1481.134	0
Protein export cytoplasm protein SecA ATPase RNA helicase (TC 3.A.5.1.1)	6.28952	0.474128	3.524058	0	338.967	141.197	124.8651	0	61.3075	0	0	0	30.150975
Protein hipA	0.359401	0	0.188491	0	0	0	0	0	0	0	0	0	0
Protein involved in mRNA turnover and stability	0	0	0	0	0	0	0	370.614	189.2816	0	0	0	0
Protein involved in stability of MscS mechanosensitive channel	3.11456	0	116.28896	0	0	0	0	0	0	0	0	0	0.280781
Protein of unknown function DUF222	0	0	0	43.705	0	26.3645	0	0	0	0	0	0	0
Protein of unknown function DUF224(EC:1.8.98.1)	0	0	0	0	0	0	0	118.979	56.2325	0	0	0	0
Protein of unknown function DUF28	0	6.09177	2.65548	0	0	0	0	0	0	0	0	0	0
Protein of unknown function DUF881	0	0	0	0	0	0	0	770.355	395.35	0	0	0	0
Protein of unknown function DUF91	0.208954	0	0.109588	0	0	0	0	0	0	0	0	0	0
Protein RtcB	0	165.67043	80.23331	0	0	0	0	0	0	0	0	0	0
Protein similar to polyadenylation specificity factor, MJ1236 type	0	772.444	367.112	0	0	0	0	0	0	0	0	0	0
Protein yceI precursor	21.159	18.68697	20.253126	0	31.4993	13.2207	0	0	0	0	0	0	0.806345
Protein yraQ	0	240.485	114.3782	0	0	0	0	0	0	0	0	0	0
Protein-export membrane protein SecF (TC 3.A.5.1.1)	16.0301	0	8.40713	0	0	0	0	0	0	0	0	0	1.230665
Protoporphyrin IX Mg-chelatase subunit D (EC 6.6.1.1)	0	0	0	0	0	0	0	1.91198	0.973216	0	0	0	0
PTS system HrsA EIIA component / PTS system HrsA EIIB component / PTS system HrsA permease IIC compo	0	83.374	39.6798	0	0	0	0	0	0	0	0	0	0
PTS system, beta-glucoside-specific IIB component (EC 2.7.1.69) / PTS system, beta-glucoside-specific IIC cor	0.214953	0	0.112734	0	0	0	0	0	0	0	0	0	0
PTS system, mannose-specific IIC component	0	0	0	0	0	0	232	0	113.888	0	0	0	0
PTS system, N-acetylglucosamine-specific IIA component / PTS system, N-acetylglucosamine-specific IIB com	0	0	0	0	0.275419	0.115294	0	0	0	0	0	0	0
PTS system, sucrose-specific IIB component (EC 2.7.1.69) / PTS system, sucrose-specific IIC component (EC 2	0	0	0	0	0	0	0	0	0	239.3976	0	154.5449	0
Putative ABC transporter	0	0	0	0	0	0	8.82766	0	4.28459	0	0	0	0
Putative ABC transporter ATP-binding protein	1142.7806	20.44388	609.403163	0	0	0	0	0	0	0	0	0	0.200622
putative ABC transporter permease	0	0	0	0	0	0	11.6994	0	0	0	0	0	0
Putative ABC transporter solute-binding protein	0	373.494	0	0	0	0	0	0	0	0	0	0	0
Putative acyl carrier protein phosphodiesterase	0	0	0	23.02543	0	13.38668	0	0	0	0	0	0	0
Putative amidase	0	0	0	0	0	0	0	3.83185	1.835719	0	0	0	0
Putative amino acid ABC transporter, permease protein	0.76577	0	0.401614	0	0	0	0	0	0	0	0	0	0
Putative amino acid deaminase	0	0	0	63.224	0	36.7539	0	0	0	0	0	0	0
Putative aminotransferase	0	0	0	0	0	0	0	7.40893	3.55224	0	0	0	0
Putative antibiotic transport-associated protein	0	0	0	0	0	0	0	0	0	54.485	0	28.5252	0
Putative ATP-dependent Clp proteinase (ATP-binding subunit)	0	0	0	0	0	0	0	0	0	1670.399	0	873.1612	0
Putative ATP/GTP-binding protein	0	0	0	233.2132	0	134.9651	0	0	0	0	0	0	0.231231
Putative ATPase component of ABC transporter with duplicated ATPase domain	0	0	0	25.1248	0	14.6072	0	0	0	0	0	0	0
Putative bacitracin ABC transporter, ATP-binding protein BcrA	0	0	0	0	0	0	0	0	0	407.47	0	209.311	0
Putative bacterial transferase hexapeptide repeat:ADP-glucose pyrophosphorylase	0	0	0	0	0	0	10.6284	0	5.21843	0	0	0	0
Putative binding-protein-dependent transport protein (periplasmic)	0	0	0	0	0	0	0	10.033	5.10504	0	0	0	0
Putative branched-chain amino acid ABC transporter (substrate-binding protein)	0	0	0	0	0	0	0	8.93428	42.85933	0	0	0	0
Putative CarD-like transcriptional regulator	0	0	0	0	0	0	0	0	0	1016.06	0	572.437	0
Putative cytochrome c 553	0	9.80758	4.43935	0	0	0	0	0	0	0	0	0	0
Putative deoxyribose-specific ABC transporter, permease protein	0	13.3356	6.34162	0	0	0	0	0	0	0	0	0	1.038456
Putative divalent heavy-metal cations transporter	0	0	0	0	18.0564	7.59285	0	0	0	0	0	0	0
putative DNA helicase	0.214953	0	0.112734	0	0	0	0	0	0	0	0	0	0
putative DNA polymerase III epsilon subunit (exonuclease)	0	0	0	0	0	0	0	0	0	0	307.535	0	0
putative esterase	0	0	0	0	0	0	0	0	0	0.242656	0	0.124572	0
putative exported protein	0	0	193.198	0	0	0	0	105.86568	58.38947	3766.44	0	1947.41	6.713567
Putative exported protein precursor	0	0	0	0	0	0	0	10.5948	6.03822	0	0	0	0
Putative fucose-glucose oligosaccharide ABC transport system, sugar-binding protein	0	0	0	0	0	0	0	147.445	77.2031	0	0	0	0
Putative fumarate reductase flavoprotein subunit	0	0	0	0	0	0	1989.99	0	1009.85	0	0	0	0
Putative gluthatione transporter,solute-binding component	2.716404	0	1.424642	0	0	0	0	0	0	0	0	0	0
Putative glycosyl transferase	0.276633	0	0.145082	0	0	0	0	0	0	0	0	0	0
Putative GTPases (G3E family)	0.460299	0	0	0	0	0	0	0	0	0	0	0	0
Putative HTH-type transcriptional regulator ycaN	0.326728	0	0.171356	0	0	0	0	0	0	0	0	0	0
Putative hydrolases or acyltransferases (Alpha/beta hydrolase superfamily)	0.257598	0	0.1351	0	0	0	0	0	0	0	0	0	0
Putative integral membrane efflux protein	0	0	0	0	0	0	0	0	0	0	94.7955	44.9629	0

Putative integral membrane protein	0	0	0	0	0	0	396.668	415.864	406.493	0	0	0	0
Putative integration host factor IHF alpha subunit	0	0	0	0	0	0	0	0.588982	0.299797	0	0	0	0
Putative isobutyryl-CoA mutase, chain B	0	19.9538	13.6302	0	0	0	0	0	0	0	0	0	0
Putative lipoprotein LpqB	0	0	0	0	1855.832	657.457	0	0	0	0	0	0	0
Putative major facilitator superfamily (MFS) transporter	0.174806	0	0.0916785	0	0	0	0	0	0	0	0	0	0
Putative membrane-bound ClpP-class protease associated with aq_911	0	754.316	374.706	0	0	0	0	0	0	0	0	0	0.767273
Putative methionine aminopeptidase	0	0	0.210369	0	0	0	0	0	0	0	0	0	0
Putative multi-domain protein	0	0	0	0	0	0	0	19.0827	9.77398	0	0	0	0
Putative nucleotide-binding protein	0	92.5516	45.5178	0	0	0	0	0	0	0	0	0	0
Putative oligopeptide ABC transport system membrane protein	0	0	0	3630.509	0	486.958	0	0	0	0	0	0	0
Putative opine dehydrogenase	0	0	0	0	0	0	0	1.05954	0.539314	0	0	0	0
Putative peptidoglycan binding domain 1	0	0	0	0	0	0	0	0	0	0.418581	0	0.214886	0
Putative periplasmic protein kinase ArgK and related GTPases of G3E family	0	0	0	0	0	0	0	0	0	0.263259	0	0.135148	0
Putative phage excisionase	0	0	0	94.6336	0	126.8891	0	0	0	0	0	0	0
Putative phosphatidylglycerophosphate synthase	0.371897	0	0.195045	0	0	0	0	0	0	0	0	0	0
Putative phosphoenolpyruvate synthase/pyruvate phosphate dikinase, C-terminal domain	0	789.112	378.85	0	0	0	0	3.9825	2.16747	0	0	0	0
Putative phosphotransferase system protein	0	376.284	0	0	0	0	0	0	0	0	0	0	0
Putative plasmid-related protein	0	0	0	0	0	0	0	0	0	98.864	0	50.4175	0
Putative protein phosphatase	0	0	0	0	2988.16	1252.64	0	0	0	0	0	0	0
Putative pyridine nucleotide-disulphide oxidoreductase	0	0	0	0	0	0	0	0	0	0.667304	0	0.342572	0
Putative secretion accessory protein EsaA/YueB	0	0	0	0	0	0	0	0	0	78.8728	0	40.4755	0
Putative senescence marker protein-30 (SMP-30) (Regucalcin) (RC)	0	0	0.0938701	0	0	0	0	0	0	0	0	0	0
Putative sensory transduction histidine kinase	5.05702	0	2.6522	0	0	0	0	0	0	0	0	0	0
Putative stomatin/prohibitin-family membrane protease subunit aq_911	0	203.1329	100.8688	0	0	0	564.501	2501.10578	1798.995079	0	0	0	0.207164
Putative sucrose phosphorylase (EC 2.4.1.7)	0	0	0	0	0.695401	0.291104	0	0	0	0	0	0	0
Putative sugar isomerase	0	0	0	0	0	0	0	129.099	66.3288	0	0	0	0
Putative superfamily I DNA helicases	0	0	0	0	315.094	132.1598	0	0	0	0	0	0	0
putative TonB-dependent receptor	0	0	0	0	0.336472	0.140852	0	0	0	0	0	0	0
Putative transcriptional regulator	0	0	0	0	0	0	0	0.94496	0.4527	0	0	0	0
Putative transferase	0	0	0	0	0	0	0	0	0	0	0.547693	0.266525	0
Putative transmembrane protein	0	0.134637	0.0640255	92.8901	0	54.1418	0	0	0	0	0	0	4.163868
Putative transporter	0.211911	0	0.111139	0	0	0	0	0	0	0	0	0	0
Putative transporting ATPase	0	0	0	45.7063	0	26.573	0	0	0	0	0	0	0
Putative transposable element	19.896	0	10.7401	0	0	0	0	0	0	0	0	0	0
Putative tRNA-m1A22 methylase	32.7803	0	17.1919	0	0	0	0	0	0	0	0	0	0
Putative two-component system response regulator	0.423822	0	0.222277	0	0	0	0	35.6473	18.8042	0	0	0	0
Putative two-component system sensor protein, no kinase domain	0	0	0	0	0	0	0	9.8022	4.97854	0	0	0	0
Putative UDP-glucuronosyltransferase	0	0	0	0	0	0	0	0	0	405.755	0	801.3	0
Putative uncharacterized protein Sb07g003556	0.409901	0	0.214976	0	0	0	0	0	0	0	0	0	0
Putative virion core protein (lumpy skin disease virus)	0	0	0	0	0	0	0	602.4901	306.67349	0	0	0	0
Pva1 protein	0	0	0	0	0	0	0	0	0	17.758	0	10.0931	0
Pyridine nucleotide-disulphide oxidoreductase family protein	0	0	0	0	0	0	0	24.0314	12.22088	0	0	0	0
Pyrogallol hydroxytransferase(EC:1.97.1.2)	0	0	0	0	0	0	0	25.0853	12.7687	0	0	0	0
Pyrophosphate-energized proton pump (EC 3.6.1.1)	0	154.5689	73.9344	0	0	0	0	0	0	0	0	0	0
Pyruvate dehydrogenase E1 component beta subunit (EC 1.2.4.1)	0	0	0	300.907	0	176.175	0	0	0	0	0	0	0
Pyruvate formate-lyase (EC 2.3.1.54)	0	0	0	0	0	0	0	11.7767	6.12643	0	0	0	0
Pyruvate formate-lyase activating enzyme (EC 1.97.1.4)	0	0	0	0	0	0	0	26.1353	13.06767	0	0	0	0
Pyruvate,phosphate dikinase (EC 2.7.9.1)	0	8.476432	4.030901	0	0	0	0	0	0	0	0	0	0
Pyruvate:ferredoxin oxidoreductase, alpha subunit (EC 1.2.7.1)	0	243.357	111.545	0	0	0	0	0	0	108.3285	0	61.3515	0
Pyruvate:ferredoxin oxidoreductase, beta subunit (EC 1.2.7.1)	0	0	0	0	0	0	0	1.35701	0.690729	0	0	0	0
Pyruvate:ferredoxin oxidoreductase, gamma subunit (EC 1.2.7.1)	0	224.58798	106.78721	0	0	0	0	0	0	0	0	0	0
Quaternary ammonium compound-resistance protein SugE	1584.616	343.855	941.378	0	0	0	0	0	0	0	0	0	0
Queuosine Biosynthesis QueC ATPase	0	81.2319	38.6771	0	0	0	0	0	0	0	0	0	1.011531
Radical SAM domain protein	73.9163	236.295	150.8757	11.10326	0	6.5647	0	10.5296	2.53422	0	0	0	0.541941
RecA protein	0	37.5581	17.8604	0	0	0	0	0.200563	0.102088	402.141	0	207.5167	0.848083
RecD-like DNA helicase YrrC	0	0	0	0	0	0	0	40.6957	22.73605	0	0	0	0
Recombination protein RecR	0	0	0	132.43	0	77.7802	0	0	0	0	0	0	0.478989
Redox-sensing transcriptional regulator QorR	0	6.55408	3.11674	0	0	0	0	0	0	0	0	0	0
Reductases with broad range of substrate specificities	0	0	0	0	0	0	0	0	0	0	1634.008	755.957	0
Regulatory protein, MarR	104.8612	0	47.6514	0	0	0	0	0	0	0	0	0	0
Replication-associated protein	0	0	0	0	0	0	0	1.58811	0.808362	0	0	0	0
Repressor CsoR of the copZA operon	0	0	0	188.8313	0	109.784	0	0	0	0	0	0	0
Resolvase, N-terminal domain	0	0	0	0	0	0	27.1439	0	12.5058	0	0	0	0
Resolvase/Integrase TinR protein	0	14.9034	7.06372	0	0	0	0	0	0	0	0	0	0
Response regulator	12.3859	0	6.49589	0	0	0	0	0	0	0	0	0	0.40215
Response regulator of the LytR/AlgR family	0	0	0	3.27425	0	1.9036	0	0	0	0	0	0	0
Response regulator receiver	0	0	0	0	0	0	0	0	0	0	470.945	0	0
Retron-type reverse transcriptase	0	0	0	0	525.939	218.531	661.363	608.542	707.6049	0	0	0	0.802072
Retron-type RNA-directed DNA polymerase (EC 2.7.7.49)	0	18920.2708	12592.62	3955.41	0	0	43140.8	6744.591	30243.5894	23824.7	1342.575	9651.41	0
Retrotransposon protein, putative, LINE subclass	0	0	0	0	0	0	0	0	0	112.27	0	113.62	0
Retrotransposon protein, putative, Ty1-copia subclass	99.783	0	52.3688	0	0	0	0	0	0	378.305	0	201.5603	0
Retrotransposon protein, putative, Ty3-gypsy subclass	0	467.023	339.927	0.242269	0	0.140852	0	0	0	3671.67	0	1890.785	0
Retrotransposon protein, putative, unclassified	145.625	0	76.3978	0	0.44546	0.186475	0	0	0	1428.257	0	748.405	0
Retrotransposon, putative, centromere-specific	0	0	0	0	0	0	0	0	0	14.5701	0	7.47981	0
Reverse transcriptase	0	235.683	321.716	0	0	0	0	0	0	0	0	0	0
Rhamnogalacturonides degradation protein RhiN	0	0	0	0	0	0	0	15.0764	7.88074	0	0	0	0
Rhamnulokinase (EC 2.7.1.5)	0	0	0	0	0	0	435.685	0	208.964	0	0	0	0
Rhs family protein	0	0	0	0	0	0	0	17.18446	8.74705	0	0	0	0
Ribonuclease BN (EC 3.1.-.-)	0	0	0	0	0	0	0	0	0	9.30844	0	4.77865	0
Ribonuclease HII (EC 3.1.26.4)	3.11654	0	1.60365	0	0	0	0	0	0	0	0	0	0
Ribonuclease III family protein	0	0	0	0	0	0	0	0	0	85.2358	0	43.7392	0
Ribonuclease J1 (endonuclease and 5' exonuclease)	0	0	0	0	0	0	0	0	0	467.823	0	239.986	0
Ribonuclease P protein component 1 (EC 3.1.26.5)	0	0	0	0	0	0	0	38.2961	19.4931	0	0	0	0
Ribonuclease PH (EC 2.7.7.56)	0	17.44458	8.28221	0	0	0	0	0	0	0	0	0	1.104813
Ribonuclease Y (EC 3.1.-.-)	0	0	0	0	0	0	0	0	0	117.9399	0	63.0805	0
Ribonucleotide reductase of class Ia (aerobic), beta subunit (EC 1.17.4.1)	0.813125	0	0.42645	0	0	0	0	0	0	0	0	0	0
Ribonucleotide reductase of class II (coenzyme B12-dependent) (EC 1.17.4.1) @ intein-containing	0	0	0	0	0	0	0	0	0	79.8504	0	43.1825	0
Ribonucleotide reductase of class III (anaerobic), large subunit (EC 1.17.4.2)	9.09627	0	4.77062	2185.14	0	1308.94	0	3.40095	1.73112	55.2917	0	28.5182	1.211452
Ribonucleotide reductase transcriptional regulator NrdR	0.206079	0	0.10808	0	0	0	15.155	0	7.34718	0	0	0	0
Ribose ABC transport system, periplasmic ribose-binding protein RbsB (TC 3.A.1.2.1)	0	90.5387	43.0549	0	0	0	0	328.7639	179.1278	0	0	0	0.72263
Ribose ABC transporter (permease)	0	71.6863	54.5552	0	0	0	0	0	0	0	0	0	0
Ribose-phosphate pyrophosphokinase (EC 2.7.6.1)	0	14.5222	7.10904	0	0	0	0	38.5768	20.3557	31.1547	0	17.6815	0.243779
Ribosomal large subunit pseudouridine synthase A (EC 4.2.1.70)	0	0	0	48.5986	0	28.8662	0	0	0	0	0	0	0
Ribosomal large subunit pseudouridine synthase C (EC 4.2.1.70)	0	0	0	47.8035	0	31.6237	0	0	0	0	0	0	0
Ribosome recycling factor	682.04	164.387	432.1989	0	0	0	0	0	0	0	0	0	0.200622
Ribulokinase (EC 2.7.1.16)	0	50.4516	23.9918	0	0	0	0	0	0	0	0	0	0
Ribulose bisphosphate carboxylase large chain (EC 4.1.1.39)	0	0	0	0	0	0	0	2.55776	1.30193	0	0	0	0
Rieske (2Fe-2S) region	0	0	0	0	0	0	0	0	0	0	20.5407	9.99577	0
RNA 3'-terminal phosphate cyclase (EC 6.5.1.4)	0	0	0	0	0	0	0	0	0	225.422	0	125.1996	0
RNA binding methyltransferase FtsJ like	0	0	0	0	0.272127	0.113916	0	0	0	0	0.281708	0.137088	0
RNA methyltransferase, TrmA family	0	0	0	0	3.04701	1.27552	0	0	0	0	0	0	0
RNA polymerase associated protein RapA (EC 3.6.1.-)	0.271725	0	0.142508	0	0	0	0	0	0	0	0	0	0
RNA polymerase principal sigma factor HrdD	0	0	0.179401	0	0	0	0	0	0	0	0	0	0
RNA polymerase sigma factor for flagellar operon	0	181.424	86.1666	0	0	0	0	0	0	0	0	0	1.227755
RNA polymerase sigma factor RpoH	35.8202	0	18.5574	0	0	0	0	0	0	0	0	0	0
RNA-binding protein	0	0	0	0	0	0	1711.01	0.46709	840.240753	0	0	0	0
RNA-binding region RNP-1 (RNA recognition motif)	0	0	0	0	0	0	0	3.28095	1.67003	0	0	0	0
RNA:NAD 2'-phosphotransferase TPT1	0	0	0	0	0	0	0	0	0	2522.27	0	1298.7	0
RNase L inhibitor	97.8095	0	51.3202	0	0	0	0	0	0	0	0	0	0
RND efflux system, inner membrane transporter CmeB	0	0	0	81.6106	0	50.7203	0	0	0	0	0	0	3.55
Rod shape-determining protein MreD	0.163364	50.8405	24.3503778	0	0	0	0	0	0	0	0	0	1.056059
rRNA methylase	0	0	0	0	0	0	0	0	0	18.7159	0	11.1024	0
rRNA small subunit methyltransferase H	0	3217.62	1530.14	0	0	0	0	0	0	32.1358	0	16.4974	0
rRNA small subunit methyltransferase I	0.214953	0	0.112734	0	0	0	0	0	0	0	0	0	0
rRNA small subunit methyltransferase, glucose inhibited division protein GidB	0	9.09017	4.32276	0	0	0	0	0	0	0	0	0	0
RTX toxin, putative	0	0	0	0	0	0	0	1.39425	0.575422	0	0	0	0
RTX toxins and related Ca2+-binding proteins	0	69.5694	32.8258	0	0	0	0	0	0	0	0	0	0
S-adenosylhomocysteine deaminase (EC 3.5.4.28); Methylthioadenosine deaminase	0	0	0	0	0	0	0	31.6089	14.9036	0	0	0	0
S-adenosylmethionine decarboxylase proenzyme (EC 4.1.1.50), prokaryotic class 1A	0.174806	0	0.0916785	0	0	0	0	0	0	0	0	0	0
Salicylate hydroxylase (EC 1.14.13.1)	0.176871	0	0.0927613	0	0	0	0	0	0	0	0	0	0
SAM-dependent methyltransferase YafE (UbiE paralog)	0	0	0	0	2152.31	900.282	0	0	0	0	0	0	0.398069
SAM-dependent methyltransferases	0	0	0	107.5376	0	65.3611	0	0	0	0	0	0	1.596304
Sarcosine oxidase alpha subunit (EC 1.5.3.1)	0.176871	0	0.0927613	0	0	0	0	0	0	0	0	0	0
Secreted protein containing C-terminal beta-propeller domain distantly related to WD-40 repeats	0	0	0	0	0	0	0	1145.1084	581.8242	0	0	0	0
Secreted protein, suppressor for copper-sensitivity D	0	0	0	0	0	0	0	0	0.158972	0	0	0	0
Secretion activator protein	0	0	0	0	0	0	0	44.1905	22.6818	0	0	0	0
Sensor histidine kinase	8214.3818	0	4299.02411	0	0	0	0	0	0	0	0	0	4.155983
Sensor histidine kinase VraS	0	0	0	0	0	0	0	0	0	562.469	0	288.753	0
Sensor protein	0	0	0	5.60429	0	3.39125	0	0	0	0	0	0	0

Sensor protein basS/pmrB (EC 2.7.3.-)	0	0	0	2.10921	0	1.22626	0	0	0	0	0	0	0
Sensor protein copS (EC 2.7.3.-)	0	0	0	0	0	0	0	0	0	0	184.5819	89.9195	0
Sensory box protein	0	0	0	0	2036.75	855.076	0	0	0	0	0	0	0
Sensory transduction histidine kinase	0	0	0	0	1.01441	0.424647	0	0	0	0	0	0	0
Septin family protein (P-loop GTPase)	80697.9	0	41186.7	0	0	0	0	0	0	0	0	0	0
Serine hydroxymethyltransferase (EC 2.1.2.1)	0	0	0	115.261	0	266.136	0	18.56021	9.67447	391.237	0	158.143	0.802487
Serine protease	0	62.5413	28.8995	0	0	0	0	0	0	0	0	0	0
Serine--pyruvate aminotransferase (EC 2.6.1.51)	0	0	0	0	0	0	0	28.1623	7.23064	0	0	0	0
Serine-threonine kinase Stk1	0	95.3613	44.4364	0	0	0	0	0	0	0	0	0	0
Serine/threonine protein kinase (EC 2.7.11.1)	566.394	89.8677	341.8318	0	0	0	0	0	0	0	0	0	1.416913
Serine/threonine protein kinase PrkC, regulator of stationary phase	0.29299	64.2941	30.607261	11.9099	0	6.94292	0	0	0	0	0	0	0
Serine/threonine protein kinase related protein	0	85.5295	41.2619	0	0	0	0	0	0	0	0	0	0
Serine/threonine protein phosphatase 2 (EC 3.1.3.16)	3.86915	0	2.02921	0	0	0	0	0	0	0	0	0	0
Seven transmembrane helix receptor	0	0	0	0	0	0	0	0	0	29.7477	0	15.494	0
Shikimate 5-dehydrogenase I alpha (EC 1.1.1.25) # AroDI alpha	0.403519	0	0.211629	0	0	0	0	0	0	0	0	0	0
Short-chain dehydrogenase/reductase SDR	10.473703	0	5.427204	0	0	0	0	0	0	0	0	0	1.992358
Shwachman-Bodian-Diamond syndrome protein; predicted exosome subunit	0	50.5	24.0808	0	0	0	0	0	0	0	0	0	0
Siderophore biosynthesis complex, short chain @ Siderophore synthetase small component, acetyltransfera	0	0	0	0	0	0	0	49.7531	25.8816	0	0	0	0
Sigma cross-reacting protein 27A	0	0	0	0	0	0	0	16.8166	8.55982	0	0	0	0
Sigma factor sigB regulation protein rsbU	0	0	0	0	0	0	0	8.8434	4.50137	0	0	0	0
Signal peptidase I (EC 3.4.21.89)	0	0	0	25.6731	0	14.92602	0	0	0	0	0	0	2.276178
Signal recognition particle receptor protein FtsY (alpha subunit) (TC 3.A.5.1.1)	11.6584	41.656	26.46854	0	0	0	47.7176	0	23.0575	0	0	0	0.448589
Single-stranded DNA-binding protein	0	150.236	71.4437	0	0	0	6.17009	34.8486	25.63901	3162.54	0	1628.25	1.7954528
Sirtuin 5 and related class III sirtuins (SIR2 family)	0	0	0	0	0	0	0	0	0	0	0.332013	0.161568	0
Site-specific recombinase XerD	0	0	0	0	0	0	0	192.269	98.4404	0	0	0	0
Small heat shock protein	0	4.12585	1.960086	0	0	0	0	1340.305	698.129	0	0	0	0
Small integral membrane protein	0	0	0	0	0	0	0	0	0	22.3244	0	11.46058	0
Sodium:dicarboxylate symporter	0	0	0	20.5608	0	11.9538	0	156.801	78.2268	0	0	0	0.147381
Sodium/alanine symporter family protein	0	0	0	0	0	0	0	0	0	6.3866	0	3.27868	0
Sorbitol-6-phosphate 2-dehydrogenase (EC 1.1.1.140)	0	0	0	297.387	0	174.37	0	0	0	0.496538	0	0.254906	0
Sphingolipid fatty acid hydroxylase	0	538.928	257.361	0	0	0	0	0	0	0	0	0	0
Spore coat protein A	0	113.404	55.0783	0	0	0	0	0	0	0	0	0	0
SpoVT/AbrB-like protein	0	0	0	0	0	0	0	0	0	43.2104	0	22.10896	0
SSU ribosomal protein S11e (S17p)	0	0	0	0	0	0	0	28.7971	15.5596	0	0	0	0
SSU ribosomal protein S11p (S14e)	0	0	0	0	0	0	0	0	0	48.8722	0	27.4899	0
SSU ribosomal protein S12p (S23e)	0	214.679	101.897	0	0	0	0	0	0	0	0	0	0.416522
SSU ribosomal protein S14p (S29e) @ SSU ribosomal protein S14p (S29e), zinc-dependent	0	0	0	0	0	0	0	0	0	5.95818	0	3.05874	0
SSU ribosomal protein S15Ae (S8p)	0	0	0	0	0	0	0	23.3154	11.8678	0	0	0	0
SSU ribosomal protein S17p (S11e)	0	0	0	0	0	0	0	53.347	27.7519	0	0	0	0
SSU ribosomal protein S18p @ SSU ribosomal protein S18p, zinc-dependent	0	0	0	0	0	0	9.87526	0	4.84866	0	0	0	0
SSU ribosomal protein S19e	0	49.8873	23.9397	0	0	0	0	0	0	0	0	0	0
SSU ribosomal protein S19p (S15e)	0	0	0	0	0	0	0	0.608142	0.30955	3.50765	0	1.800708	0
SSU ribosomal protein S23e (S12p)	0	0	0	0	46.5534	19.48794	0	0	0	0	0	0	0
SSU ribosomal protein S27e	0	572.309	259.147	0	0	0	0	0	0	0	0	0	0
SSU ribosomal protein S28e	0	0	0	0	0	0	0	57.6702	29.3547	0	0	0	0
SSU ribosomal protein S5p (S2e)	0	101.928	51.5228	0	0	0	0	0	0	1821.706	0	935.721	0.918783
SSU ribosomal protein S6e	0	0	0	0	0	0	0	1.23301	0.627616	0	0	0	0
SSU ribosomal protein S6p	0	0	0	0	0	0	0	3.15763	1.60726	0	0	0	0
SSU ribosomal protein S7p (S5e)	0	38.7547	16.3059	0	0	0	0	0	0	11.662	0	5.98687	0
SSU ribosomal protein S8p (S15Ae)	0	82.0459	38.0248	0	0	0	0	6.00369	2.11565	0	0	0	0
SSU rRNA (adenine(1518)-N(6)/adenine(1519)-N(6))-dimethyltransferase (EC 2.1.1.182)	0	0	0	0	0	0	0	11.06541	3.53218	0	0	0	0
Streptomycin 3''-O-adenylyltransferase (EC 2.7.7.47) @ Spectinomycin 9-O-adenylyltransferase	12678.9	0	6190.9	0	0	0	0	0	0	0	0	0	0
Substrate-specific component TTE1586 of predicted methylthioadenosine ECF transporter	1421.218	0	745.538	0	0	0	0	0	0	0	0	0	0
Subtilisin BPN' precursor (EC 3.4.21.62)	0	0	0	0	0	0	0	0	342.609	0	0	0	0
Subtilisin-like serine protease	0	0	0	0	0	0	0	12.23788	3.46303	0	0	0	0
Succinate dehydrogenase flavoprotein subunit (EC 1.3.99.1)	0	2075.92	1113.35	0	0	0	182.843	0	88.9494	5636.5138	0	2334.1078	1.671011
Succinyl-CoA ligase alpha chain (EC 6.2.1.5)	6676.44	0	3502.24	0	0	0	0	0	0	0	0	0	1.031903
Succinyl-CoA ligase beta chain (EC 6.2.1.5)	2405.5708	168.086	1341.779387	0	0	0	107.108	0	52.3736	0	0	0	1.740021
Sugar ABC transporter sugar-binding protein	0	0	0	0	0	0	0	0	16.4527	0	0	0	0
Sugar ABC transporter, ATP-binding protein	0.178985	0	0	0	0	0	0	0	0	0	0	0	0
Sugar ABC transporter, periplasmic sugar-binding protein	0.190361	9.66729	4.7188064	0	0	0	0	0	0	0	0	0	0
Sugar binding protein of sugar ABC transporter	0	200.232	109.868	0	0	0	0	0	0	0	0	0	0
Sugar efflux transporter B	0	0	0	0	0.275419	0.115294	0	0	0	0	0	0	0
Sugar kinases, ribokinase family	0	4972.6	2365.06	0	0	0	0	0	0	0	0	0	1.210339
Sulfate and thiosulfate binding protein CysP	2577.3	0	1352.587	0	0	0	0	0	0	0	0	0	0.534618
Sulfopyruvate decarboxylase - beta subunit (EC 4.1.1.79)	0	0	0	0	0	0	14.6559	0	7.1959	0	0	0	0
Sulfur deprivation response regulator	0	0	0	0	0	0	0	3.48771	1.77528	0	0	0	0
Superfamily II DNA/RNA helicases, SNF2 family	5.81519	0	3.04982	0	0	0	0	3.90742	1.98891	0	0	0	0
Superoxide dismutase (EC 1.15.1.1)	0	35.3938	16.7967	0	0	0	0	0	0	0	0	0	1.868711
Surface layer protein B (slgB-2)	0	0	0	0	0	0	0	3.8368	1.9701	0	0	0	0
Surface protein Rib	504.849	0	125.326	0	0	0	0	0	0	0	0	0	0
SusC, outer membrane protein involved in starch binding	0	0	0	0	0.225673	0.0944699	0	0	0	0	0	0	0
Symbiosis island integrase	0	0	0	0	0	0	0	146.981	76.5145	0	0	0	0
Synaptic vesicle transporter SVOP and related transporters (major facilitator superfamily)	0	0	0	0	0	0	0	0	0	0.245863	0	0.126218	0
Syntenic homolog of Saccharomyces cerevisiae YNR018W	0	0	0	0	0	0	0	0	0	0	214.792	17.8157	0
T-complex protein 11	0	0	0	93.3691	0	54.3641	0	0	0	0	0	0	0
Teichoic acid export ATP-binding protein TagH (EC 3.6.3.40)	0	0	0	0	0	0	4.56469	0	2.186977	0	0	0	0
Terminase large subunit	0	0.272233	0	0	0	0	0	0	0	0	0	0	0
Tetracycline-resistance determinant TetV	78.5499	0	23.5614	0	0	0	0	0	0	0	0	0	0
Tetrathionate reductase sensory transduction histidine kinase	0	0	0	0	0	0	0	3885.58	1978.61	0	0	0	0
Thermosome subunit	402.04	42.5237	18.2758	0	0	0	0	60.2254	32.196	0	0	0	0
Thermostable carboxypeptidase 1 (EC 3.4.17.19) ## Zinc-requiring, any residue but Pro	0	0	0	0	0	0	0	78.4229	39.918	0	0	0	0
Thiamine-monophosphate kinase (EC 2.7.4.16)	0.200559	0	0.105185	0	190.105	79.7163	0	3.76647	2.50707	0	0	0	0.526317
Thioredoxin	0	274.67	130.646	0	0	0	0	0	0	0	0	0	1.937493
Thioredoxin reductase (EC 1.8.1.9)	5675.0094	0	3066.7674	0	0	0	0	81.0373	38.2804	0	0	0	2.131271
Thiosulfate sulfurtransferase, rhodanese (EC 2.8.1.1)	23.3857	0	8.51276	0	0	0	0	0	0	0	0	0	0.806829
Threonine dehydrogenase and related Zn-dependent dehydrogenases	0	0	0	0	0	0	0	7.02601	3.57631	0	0	0	0
Threonine efflux protein	0	0	0	0	551.115	230.705	0	0	0	0	0	0	0
Threonine synthase (EC 4.2.3.1)	0.203281	4.5599	2.275033	0	0	0	0	6.74214	3.45368	236.298	0	121.953	0.541765
Threonyl-tRNA synthetase (EC 6.1.1.3)	0	80.3988	38.4394	0	180.0704	46.509394	0	0	0	0	0	0	0.589236
Thymidine kinase (EC 2.7.1.21)	0	0	0	0	0	0	23.1832	0	11.322	0	0	0	0
Thymidylate synthase thyX (EC 2.1.1.-)	0	0	0	0	0	0	334.634	0	159.478	0	0	0	0
TldD protein, part of TldE/TldD proteolytic complex	0	5.67208	2.639	0	0	0	0	0	0	0	0	0	0.863986
TldE protein, part of TldE/TldD proteolytic complex	0	15.58528	7.32596	0	0	0	0	0	0	0	0	0	0
TldE/PmbA family protein, Actinobacterial subgroup	0	426.097	203.8718	0	0	0	0	0	0	0	0	0	0
TniB NTP-binding protein	4.95378	0	2.72904	0	0	0	31.0874	0	15.26403	0	0	0	0
TonB protein	0	31.8173	15.2434	0	0	0	0	0	0	0	819.348	371.4217	0
TonB-dependent receptor	7474.0688	337.793	238.956814	561.895	0.304928	317.260647	1653.1151	2749.48463	2209.5633	382.264	355.932	366.146	6.924508
TonB-dependent receptor, putative	0	0	0	0	0	0	0	12.86085	6.54033	0	0	0	0
TonB-dependent receptor; Outer membrane receptor for ferrienterochelin and colicins	0	0	0	0	0	0	25.54736	0	12.68811	0	0	0	0
Topoisomerase IV subunit A (EC 5.99.1.-)	13.3871	0	7.03491	0	0	0	0	111.652	57.3438	0.86052	0	0.441763	0.768274
TPR domain protein	0	0	0	0	0	0	0	0	0	2.95153	0	1.515221	0
Transcription accessory protein (S1 RNA-binding domain)	786.15398	0	404.83081	0	0	0	0	34.008	18.29121	0	0	0	1.014447
Transcription antitermination protein NusG	98.7098	70.8312	85.4524	0	0	0	0	0	0	0	0	0	0.430366
Transcription attenuation protein MtrB	0	0	0	0	0	0	0	0	60.6945	0	0	0	0
Transcription initiation factor B	0	2340.277	2642.273	0	0	0	0	35.338729	18.763253	0	0	0	0
Transcription regulator, ArsR family NMB0398	0	1423.47	719.476	0	0	0	0	0	0	0	0	0	0
Transcription termination protein NusA	0	0	0	0	0	0	0	19.7543	9.73076	0	0	0	0
Transcriptional (co)regulator CytR	0	35.5204	16.3934	0	0	0	0	0	0	0	0	0	0
Transcriptional antiterminator and PTS system component IIA	0	0.781489	0.408794	0	0	0	0	0	0	0	0	0	0
Transcriptional regulator, AraC family / Acetamidase/Formamidase	0	0	0	0	7.79062	3.26126	0	0	0	0	0	0	0
Transcriptional regulator, PadR family	0	0	0	4.87741	0	2.71751	0	274.61142	2.28618	0	0	0	0.390305
Transcriptional regulator, RpiR family	0	0	0	0	0	0	0	1.66758	0.877106	0	0	0	0
Transcriptional regulator, XRE family	0	65.8316	31.3995	0	0	0	0	0	0	0	0	0	1.59242
Transcriptional regulatory protein	0	0	0	0	0	0	0	20.9313	0	0	0	0	0
Transcriptional repressor of aga operon	374.125	0	192.16	0	0	0	0	0	0	0	0	0	0
Transcriptional repressor of PutA and PutP / Proline dehydrogenase (EC 1.5.99.8) (Proline oxidase) / Delta-1	2636.37	0	1381.98	0	0	0	0	0	0	0	0	0	1.528164
Transglutaminase-like	0	0	0	0	0	0	0	0	1.05049	0	0	0	0
Transitional endoplasmic reticulum ATPase	0	0	0	0	0	0	14.1096	0	6.92769	0	0	0	0
Translation elongation factor 1 alpha subunit	0	9.3399	46.26841	0	0	0	0	2.46511	1.19203	0	0	0	0
Translation elongation factor 2	1623.67	1413.5258	1559.2107	447.187	0	272.554	0	0	0	0	0	0	0
Translation elongation factor 3	0	0	0	21.9395	0	12.6242	0	0	0	0	0	0	0
Translation elongation factor EF-1 alpha/Tu	0	90.8271	0	0	0	0	0	0	0	0	0	0	0
Translation elongation factor G	0	0	0	397.071	0	232.1183	0	30.564451	15.557612	102.1109	0	69.3604	4.903153
Translation elongation factor G ## mitochondrial	1592.06	0	865.977	0	0	0	0	0	0	0	0	0	0
Translation elongation factor P	0	597.087	272.1132	0	0	0	0	0	0	0	0.277722	0.135148	0.739562
Translation initiation factor 1A	0	146.974	69.8923	0	0	0	0	0	0	0	0	0	0
Translation initiation factor 3	0	0	0	0	0	0	0	139.881	70.5604	448.5169	0	230.4157	0
Translation initiation factor 4F, ribosome/mRNA-bridging subunit (eIF-4G)	0	0	0	0	910.592	382.45	0	0	0	0	0	0	0

Translation initiation factor SUI1-related protein	0	0	0	0	0	0	0	4.90445	3.49498	0	0	0	0
Transmembrane protein MT2276, clustered with lipoate gene	0	0	0	0	0	0	0	0	0	0	0.324212	0	0
Transmembrane protein Tmp5	0	0	0	0	0	0	0	49.7547	21.89833	0	0	0	0
Transposase	33079.633	145.1721	17407.0887	315.75667	0	193.79029	133.5779	0	65.5538	0	0	0	68.121658
Transposase DDE domain protein	0	0	0	0	0	0	0	18.44648	9.34922	0	0	0	0
Transposase ECs0136	0	0	0	0	0	0	189.5688	0	92.4456	0	0	0	0
Transposase in ISPg1	0	0	0	119.706	0	151.031	0	0	0	0	0	0	0
Transposase, IS4	5.75396	0	0	0	0	0	0	0	0	8.83903	0	4.53767	0
Transposase, mutator type	0	0	0	0	0	0	0.98915	0	1.33557	0	0	0	0
Transposon protein, putative, CACTA, En/Spm sub-class	27.3135	0	13.2934	0	0	0	0	0	0	0	0	0	0
TRAP dicarboxylate transporter, DctM subunit, unknown substrate 3	0	0	0	0	0	0	0	43.75736	23.996773	0	0	0	0
TRAP dicarboxylate transporter, DctM subunit, unknown substrate 5	0	0	0	0	0	0	0	52.89188	26.91668	0	0	0	0
TRAP dicarboxylate transporter, DctM subunit, unknown substrate 6	0	0	0	0	0	0	0	44.344	20.9375	0	0	0	0
TRAP transporter solute receptor, TAXI family precursor, unknown substrate 2	0	0	0	0	0	0	0	207.543	116.327	0	0	0	0
TRAP transporter solute receptor, unknown substrate 1	0	0	0	0	0	0	0	81.504	32.421	0	0	0	0
TRAP transporter solute receptor, unknown substrate 3	0	75.5782	36.1042	0	0	0	233.631	35.3945	132.4117	0	0	0	0
TRAP transporter solute receptor, unknown substrate 6	0	0	0	0	0	0	0	25.98133	12.98333	0	0	0	0
TRAP transporter, 4TM/12TM fusion protein, unknown substrate 2	0	0	0	0	0	0	0	127.467	63.1483	0	0	0	0
TRAP-type C4-dicarboxylate transport system, large permease component	0	0	0	0	0	0	0	0.867925	0.373816	0	0	0	0
TRAP-type C4-dicarboxylate transport system, periplasmic component	0	0	0	0	0	0	2.02309	2037.6852	1039.44592	0	0	0	0
Trehalose synthase (EC 5.4.99.16)	677.006	0	355.108	0	0	0	0	0	0	0	0	0	3.207508
Tricarboxylate transport membrane protein TctA	0.178985	0	0.0938701	0	0	0	0.744271	0	0.36543	0	0	0	0
Tricarboxylate transport protein TctC	0	0	0	0	0	0	0	68.123	34.7087	0	0	0	0
Trimethylamine-N-oxide reductase (EC 1.6.6.9)	0	0	0	1.16245	0	0.675832	0	0	0	0	0	0	0
Trimethylamine:corrinoid methyltransferase; pyrrolysine-containing	0	0	0	0	0	0	0	142.6433	72.7442	0	0	0	0
Triosephosphate isomerase (EC 5.3.1.1)	0	44.5056	21.3041	0	0	0	0	0	0	0	0	0	0
Tripeptide aminopeptidase (EC 3.4.11.4)	0	0	0	0	0	0	0	3.8389	6.47623	0	0	0	0
Trk system potassium uptake protein TrkA	0	0	0	0	0	0	0	4.41948	2.258214	0	0	0	0
tRNA N2,N2-dimethyl(Guanine26-N2)-methyltransferase (EC 2.1.1.32)	0	0	0	0	0	0	0	0	0	70.6624	0	36.2757	0
tRNA-dependent lipid II-Ala--L-alanine ligase	0	0	0	0	0	0	0	10.1207	8.1359	0	0	0	0
tRNA-i(6)A37 methylthiotransferase	0	0	0	0	0	0	0	0	0	58.7232	0	31.6281	0
Tryptophan 2-monooxygenase (EC 1.13.12.3)	0	44.7027	21.258	0	0	0	0	0	0	0	0	0	0
Tryptophan synthase beta chain like (EC 4.2.1.20)	0	0	0	0	0	0	587.4324	494.4672	480.32008	0	0	0	0
TspB protein	0	0	0	0	0	0	4.46029	0	2.20437	127.805	55.9689	47.447	0
Tungsten-containing aldehyde:ferredoxin oxidoreductase (EC 1.2.7.5)	0	193.0028	89.25551	0	0	0	0	226.34529	116.71013	0	0	0	0
Tungsten-containing formate dehydrogenase alpha subunit	0	0	0	0	0	0	0	16.1258	7.87906	0	0	0	0
Twin-arginine translocation protein TatC	0	2255.87	1501.13	0	0	0	0	0	0	0	0	0	0.427856
Two component transcriptional regulator VraR	0	0	0	0	0	0	0	0	0	428.316	0	222.619	0
Two component transcriptional regulator, winged helix family	0	0	0	0	0	0	0	0	0	0	0.395147	0.192292	0
Two component transcriptional regulatory protein DevR	0	0	0	4.13866	0	8.55328	0	0	0	0	0	0	0
Two component, sigma54 specific, transcriptional regulator, Fis family	0	39.2097	18.6458	0	0	0	0	0	0	0	0	0	0
Two-component response regulator CreC	0	65.8038	32.0288	0	0	0	0	0	0	0	0	0	0
Two-component sensor histidine kinase	0	0	0	0	0	0	0	2.20069	0.902827	0	0	0	0
Two-component system sensor histidine kinase/response regulator, hybrid ('one-component system')	0	0	0	0	0	0	0	2.6884	1.368419	0	0	0	0
Type cbb3 cytochrome oxidase biogenesis protein CcoG, involved in Cu oxidation	0	14.63871	6.81725	0	0	0	0	0	0	0	0	0	1.0413224
Type I restriction-modification system, DNA-methyltransferase subunit M (EC 2.1.1.72)	3.82969	0	2.00851	110.898	0	64.6862	0	0	0	0	0	0	0.383048
Type I restriction-modification system, restriction subunit R (EC 3.1.21.3)	63.72077	0	33.41892	0	0	0	6.91041	2.86883	4.87171	4.96585	0.270078	2.680729	10.631895
Type I restriction-modification system, specificity subunit S (EC 3.1.21.3)	0	901.14	431.094	0	0	0	0	0	0	0	0	0	0.229543
Type I secretion outer membrane protein, TolC precursor	0.368239	0	0.193126	1439.15	0	839.702	0	0	0	0	0	0	0.698055
Type I signal peptidase	0	100.162	47.7372	0	0	0	0	0	0	0	0	0	0
Type II restriction enzyme HpaII (Endonuclease HpaII)(EC:3.1.21.4)	4991.95	0	2619.6	0	0	0	0	0	0	0	0	0	0
Type II restriction enzyme, methylase subunit YeeA	7.184251	731.123	350.361848	0	0	0	0	0	0	0	0	0	0
Type II secretory pathway, ATPase PulE/Tfp pilus assembly pathway, ATPase PilB	0	0	0	11.3539	0	7.52445	0	0	0	0	0	0	0.859371
Type III restriction-modification system methylation subunit (EC 2.1.1.72)	0	0	0	0	0	0	0	0	0	0	47.2334	22.9425	0
Type IV fimbrial assembly, ATPase PilB	0	10.5988	5.04019	0	0	0	0	312.6458	159.2867	0	0	0	0.3662
Type IV pilus biogenesis protein PilM	0	0	0	0	0	0	0	21.7664	11.0793	0	0	0	0
Type IV pilus biogenesis protein PilP	62.5372	0	32.7982	0	0	0	0	0	0	0	0	0	0
Type IV pilus biogenesis protein PilQ	95.2618	0	0	0	0	0	0	0	0	0	0	0	1.293975
Tyrosine recombinase XerC	0	0	0	0	0	0	0	1330.02	676.826	0	0	0	0
Tyrosyl-tRNA synthetase (EC 6.1.1.1)	0	95.56158	47.52163	0	0	0	0	48.2934	24.6013	0	0	0	0.29761
Ubiquitin-specific protease	17225.2	0	9652.09	0	0	0	0	0	0	0	0	0	0
UDP-2,3-diacylglucosamine diphosphatase (EC 3.6.1.54)	8.64668	0	4.53483	0	0	0	0	0	0	0	0	0	0.35467
UDP-3-O- glucosamine N-acyltransferase (EC 2.3.1.-)	0	0	0	0	0	0	0	3.81069	3.01161	0	0	0	0
UDP-3-O- glucosamine N-acyltransferase (EC 2.3.1.191)	28.0434	0	14.7076	0	0	0	0	0	0	0	0	0	0.326303
UDP-galactopyranose mutase (EC 5.4.99.9)	0	0	0	0	0	0	4.8862	0	2.39908	0	0	0	0
UDP-glucose 4-epimerase (EC 5.1.3.2)	0	101.476	47.7731	0	0	0	0	26.9314	13.70831	0	0	0	9.170099
UDP-glucose dehydrogenase (EC 1.1.1.22)	0	0	0	0	0	0	0	17.681248	9.037581	0	0	0	0
UDP-N-acetylenolpyruvoylglucosamine reductase (EC 1.1.1.158)	0	48.87219	23.50806	0	0	0	0	34.567364	17.593386	0	0	0	0.187187
UDP-N-acetylglucosamine 4,6-dehydratase (EC 4.2.1.-)	0	0	0	0	0	0	0	48.3832	24.6275	0	0	0	0
UDP-N-acetylmuramate:L-alanyl-gamma-D-glutamyl-meso-diaminopimelate ligase (EC 6.3.2.-)	0	453.757	225.6563	0	0.278791	0.116706	0	0	0	0	0	0	0.714503
UDP-N-acetylmuramoylalanyl-D-glutamate--2,6-diaminopimelate ligase (EC 6.3.2.13)	0.481201	25.1603	12.5047701	0	0	0	0	0	0	0	0	0	0.389552
Uncharacterized 9.4 kDa protein	0.724599	0	0.304018	0	0	0	0	0	0	0	0	0	0
Uncharacterized ATP-dependent helicase AF1149	0	0	0	0	0	0	0	203.758	109.035	0	0	0	0
Uncharacterized conserved protein, contains double-stranded beta-helix domain	0.255257	0	0.133871	0	0	0	0	0	0	0	0	0	0
Uncharacterized mitochondrial protein AtMg00030	0	0	0	0	0	0	0	0	0	26.9287	0	0	0
Uncharacterized protein (ATP-grasp superfamily)	0	4.46995	2.12565	0	0	0	0	0	0	0	0	0	0
Uncharacterized protein AF_0817 precursor	0	0	0	0	0	0	0	104.1553	7.00228	0	0	0	0
Uncharacterized protein MJ0274	0	0	0	0	0	0	0	0	0	125.6793	0	64.9775	0
Uncharacterized protein MJ1313	0	0	0	0	0	0	0	19.7092	10.0322	0	0	0	0
Uncharacterized protein similar to VCA0109	19.30071	0	9.88419	0	0	0	0	0	0	0	0	0	0
Uncharacterized protein, homolog of B.subtilis YhgC	0	0	0	0	0	0	0	0	0	32.32798	0	16.82796	0
Undecaprenyl-phosphate galactosephosphotransferase (EC 2.7.8.6)	0	19.2796	9.16824	0	0	0	0	0	0	0	0	0	0.671048
Undecaprenyl-phosphate N-acetylglucosaminyl 1-phosphate transferase (EC 2.7.8.-)	0	0	0	0	0	0	0	0	0	147.4101	0	75.5595	0
UPF0246 protein YaaA	0	0	0	0	0	0	0	0	0	0	0.294385	0.143257	0
Uracil phosphoribosyltransferase (EC 2.4.2.9)	1.472956	0	0.772504	0	0	0	0	0	0	84.3862	0	43.3211	0
Urea ABC transporter, urea binding protein	109.1722	0	32.7732	0	0	0	0	0	0	0	0	0	0
Urea carboxylase (EC 6.3.4.6)	0	1.62852	0.774428	0	0	0	0	9.29636	4.73194	0	0	0	0
Urease accessory protein UreF	0	0	0	0	0	0	0	0	0	111.756	0	33.8892	0
Uridine phosphorylase (EC 2.4.2.3)	0	0	0	131.8725	0	84.8683	0	0	0	0	0	0	0
Uridylate kinase (EC 2.7.4.-)	25.3642	0	13.3156	0	0	0	0	0	0	0	0	0	0
Uridylyltransferase (EC 2.7.7.59)	0	0	0	42.6961	0	78.1409	0	5.91807	3.01235	0	0	0	2.773962
Uroporphyrinogen decarboxylase (URO-D)	0	0	0	4.4214	0	2.9931	0	0	0	0	0	0	0
Uroporphyrinogen-III decarboxylase	0	0	0	0	0	0	0	5.48748	1.714	0	0	0	0
Uroporphyrinogen-III decarboxylase-like protein	0	0	0	0	0	0	0	0	0	167.378	0	85.9262	0
Uroporphyrinogen-III methyltransferase (EC 2.1.1.107)	0	51.1944	24.345	0	0	0	0	1.183777	0.602554	0	0	0	0.215578
V-type ATP synthase subunit I (EC 3.6.3.14)	0	3.43712	1.35694	0	0	0	0	0	0	0	0	0	0
Valyl-tRNA synthetase (EC 6.1.1.9)	0.454207	10.53887	5.116793	0	0	0	0	356.9169	175.7908	38.0946	1.9849	24.304029	0.962629
Vibriolysin, extracellular zinc protease (EC 3.4.24.25)	0	0	35.8943	0	0	0	0	0	0	0	0	0	0
Vitamin-B12 independent methionine synthase family protein	0	12.20161	5.85716	0	0	0	0	0	0	0	0	0	0
Xanthine and CO dehydrogenases maturation factor, XdhC/CoxF family	304.6137	0	71.5458	0	0	0	0	0	0	0	0	0	0.231231
Xanthine dehydrogenase, molybdenum binding subunit (EC 1.17.1.4)	0	23.08889	10.96756	0	0	0	0	0	0	0	0	0	1.103275
Xanthine permease	0	0	0	1460.746	0	814.7104	0	0	0	0	0	0	0
Xanthine phosphoribosyltransferase (EC 2.4.2.22)	0	0	0	0	0	0	0	0	0	0	115.57	56.2403	0
Xanthine/uracil/thiamine/ascorbate permease family protein	2208.25	0	1158.75	0	0	0	0	0	0	0	0	0	4.926606
Xylose ABC transporter, permease protein XylH	0	0	0	28.701	0	16.6864	0	0	0	0	0	0	0
Xyloside transporter XynT	0	0	0	0	0	0	85.6396	0	35.0799	0	0	0	0
Xylulose-5-phosphate phosphoketolase (EC 4.1.2.9) @ Fructose-6-phosphate phosphoketolase (EC 4.1.2.22)	0	0	0	0	47.9862	20.1641	0	0	0	0	0	0	0
YceI family protein	0	0	0	0	31.0911	13.2849	0	0	0	0	0	0	0
YceI like family protein	0	0	0	0	0	0	0	0	0	14.3327	0	9.42042	0
YehQ protein	0.193643	0	0.101558	0	0	0	0	0	0	0	0	0	0
YihE protein, a ser/thr kinase implicated in LPS synthesis and Cpx signalling	0	0	0	0	13.4014	5.61	0	0	0	0	0	0	0
YlxP-like protein	0	0	0	0	0	0	0	321.75	163.794	0	0	0	0
YoeB toxin protein	1033.28	0	543.665	0	0	0	0	0	0	0	0	0	0
YpfJ protein, zinc metalloprotease superfamily	0	1319.365	627.14	0	0	0	0	0	0	0	0	0	0.159308
YwoG	12.7935	0	0	0	0	0	0	0	0	0	0	0	0
Zinc ABC transporter, inner membrane permease protein ZnuB	0.313431	0	0.164382	38.8779	0	22.5833	0	0	0	0	0	0	0
Zinc-finger protein	0	0	0	0	0	0	0	421.623	214.753	0	0	0	0
Zinc-regulated outer membrane receptor	27.7323	0	14.48173	0	0	0	0	0	0	0	0	0	0.734769
Zn-dependent hydrolase	0.390654	0	0.204882	0	0	0	0	0	0	0	0	0	0
Zn-finger protein	0	0	0	0	1144.28	970.857	0	0	0	0	0	0	0
