# Supplementary material for: Fungal and Prokaryotic Activities in the Marine Subsurface Biosphere at Peru Margin and Canterbury Basin Inferred from RNA-Based Analyses and Microscopy
Source: Front Microbiol. 2016 Jun 9;7:846. doi: 10.3389/fmicb.2016.00846 (PMC4899926; doi:10.3389/fmicb.2016.00846)
Supplement: Table S4 — Profile of fungal metabolic activities in our 345.50 mbsf Canterbury Basin metatranscriptome as revealed by the distribution of contigs across the “biological functions” category as detected by Gene Ontology (GO) term analysis at the GO level 4. [file Table_4.PDF]

GO	#Seqs
metabolic process	206
oxidation-reduction process	110
cellular process	89
cellular metabolic process	71
signal transduction	68
transport	67
primary metabolic process	64
single-organism metabolic process	54
regulation of cellular process	49
regulation of transcription, DNA-templated	44
organic substance metabolic process	41
obsolete ATP catabolic process	36
translation	35
transmembrane transport	27
proteolysis	26
cellular amino acid biosynthetic process	25
protein folding	24
small molecule metabolic process	24
tricarboxylic acid cycle	23
organonitrogen compound metabolic process	23
phosphorylation	22
response to stress	21
transcription, DNA-templated	21
single-organism cellular process	21
glycolytic process	20
nitrogen compound metabolic process	19
protein metabolic process	18
cellular response to stimulus	18
phosphorelay signal transduction system	18
cellular amino acid metabolic process	18
cellular macromolecule metabolic process	17
obsolete GTP catabolic process	16
electron transport chain	15
organic substance biosynthetic process	15
intracellular signal transduction	14
nucleobase-containing small molecule metabolic process	13
cellular biosynthetic process	13
carbohydrate metabolic process	13
biosynthetic process	13
translational elongation	12
ATP synthesis coupled proton transport	12
phosphate-containing compound metabolic process	12
nucleobase-containing compound metabolic process	12
ATP hydrolysis coupled proton transport	12
generation of precursor metabolites and energy	11
DNA metabolic process	11
DNA recombination	11
cellular response to iron ion starvation	10
response to oxidative stress	10
leucine biosynthetic process	10
one-carbon metabolic process	10
pathogenesis	10
cellular response to oxidative stress	9

signal transduction by protein phosphorylation	9
DNA integration	9
isocitrate metabolic process	9
conidium formation	9
cellular aromatic compound metabolic process	8
response to heat	8
ferricrocin biosynthetic process	8
DNA topological change	8
methionine biosynthetic process	8
gluconeogenesis	8
filamentous growth of a population of unicellular organisms in response to biotic stimulus	8
DNA-templated transcription, initiation	7
cellular response to DNA damage stimulus	7
nucleotide biosynthetic process	7
tRNA aminoacylation	7
induction by symbiont of host defense response	7
SOS response	7
chromosome segregation	7
response to stimulus	7
single-organism process	7
branched-chain amino acid biosynthetic process	7
tRNA aminoacylation for protein translation	7
single-organism transport	7
mycelium development	7
macromolecule metabolic process	7
entry into host	6
fatty acid biosynthetic process	6
cation transport	6
proton transport	6
arginine biosynthetic process	6
glucose metabolic process	6
fumarate metabolic process	6
leucine catabolic process	6
RNA processing	6
metal ion transport	6
DNA repair	6
glutamate biosynthetic process	6
extracellular polysaccharide biosynthetic process	6
DNA replication	5
isoleucine biosynthetic process	5
penicillin biosynthetic process	5
lipid metabolic process	5
organic cyclic compound metabolic process	5
angiogenesis	5
purine nucleotide biosynthetic process	5
organic cyclic compound biosynthetic process	5
cyclic nucleotide biosynthetic process	5
regulation of intracellular pH	5
cell redox homeostasis	5
transposition, DNA-mediated	5
cellular response to stress	5
positive regulation of conidium formation	5
RNA-dependent DNA replication	5
intracellular sequestering of iron ion	5

emericellamide biosynthetic process	5
'de novo' IMP biosynthetic process	5
aromatic amino acid family biosynthetic process	5
cell division	5
histidine catabolic process to glutamate and formate	5
chorismate biosynthetic process	5
alpha-amino acid metabolic process	5
negative regulation of cell adhesion involved in substrate-bound cell migration	5
histidine catabolic process to glutamate and formamide	5
cellular protein metabolic process	5
carboxylic acid metabolic process	5
pigment biosynthetic process	5
sulfate transport	4
tryptophan metabolic process	4
cellular response to drug	4
ergosterol biosynthetic process	4
response to salt stress	4
amino acid transport	4
valine biosynthetic process	4
hyphal growth	4
protein refolding	4
glutamate metabolic process	4
pentose-phosphate shunt	4
tRNA wobble uridine modification	4
purine-containing compound metabolic process	4
histidine catabolic process	4
cellular nitrogen compound metabolic process	4
heterocycle metabolic process	4
protein transport	4
protein autophosphorylation	4
urea catabolic process	4
secondary metabolite biosynthetic process	4
protein phosphorylation	4
ncRNA metabolic process	4
protein secretion	4
gene expression	4
aromatic amino acid family metabolic process	4
nucleotide metabolic process	4
ketone body catabolic process	4
protein processing	4
response to sucrose	4
chemotaxis	4
cellular response to organic substance	4
lysine biosynthetic process	4
filamentous growth of a population of unicellular organisms	4
cysteine metabolic process	4
response to drug	4
biological regulation	3
cellular response to farnesol	3
regulation of nitrogen utilization	3
sulfur compound metabolic process	3
aerobic respiration	3
ATP biosynthetic process	3
anisotropic cell growth	3

oxidative phosphorylation	3
DNA ligation involved in DNA repair	3
photosynthesis, light reaction	3
purine-containing compound biosynthetic process	3
ethanol oxidation	3
glutamine family amino acid metabolic process	3
RNA metabolic process	3
protein catabolic process	3
response to chemical	3
glutamine metabolic process	3
spore germination	3
ATP metabolic process	3
pilus assembly	3
glycine catabolic process	3
pyruvate metabolic process	3
nucleotide-excision repair	3
methionine transport	3
monocarboxylic acid metabolic process	3
alpha-amino acid catabolic process	3
cellular response to hydrogen peroxide	3
heterocycle biosynthetic process	3
glutamine biosynthetic process	3
potassium ion transport	3
virion assembly	3
ion transport	3
response to light stimulus	3
protein peptidyl-prolyl isomerization	3
'de novo' AMP biosynthetic process	3
cellular response to osmotic stress	3
pyrimidine nucleotide biosynthetic process	3
tetrapyrrole biosynthetic process	3
phosphatidylinositol phosphorylation	3
carbon utilization	3
N',N'',N'''-triacetylfusarinine C biosynthetic process	3
nitrogen fixation	3
protein targeting to mitochondrion	2
protein targeting	2
protein import	2
alcohol metabolic process	2
establishment of cell polarity	2
cysteine biosynthetic process from serine	2
pteridine-containing compound metabolic process	2
chaperone mediated protein folding requiring cofactor	2
lipopolysaccharide transport	2
establishment or maintenance of cell polarity	2
threonine catabolic process	2
fatty acid beta-oxidation using acyl-CoA dehydrogenase	2
dephosphorylation	2
Mo-molybdopterin cofactor biosynthetic process	2
regulation of nitrogen compound metabolic process	2
bile acid metabolic process	2
ammonia assimilation cycle	2
3,4-dihydroxybenzoate catabolic process	2
siderophore biosynthetic process	2

cellular catabolic process	2
response to abiotic stimulus	2
antibiotic biosynthetic process	2
carbohydrate transport	2
thiamine metabolic process	2
cilium or flagellum-dependent cell motility	2
inositol phosphate dephosphorylation	2
tRNA metabolic process	2
pyrimidine-containing compound metabolic process	2
response to oxygen-containing compound	2
cellular heat acclimation	2
single-organism carbohydrate metabolic process	2
regulation of catalytic activity	2
porphyrin-containing compound biosynthetic process	2
lipid biosynthetic process	2
cellular component organization	2
protein targeting to membrane	2
interaction with host	2
alanyl-tRNA aminoacylation	2
phospholipid biosynthetic process	2
organonitrogen compound biosynthetic process	2
chromosome organization	2
organophosphate metabolic process	2
riboflavin biosynthetic process	2
threonine biosynthetic process	2
propionate catabolic process, 2-methylcitrate cycle	2
L-serine metabolic process	2
nitrogen utilization	2
response to cadmium ion	2
response to starvation	2
L-lysine catabolic process to acetate	2
S-adenosylmethionine biosynthetic process	2
single-species biofilm formation on inanimate substrate	2
single-organism developmental process	2
cellular response to alkaline pH	2
isoprenoid biosynthetic process	2
adenine catabolic process	2
single-organism biosynthetic process	2
pseudouridine synthesis	2
glycine decarboxylation via glycine cleavage system	2
L-serine biosynthetic process	2
'de novo' CTP biosynthetic process	2
negative regulation of transcription, DNA-templated	2
response to toxic substance	2
cellular respiration	2
post-embryonic development	2
diaminopimelate metabolic process	2
glyoxylate cycle	2
copper ion transport	2
response to fructose	2
mismatch repair	2
organic substance catabolic process	2
fatty acid beta-oxidation	2
alpha-amino acid biosynthetic process	2

response to hydrogen peroxide	2
nonribosomal peptide biosynthetic process	2
hypoxanthine salvage	2
osmosensory signaling via phosphorelay pathway	2
cellular carbohydrate metabolic process	2
DNA-dependent DNA replication	2
multicellular organismal development	2
protein homooligomerization	2
translational initiation	2
fungal-type cell wall organization	2
Golgi organization	2
peptide biosynthetic process	2
cell cycle	2
positive regulation of translation	2
response to organic substance	2
carboxylic acid biosynthetic process	2
biotin biosynthetic process	2
purine ribonucleoside monophosphate biosynthetic process	2
intracellular protein transmembrane transport	2
glycerol metabolic process	2
methylation	2
oxalate metabolic process	2
glutathione metabolic process	2
DNA-templated transcription, termination	2
secondary metabolic process	2
single-organism catabolic process	1
endoplasmic reticulum unfolded protein response	1
retinal cell programmed cell death	1
glycosphingolipid metabolic process	1
estrogen metabolic process	1
proteolysis involved in cellular protein catabolic process	1
response to wounding	1
phosphoenolpyruvate-dependent sugar phosphotransferase system	1
cellular response to mechanical stimulus	1
ion transmembrane transport	1
sulfur compound biosynthetic process	1
mitotic spindle organization	1
blood vessel remodeling	1
L-aspartate transport	1
selenium compound metabolic process	1
cysteine biosynthetic process via cystathionine	1
positive regulation of release of sequestered calcium ion into cytosol	1
B cell apoptotic process	1
chondroitin sulfate metabolic process	1
tetrahydrofolate biosynthetic process	1
hydrogen peroxide metabolic process	1
regulation of conidium formation	1
rRNA base methylation	1
tRNA processing	1
D-serine metabolic process	1
siderophore transmembrane transport	1
ammonium transport	1
carbohydrate biosynthetic process	1
cellular component biogenesis	1

branched-chain amino acid catabolic process	1
protein N-linked glycosylation via asparagine	1
cellular response to nitrosative stress	1
siderophore transport	1
oxygen transport	1
chlorophyll catabolic process	1
intracellular pH elevation	1
cytochrome complex assembly	1
sporulation	1
glycerophospholipid metabolic process	1
positive regulation of B cell apoptotic process	1
fertilization	1
establishment of protein localization to organelle	1
negative regulation of sterigmatocystin biosynthetic process	1
sodium ion transport	1
mannitol metabolic process	1
bicarbonate transport	1
regulation of biological process	1
negative regulation of fibroblast proliferation	1
2-oxoglutarate metabolic process	1
anatomical structure development	1
iron ion homeostasis	1
B cell homeostatic proliferation	1
anatomical structure formation involved in morphogenesis	1
malonyl-CoA biosynthetic process	1
response to food	1
L-phenylalanine catabolic process	1
response to selenium ion	1
response to glucose	1
D-glucarate catabolic process	1
glutamine catabolic process	1
purine nucleobase biosynthetic process	1
glycine metabolic process	1
methylation-dependent chromatin silencing	1
response to acid chemical	1
iron assimilation	1
ovarian follicle development	1
positive regulation of apoptotic process involved in mammary gland involution	1
seryl-tRNA aminoacylation	1
neuron migration	1
arginine catabolic process	1
homeostasis of number of cells within a tissue	1
RNA methylation	1
positive regulation of flavonoid biosynthetic process	1
phosphate ion transmembrane transport	1
ketone biosynthetic process	1
response to xenobiotic stimulus	1
signal peptide processing	1
protein glutathionylation	1
nuclear fragmentation involved in apoptotic nuclear change	1
activation of cysteine-type endopeptidase activity involved in apoptotic process by cytochrome c	1
sulfur amino acid metabolic process	1
cellular protein complex assembly	1
unsaturated fatty acid biosynthetic process	1

jasmonic acid biosynthetic process	1
response to gamma radiation	1
asexual sporulation resulting in formation of a cellular spore	1
nitrate assimilation	1
fatty acid catabolic process	1
pyrimidine nucleobase biosynthetic process	1
trehalose biosynthetic process	1
phosphorus metabolic process	1
regulation of insulin secretion	1
Sertoli cell proliferation	1
inositol biosynthetic process	1
macromolecule biosynthetic process	1
anthocyanin-containing compound biosynthetic process	1
serine family amino acid metabolic process	1
calcium ion transport	1
carbohydrate derivative metabolic process	1
methanogenesis	1
regulation of protein homodimerization activity	1
acetate catabolic process	1
cytoplasmic transport	1
kidney development	1
selenocysteinyl-tRNA(Sec) biosynthetic process	1
protein secretion by the type III secretion system	1
negative regulation of cellular process	1
heme metabolic process	1
histidine biosynthetic process	1
androgen metabolic process	1
folic acid-containing compound metabolic process	1
oxidoreduction coenzyme metabolic process	1
sphingolipid biosynthetic process	1
cell differentiation	1
selenocysteine biosynthetic process	1
regulation of fatty acid biosynthetic process	1
positive regulation of neuron apoptotic process	1
glycerol ether metabolic process	1
positive regulation of filamentous growth of a population of unicellular organisms in response to biotic stimulus	1
regulation of mammary gland epithelial cell proliferation	1
regulation of multicellular organism growth	1
glyoxylate catabolic process	1
carbon catabolite activation of transcription	1
alpha-linolenic acid metabolic process	1
cellular polysaccharide biosynthetic process	1
GMP biosynthetic process	1
nucleobase-containing small molecule interconversion	1
cellular hyperosmotic response	1
arginine biosynthetic process via ornithine	1
cytoskeleton organization	1
dolichol-linked oligosaccharide biosynthetic process	1
DNA methylation on adenine	1
response to axon injury	1
sulfate assimilation	1
very long-chain fatty acid metabolic process	1
protein insertion into mitochondrial membrane involved in apoptotic signaling pathway	1
cellular calcium ion homeostasis	1

coenzyme biosynthetic process	1
endocrocin biosynthetic process	1
pigment metabolic process involved in developmental pigmentation	1
filamentous growth	1
transition metal ion transport	1
phosphatidylcholine biosynthetic process	1
regulation of arginine biosynthetic process via ornithine	1
release of matrix enzymes from mitochondria	1
tRNA threonylcarbamoyladenosine modification	1
cellular response to UV	1
determination of adult lifespan	1
chromatin silencing	1
apoptotic DNA fragmentation	1
regulation of signal transduction	1
response to hypoxia	1
obsolete cleavage of lamin involved in execution phase of apoptosis	1
hydrogen sulfide biosynthetic process	1
glycerol-3-phosphate catabolic process	1
lysyl-tRNA aminoacylation	1
tetrahydrofolylpolyglutamate biosynthetic process	1
response to misfolded protein	1
isoleucine catabolic process	1
tyrosyl-tRNA aminoacylation	1
proteasome core complex assembly	1
histidine metabolic process	1
regulation of hydrogen peroxide metabolic process	1
chondrocyte development	1
B cell homeostasis	1
peptidyl-tyrosine dephosphorylation	1
ectopic germ cell programmed cell death	1
nucleic acid phosphodiester bond hydrolysis	1
response to biotic stimulus	1
superoxide metabolic process	1
protein glycosylation	1
ureide catabolic process	1
mRNA catabolic process	1
organic hydroxy compound metabolic process	1
dihydrofolate biosynthetic process	1
histone H3-K9 methylation	1
steroid metabolic process	1
response to temperature stimulus	1
mannan catabolic process	1
retina development in camera-type eye	1
transsulfuration	1
endochondral ossification	1
acetate metabolic process	1
regulation of transport	1
systemic acquired resistance, salicylic acid mediated signaling pathway	1
hyperosmotic response	1
cellular response to cation stress	1
G2/M transition of mitotic cell cycle	1
reductive pentose-phosphate cycle	1
NADP biosynthetic process	1
post-embryonic camera-type eye morphogenesis	1

cellular response to phosphate starvation	1
ecdysone metabolic process	1
succinate transmembrane transport	1
negative regulation of peptidyl-serine phosphorylation	1
red or far-red light signaling pathway	1
response to cold	1
phagocytosis	1
cellular component organization or biogenesis	1
RNA modification	1
mating pheromone secretion involved in conjugation with cellular fusion	1
development of secondary sexual characteristics	1
tRNA thio-modification	1
long-chain fatty acid catabolic process	1
aromatic compound biosynthetic process	1
peptide pheromone export	1
dicarboxylic acid transport	1
mitochondrial fusion	1
nitric oxide catabolic process	1
'de novo' protein folding	1
valine catabolic process	1
G-protein coupled receptor signaling pathway	1
release of cytochrome c from mitochondria	1
urea metabolic process	1
carnitine metabolic process, CoA-linked	1
dipeptide transport	1
cellular macromolecule biosynthetic process	1
positive regulation of endoplasmic reticulum unfolded protein response	1
phosphate ion transport	1
negative regulation of fatty acid oxidation	1
ribosome biogenesis	1
SRP-dependent cotranslational protein targeting to membrane	1
movement of cell or subcellular component	1
lysine metabolic process	1
protein tetramerization	1
negative regulation of endoplasmic reticulum calcium ion concentration	1
vagina development	1
STAT protein import into nucleus	1
translational termination	1
mitochondrial fragmentation involved in apoptotic process	1
regulation of epithelial cell differentiation	1
mitochondrial translation	1
T cell homeostatic proliferation	1
ubiquinone biosynthetic process	1
ethanol metabolic process	1
negative regulation of neuron apoptotic process	1
monovalent inorganic cation transport	1
acyl-CoA metabolic process	1
fatty acid metabolic process	1
B cell negative selection	1
nucleobase-containing compound biosynthetic process	1
glucan catabolic process	1
purine nucleobase metabolic process	1
proteasomal protein catabolic process	1
positive regulation of anthocyanin metabolic process	1

spermatid differentiation	1
fatty acid beta-oxidation using acyl-CoA oxidase	1
macromolecule modification	1
temperature homeostasis	1
positive regulation of protein oligomerization	1
cellular response to heat	1
ribosomal small subunit biogenesis	1
positive regulation of developmental pigmentation	1
gibberellic acid mediated signaling pathway	1
embryo development ending in seed dormancy	1
positive regulation of cell proliferation	1
branched-chain amino acid metabolic process	1
coumarin biosynthetic process	1
regulation of protein heterodimerization activity	1
sucrose induced translational repression	1
adhesion of symbiont to host	1
L-methionine biosynthetic process from homoserine via O-acetyl-L-homoserine and cystathionine	1
formaldehyde catabolic process	1
retinal cell apoptotic process	1
mitotic spindle elongation	1
tyrosine biosynthetic process from chorismate via 4-hydroxyphenylpyruvate	1
acetate biosynthetic process	1
pyrimidine-containing compound biosynthetic process	1
removal of superoxide radicals	1
heme biosynthetic process	1
L-phenylalanine biosynthetic process from chorismate via phenylpyruvate	1
multi-organism process	1
protein heterooligomerization	1
positive regulation of cell death	1
ammonium transmembrane transport	1
carbamoyl phosphate biosynthetic process	1
ubiquitin-dependent protein catabolic process	1
carnitine catabolic process	1
cerebral cortex development	1
GDP-mannose metabolic process	1
acetyl-CoA biosynthetic process from pyruvate	1
lipoate metabolic process	1
regulation of metabolic process	1
rRNA processing	1
cellular nitrogen compound biosynthetic process	1
positive regulation of intrinsic apoptotic signaling pathway	1
aspartate biosynthetic process	1
sucrose catabolic process	1
circadian rhythm	1
mitotic nuclear division	1
germ cell development	1
isopentenyl diphosphate biosynthetic process, methylerythritol 4-phosphate pathway	1
cell septum assembly	1
water transport	1
positive regulation of JAK-STAT cascade	1
protein homotetramerization	1
response to defense-related host nitric oxide production	1
regulation of gene expression	1
iron incorporation into metallo-sulfur cluster	1

gravitropism	1
alkanesulfonate catabolic process	1
pyridine-containing compound biosynthetic process	1
galactonate metabolic process	1
nucleic acid metabolic process	1
asperthecin biosynthetic process	1
embryonic digit morphogenesis	1
hydrogen peroxide catabolic process	1
pyruvate catabolic process	1
glutathione biosynthetic process	1
regulation of translational fidelity	1
root hair elongation	1
hypothalamus development	1
cellular response to starvation	1
regulation of cell cycle	1
positive regulation of multicellular organism growth	1
regulation of intracellular steroid hormone receptor signaling pathway	1
intrinsic apoptotic signaling pathway in response to endoplasmic reticulum stress	1
glycerol catabolic process	1
chloroplast organization	1
cell adhesion	1
pyridine nucleotide biosynthetic process	1
'de novo' pyrimidine nucleobase biosynthetic process	1
fumarate transport	1
tryptophan biosynthetic process	1
intron homing	1
propionate metabolic process, methylcitrate cycle	1
nucleotide phosphorylation	1
glycerol-3-phosphate biosynthetic process	1
arginine metabolic process	1
leucyl-tRNA aminoacylation	1
positive regulation of release of cytochrome c from mitochondria	1
cellular response to insulin stimulus	1
monosaccharide metabolic process	1
neuron apoptotic process	1
tripeptide transport	1
establishment or maintenance of transmembrane electrochemical gradient	1
proline biosynthetic process	1
glutaminyl-tRNAGln biosynthesis via transamidation	1
response to high light intensity	1
methylglyoxal catabolic process to D-lactate via S-lactoyl-glutathione	1
serine transport	1
regulation of cholesterol metabolic process	1
peptidoglycan catabolic process	1
glycine biosynthetic process	1
vitamin metabolic process	1
positive regulation of glycine hydroxymethyltransferase activity	1
regulation of mitochondrial membrane potential	1
cofactor metabolic process	1
negative regulation of fatty acid biosynthetic process	1
nucleotide-sugar metabolic process	1
L-proline biosynthetic process	1
post-translational protein modification	1
response to red or far red light	1

filamentous growth of a population of unicellular organisms in response to starvation	1
adenosine biosynthetic process	1
protein complex assembly	1
acetaldehyde catabolic process	1
glycerophosphate shuttle	1
lysine biosynthetic process via diaminopimelate	1
transformed cell apoptotic process	1
cofactor biosynthetic process	1
GMP metabolic process	1
trehalose catabolic process	1
reproductive process	1
myeloid cell homeostasis	1
regulation of lipid metabolic process	1
reproductive structure development	1
regulation of nuclear division	1
negative regulation of protein binding	1
posttranscriptional gene silencing	1
iron-sulfur cluster assembly	1
mRNA splicing, via spliceosome	1
cell-matrix adhesion	1
malate metabolic process	1
nucleoside triphosphate biosynthetic process	1
multicellular organismal process	1
aerobic electron transport chain	1
