# Supplementary material for: Fungal and Prokaryotic Activities in the Marine Subsurface Biosphere at Peru Margin and Canterbury Basin Inferred from RNA-Based Analyses and Microscopy
Source: Front Microbiol. 2016 Jun 9;7:846. doi: 10.3389/fmicb.2016.00846 (PMC4899926; doi:10.3389/fmicb.2016.00846)
Supplement: Table S5 — Profile of fungal metabolic activities in our 345.50 mbsf Canterbury Basin metatranscriptome as revealed by the distribution of contigs across the “molecular functions” category as detected by Gene Ontology (GO) term analysis at the GO level 4. [file Table_5.PDF]

GO	#Seqs
catalytic activity	288
ATP binding	146
oxidoreductase activity	131
transferase activity	107
nucleotide binding	107
binding	97
hydrolase activity	96
metal ion binding	70
nucleoside-triphosphatase activity	67
heterocyclic compound binding	59
organic cyclic compound binding	59
DNA binding	54
ligase activity	44
ion binding	43
transporter activity	34
ATPase activity	34
lyase activity	33
signal transducer activity	30
flavin adenine dinucleotide binding	27
transferase activity, transferring phosphorus-containing groups	26
transcription factor activity, sequence-specific DNA binding	24
peptidase activity	23
magnesium ion binding	23
kinase activity	22
NAD binding	22
electron carrier activity	22
oxidoreductase activity, acting on the CH-CH group of donors	21
zinc ion binding	20
iron ion binding	19
oxidoreductase activity, acting on the aldehyde or oxo group of donors, NAD or NADP as acceptor	19
GTP binding	18
structural constituent of ribosome	18
pyridoxal phosphate binding	17
nucleic acid binding	17
protein binding	16
transferase activity, transferring acyl groups	16
unfolded protein binding	16
GTPase activity	16
RNA binding	15
4 iron, 4 sulfur cluster binding	15
iron-sulfur cluster binding	14
proton-transporting ATP synthase activity, rotational mechanism	14
helicase activity	13
coenzyme binding	12
translation elongation factor activity	12
heme binding	12
isomerase activity	11
proton-transporting ATPase activity, rotational mechanism	11
ATPase activity, coupled to transmembrane movement of substances	11
peroxidase activity	10
aminoacyl-tRNA ligase activity	10
oxidoreductase activity, acting on the CH-OH group of donors, NAD or NADP as acceptor	10
NADP binding	9
small molecule binding	9
anion binding	9
phosphorelay sensor kinase activity	9
phosphorelay response regulator activity	9
cation-transporting ATPase activity	9

isocitrate dehydrogenase (NADP+) activity	9
acyl-CoA dehydrogenase activity	8
ligase activity, forming aminoacyl-tRNA and related compounds	8
endonuclease activity	8
protein histidine kinase activity	8
identical protein binding	8
DNA topoisomerase type II (ATP-hydrolyzing) activity	7
biotin carboxylase activity	7
sigma factor activity	7
transition metal ion binding	7
transferase activity, transferring acyl groups other than amino-acyl groups	7
3-isopropylmalate dehydratase activity	7
phosphopyruvate hydratase activity	7
fumarate hydratase activity	7
amino acid binding	7
high molecular weight kininogen binding	6
rRNA binding	6
oxidoreductase activity, acting on the CH-NH2 group of donors, NAD or NADP as acceptor	6
cobalamin binding	6
glucose-1-phosphate thymidylyltransferase activity	6
purine ribonucleoside binding	6
hydrolase activity, acting on acid anhydrides	6
phosphorus-oxygen lyase activity	5
S-(hydroxymethyl)glutathione dehydrogenase activity	5
transposase activity	5
hydrolase activity, acting on carbon-nitrogen (but not peptide) bonds	5
purine ribonucleotide binding	5
purine ribonucleoside triphosphate binding	5
N-(5-amino-5-carboxypentanoyl)-L-cysteinyl-D-valine synthase activity	5
copper ion binding	5
glutamate synthase activity	5
RNA-directed DNA polymerase activity	5
MHC class I protein binding	5
inositol monophosphate phosphatase activity	5
peptidase activity, acting on L-amino acid peptides	5
nucleotidyltransferase activity	5
transferase activity, transferring glycosyl groups	5
protein transporter activity	4
acetyl-CoA C-acetyltransferase activity	4
cytochrome-c oxidase activity	4
glutamate synthase (NADH) activity	4
NADH dehydrogenase (ubiquinone) activity	4
methylmalonyl-CoA mutase activity	4
3-oxoacid CoA-transferase activity	4
FMN binding	4
adenosylhomocysteinase activity	4
quinone binding	4
methyltransferase activity	4
glutamate synthase (NADPH) activity	4
phosphoenolpyruvate carboxykinase activity	4
oxidoreductase activity, acting on the CH-NH2 group of donors	4
urocanate hydratase activity	4
glutamate synthase activity, NAD(P)H as acceptor	4
ammonia-lyase activity	4
dihydroxy-acid dehydratase activity	4
electron-transferring-flavoprotein dehydrogenase activity	4
sulfate transmembrane-transporting ATPase activity	4
tRNA binding	4
metallopeptidase activity	4

exopeptidase activity	4
2 iron, 2 sulfur cluster binding	4
transaminase activity	4
oxidoreductase activity, acting on paired donors, with incorporation or reduction of molecular oxygen	4
methylcrotonoyl-CoA carboxylase activity	4
hydrolase activity, acting on acid anhydrides, catalyzing transmembrane movement of substances	3
pyruvate carboxylase activity	3
hydrolase activity, acting on glycosyl bonds	3
protein homodimerization activity	3
protein disulfide oxidoreductase activity	3
5-methyltetrahydropteroyltriglutamate-homocysteine S-methyltransferase activity	3
succinate dehydrogenase (ubiquinone) activity	3
transferase activity, transferring alkyl or aryl (other than methyl) groups	3
glyceraldehyde-3-phosphate dehydrogenase (NAD+) (phosphorylating) activity	3
methionine synthase activity	3
inositol monophosphate 3-phosphatase activity	3
intramolecular transferase activity, phosphotransferases	3
metalloendopeptidase activity	3
glutamate-ammonia ligase activity	3
L-phenylalanine:2-oxoglutarate aminotransferase activity	3
protein kinase activity	3
inositol monophosphate 4-phosphatase activity	3
potassium-transporting ATPase activity	3
cation binding	3
hydrolase activity, acting on acid anhydrides, in phosphorus-containing anhydrides	3
adenylosuccinate synthase activity	3
recombinase activity	3
ATP-dependent helicase activity	3
glycine dehydrogenase (decarboxylating) activity	3
excinuclease ABC activity	3
purine nucleotide binding	3
damaged DNA binding	3
receptor activity	3
single-stranded DNA binding	3
glutamate synthase (ferredoxin) activity	3
transferase activity, transferring acyl groups, acyl groups converted into alkyl on transfer	3
lipid binding	3
inositol monophosphate 1-phosphatase activity	3
serine-type peptidase activity	3
nickel cation binding	3
argininosuccinate synthase activity	3
oxidoreductase activity, acting on CH-OH group of donors	3
sequence-specific DNA binding	3
phosphoric diester hydrolase activity	3
aldehyde-lyase activity	3
NAD+ binding	3
phosphatase activity	3
3-oxoacyl-[acyl-carrier-protein] reductase (NADPH) activity	3
peptidyl-prolyl cis-trans isomerase activity	3
serine-type endopeptidase activity	3
methionine transmembrane transporter activity	3
carbon-nitrogen ligase activity, with glutamine as amido-N-donor	3
DNA ligase (ATP) activity	3
isovaleryl-CoA dehydrogenase activity	3
gluconate 2-dehydrogenase (acceptor) activity	3
transcription factor binding	2
lipopolysaccharide-transporting ATPase activity	2
3-phosphoshikimate 1-carboxyvinyltransferase activity	2
urease activity	2

carboxylic ester hydrolase activity	2
propionyl-CoA carboxylase activity	2
L-serine ammonia-lyase activity	2
glucose-6-phosphate isomerase activity	2
metal ion transmembrane transporter activity	2
endodeoxyribonuclease activity, producing 5'-phosphomonoesters	2
transferase activity, transferring pentosyl groups	2
hydrolase activity, acting on carbon-nitrogen (but not peptide) bonds, in cyclic amides	2
sulfurtransferase activity	2
oxidoreductase activity, acting on a sulfur group of donors, NAD(P) as acceptor	2
oxygen transporter activity	2
S-adenosylmethionine-homocysteine S-methyltransferase activity	2
ATPase activity, coupled	2
palmitoyl-CoA oxidase activity	2
methionine adenosyltransferase activity	2
3-dehydroquinate dehydratase activity	2
hydroxymethyl-, formyl- and related transferase activity	2
phosphoribosylamine-glycine ligase activity	2
adenine deaminase activity	2
histidine phosphotransfer kinase activity	2
shikimate kinase activity	2
hormone activity	2
arginase activity	2
transmembrane transporter activity	2
cysteine synthase activity	2
protein heterodimerization activity	2
2-isopropylmalate synthase activity	2
potassium ion binding	2
ribosome binding	2
transferase activity, transferring one-carbon groups	2
monooxygenase activity	2
nitronate monooxygenase activity	2
ATPase activity, coupled to transmembrane movement of ions, phosphorylative mechanism	2
L-aminoadipate-semialdehyde dehydrogenase activity	2
shikimate 3-dehydrogenase (NADP+) activity	2
protein dimerization activity	2
isocitrate lyase activity	2
NAD(P)+ transhydrogenase (AB-specific) activity	2
peptidoglycan glycosyltransferase activity	2
dihydroneopterin aldolase activity	2
translation initiation factor activity	2
penicillin binding	2
testosterone dehydrogenase (NAD+) activity	2
DNA-dependent ATPase activity	2
L-aspartate:2-oxoglutarate aminotransferase activity	2
mismatched DNA binding	2
phosphotransferase activity, alcohol group as acceptor	2
acetate-CoA ligase activity	2
alanine-tRNA ligase activity	2
oxidoreductase activity, acting on NAD(P)H	2
acid-amino acid ligase activity	2
RNA-dependent ATPase activity	2
structural molecule activity	2
hydrolase activity, acting on carbon-nitrogen (but not peptide) bonds, in linear amides	2
oxidoreductase activity, acting on single donors with incorporation of molecular oxygen	2
phosphoprotein phosphatase activity	2
sulfate transmembrane transporter activity	2
hydrogen ion transmembrane transporter activity	2
formyltetrahydrofolate deformylase activity	2

alcohol dehydrogenase (NAD) activity	2
DNA topoisomerase activity	2
fructose-bisphosphate aldolase activity	2
3-deoxy-7-phosphoheptulonate synthase activity	2
acetyl-CoA carboxylase activity	2
oxygen binding	2
translation release factor activity	2
hydro-lyase activity	2
CTP synthase activity	2
secondary active sulfate transmembrane transporter activity	2
phosphotransferase activity, phosphate group as acceptor	1
N,N-dimethylaniline monooxygenase activity	1
5-dehydro-4-deoxyglucarate dehydratase activity	1
ribosomal small subunit binding	1
succinate transmembrane transporter activity	1
NADP+ binding	1
serine C-palmitoyltransferase activity	1
glycerol-3-phosphate dehydrogenase activity	1
cadmium-exporting ATPase activity	1
acetyl-CoA hydrolase activity	1
phosphoribosylaminoimidazole carboxylase activity	1
trehalose-phosphatase activity	1
arginine decarboxylase activity	1
disulfide oxidoreductase activity	1
O-phospho-L-serine:2-oxoglutarate aminotransferase activity	1
L-leucine:2-oxoglutarate aminotransferase activity	1
N-acetyl-gamma-glutamyl-phosphate reductase activity	1
alpha-galactosidase activity	1
glutathione-disulfide reductase activity	1
formaldehyde transketolase activity	1
3'(2'),5'-bisphosphate nucleotidase activity	1
citrate (Si)-synthase activity	1
long-chain-acyl-CoA dehydrogenase activity	1
FK506 binding	1
lysine-tRNA ligase activity	1
exonuclease activity	1
NADH dehydrogenase (quinone) activity	1
tyrosine-tRNA ligase activity	1
cation transmembrane transporter activity	1
superoxide dismutase activity	1
glutamine-fructose-6-phosphate transaminase (isomerizing) activity	1
[protein-PII] uridylyltransferase activity	1
adenyl ribonucleotide binding	1
malonyl-CoA decarboxylase activity	1
holo-[acyl-carrier-protein] synthase activity	1
protein tyrosine kinase activity	1
haptoglobin binding	1
benzoate-CoA ligase activity	1
heat shock protein binding	1
glycerol-3-phosphate dehydrogenase [NAD+] activity	1
phospholipase activity	1
acetyl-CoA C-acyltransferase activity	1
cobalamin-transporting ATPase activity	1
glutamate dehydrogenase (NADP+) activity	1
homoserine dehydrogenase activity	1
7-beta-hydroxysteroid dehydrogenase (NADP+) activity	1
L,L-diaminopimelate aminotransferase activity	1
methylmalonyl-CoA carboxytransferase activity	1
dihydropteroate synthase activity	1

fatty-acyl-CoA binding	1
pyruvate kinase activity	1
dihydrofolate reductase activity	1
cofactor binding	1
sodium:dicarboxylate symporter activity	1
phosphate ion transmembrane-transporting ATPase activity	1
bile acid:sodium symporter activity	1
succinate-CoA ligase (GDP-forming) activity	1
3-oxoadipyl-CoA thiolase activity	1
cerebroside-sulfatase activity	1
pyrroline-5-carboxylate reductase activity	1
hydrolase activity, acting on ester bonds	1
aminoacyl-tRNA editing activity	1
protein serine/threonine phosphatase activity	1
ATPase activity, coupled to transmembrane movement of ions, rotational mechanism	1
5-(carboxyamino)imidazole ribonucleotide mutase activity	1
phosphoglycerate kinase activity	1
FMN reductase activity	1
enzyme regulator activity	1
GDP-mannose 4,6-dehydratase activity	1
dicarboxylic acid transmembrane transporter activity	1
cystathionine beta-synthase activity	1
phenylalanine-tRNA ligase activity	1
3'-nucleotidase activity	1
inorganic phosphate transmembrane transporter activity	1
hydrogen-exporting ATPase activity, phosphorylative mechanism	1
malate synthase activity	1
organic phosphonate transmembrane-transporting ATPase activity	1
UDP-glucose 4-epimerase activity	1
choline dehydrogenase activity	1
3,4-dihydroxy-2-butanone-4-phosphate synthase activity	1
chloramphenicol O-acetyltransferase activity	1
NAD(P)+ transhydrogenase activity	1
ammonium transmembrane transporter activity	1
glucose-6-phosphate dehydrogenase activity	1
GMP synthase (glutamine-hydrolyzing) activity	1
oxoglutarate dehydrogenase (succinyl-transferring) activity	1
cAMP-dependent protein kinase activity	1
carbonate dehydratase activity	1
galactonate dehydratase activity	1
malate:proton symporter activity	1
acetylglutamate kinase activity	1
allophanate hydrolase activity	1
tartronate-semialdehyde synthase activity	1
site-specific DNA-methyltransferase (adenine-specific) activity	1
chaperone binding	1
acetate CoA-transferase activity	1
RNA methyltransferase activity	1
2-octaprenylphenol hydroxylase activity	1
pyruvate dehydrogenase activity	1
cysteine desulfurase activity	1
protein tyrosine/serine/threonine phosphatase activity	1
guanosine tetraphosphate binding	1
histidinol-phosphate transaminase activity	1
carboxymethylenebutenolidase activity	1
cobalt ion binding	1
double-stranded RNA binding	1
nitric oxide dioxygenase activity	1
laminin binding	1

S-methyltransferase activity	1
N-acetylmuramoyl-L-alanine amidase activity	1
acetylglutamate kinase regulator activity	1
NAD+ kinase activity	1
molybdenum ion binding	1
carboxy-lyase activity	1
phosphoribosylformylglycinamidine cyclo-ligase activity	1
D-lactate dehydrogenase (cytochrome) activity	1
agmatinase activity	1
dol-P-Man:Man(6)GlcNAc(2)-PP-Dol alpha-1,2-mannosyltransferase activity	1
solute:proton antiporter activity	1
UTP:glucose-1-phosphate uridylyltransferase activity	1
RNA-DNA hybrid ribonuclease activity	1
glutamate 5-kinase activity	1
beta-alanine-pyruvate transaminase activity	1
ATPase coupled ion transmembrane transporter activity	1
manganese ion binding	1
CoA-transferase activity	1
oxidoreductase activity, acting on single donors with incorporation of molecular oxygen, incorporation of two atoms of oxygen	1
L-aspartate transmembrane transporter activity	1
ribulose-phosphate 3-epimerase activity	1
sugar:proton symporter activity	1
olfactory receptor activity	1
mannitol 2-dehydrogenase activity	1
carbamoyl-phosphate synthase (glutamine-hydrolyzing) activity	1
protein tyrosine phosphatase activity	1
UMP kinase activity	1
adenosylmethionine-8-amino-7-oxononanoate transaminase activity	1
nickel-transporting ATPase activity	1
malate dehydrogenase (decarboxylating) (NADP+) activity	1
dihydrofolate synthase activity	1
glutathione synthase activity	1
BH3 domain binding	1
phosphoglycerate mutase activity	1
dol-P-Man:Man(8)GlcNAc(2)-PP-Dol alpha-1,2-mannosyltransferase activity	1
calmodulin binding	1
gamma-glutamyltransferase activity	1
hydroxyacylglutathione hydrolase activity	1
leucine-tRNA ligase activity	1
DNA topoisomerase type I activity	1
acyl-CoA hydrolase activity	1
acid phosphatase activity	1
fumarate transmembrane transporter activity	1
lactoylglutathione lyase activity	1
argininosuccinate lyase activity	1
protein-N(PI)-phosphohistidine-sugar phosphotransferase activity	1
phosphopantetheine binding	1
structural constituent of myelin sheath	1
phosphoglycerate dehydrogenase activity	1
serine-tRNA ligase activity	1
carboxyl- or carbamoyltransferase activity	1
S-acyltransferase activity	1
nitrite reductase [NAD(P)H] activity	1
succinate-semialdehyde dehydrogenase (NAD+) activity	1
pyrophosphatase activity	1
calmodulin-dependent protein kinase activity	1
carbon-oxygen lyase activity	1
polyribonucleotide nucleotidyltransferase activity	1
double-stranded DNA binding	1

formate dehydrogenase (NAD+) activity	1
catalase activity	1
glutaminyl-tRNA synthase (glutamine-hydrolyzing) activity	1
estradiol 17-beta-dehydrogenase activity	1
cytochrome-c peroxidase activity	1
adenylate kinase activity	1
dihydrolipoyl dehydrogenase activity	1
3-carboxy-cis,cis-muconate cycloisomerase activity	1
aromatic-amino-acid:2-oxoglutarate aminotransferase activity	1
3-hydroxybutyrate dehydrogenase activity	1
FMN adenylyltransferase activity	1
transketolase activity	1
UDP-N-acetylmuramate dehydrogenase activity	1
pyruvate dehydrogenase (acetyl-transferring) activity	1
beta-mannosidase activity	1
isoquinoline 1-oxidoreductase activity	1
3'-5'-exoribonuclease activity	1
tripeptide transporter activity	1
heme-copper terminal oxidase activity	1
channel activity	1
adenyl nucleotide binding	1
acryloyl-CoA reductase activity	1
neutral amino acid:sodium symporter activity	1
peroxiredoxin activity	1
DNA-directed RNA polymerase activity	1
4-hydroxyphenylpyruvate dioxygenase activity	1
tartrate dehydrogenase activity	1
succinate-CoA ligase activity	1
protein self-association	1
glutathione binding	1
prolactin receptor binding	1
receptor binding	1
glycerol-3-phosphate dehydrogenase [NAD(P)+] activity	1
urea carboxylase activity	1
allantoate deiminase activity	1
tryptophan synthase activity	1
aspartic-type endopeptidase activity	1
fibronectin binding	1
ribose phosphate diphosphokinase activity	1
porphobilinogen synthase activity	1
ATP citrate synthase activity	1
growth hormone receptor binding	1
thioredoxin-disulfide reductase activity	1
alpha-methylacyl-CoA racemase activity	1
succinyldiaminopimelate transaminase activity	1
pseudouridine synthase activity	1
rRNA (cytosine-N4-)-methyltransferase activity	1
riboflavin kinase activity	1
sucrose alpha-glucosidase activity	1
aspartate carbamoyltransferase activity	1
methylglutaconyl-CoA hydratase activity	1
inorganic diphosphatase activity	1
serine racemase activity	1
aldo-keto reductase (NADP) activity	1
succinate-CoA ligase (ADP-forming) activity	1
nitrate reductase activity	1
G-protein coupled receptor activity	1
maleylacetoacetate isomerase activity	1
glucan 1,6-alpha-glucosidase activity	1

ATP-dependent peptidase activity	1
sedoheptulose-7-phosphate:D-glyceraldehyde-3-phosphate glyceronetransferase activity	1
sulfuric ester hydrolase activity	1
D-amino-acid dehydrogenase activity	1
tetrahydrofolylpolyglutamate synthase activity	1
phosphate acetyltransferase activity	1
selenocysteine lyase activity	1
dipeptide transporter activity	1
phosphoribosylaminoimidazolesuccinocarboxamide synthase activity	1
D-serine ammonia-lyase activity	1
UDP-N-acetylglucosamine 1-carboxyvinyltransferase activity	1
chorismate synthase activity	1
2-amino-4-hydroxy-6-hydroxymethyldihydropteridine diphosphokinase activity	1
aldehyde dehydrogenase (NAD) activity	1
siderophore transmembrane transporter activity	1
thiamine pyrophosphate binding	1
acetoacetyl-CoA reductase activity	1
carbohydrate binding	1
L-tyrosine:2-oxoglutarate aminotransferase activity	1
[isocitrate dehydrogenase (NADP+)] kinase activity	1
xanthine dehydrogenase activity	1
ribose-5-phosphate isomerase activity	1
histidine ammonia-lyase activity	1
nucleotide kinase activity	1
dihydroorotase activity	1
aminoacyl-tRNA hydrolase activity	1
polyamine-transporting ATPase activity	1
ligase activity, forming carbon-nitrogen bonds	1
calcium ion binding	1
formaldehyde dehydrogenase activity	1
ubiquinone binding	1
beta-glucosidase activity	1
oxidoreductase activity, acting on the CH-CH group of donors, NAD or NADP as acceptor	1
ATP-dependent DNA helicase activity	1
aconitate hydratase activity	1
aldehyde dehydrogenase [NAD(P)+] activity	1
malate dehydrogenase (decarboxylating) (NAD+) activity	1
homoserine kinase activity	1
methylisocitrate lyase activity	1
