# Supplementary material for: Fungal and Prokaryotic Activities in the Marine Subsurface Biosphere at Peru Margin and Canterbury Basin Inferred from RNA-Based Analyses and Microscopy
Source: Front Microbiol. 2016 Jun 9;7:846. doi: 10.3389/fmicb.2016.00846 (PMC4899926; doi:10.3389/fmicb.2016.00846)
Supplement: Table S6 — Profile of fungal metabolic activities in our 345.50 mbsf Canterbury Basin metatranscriptome as revealed by the distribution of contigs across the “cellular component” category as detected by Gene Ontology (GO) term analysis at the GO level 4. [file Table_6.PDF]

GO	#Seqs
membrane	100
cytoplasm	80
integral component of membrane	70
plasma membrane	65
mitochondrion	58
intracellular	41
cytosol	30
cytoplasmic part	21
cell surface	18
extracellular region	16
intracellular membrane-bounded organelle	16
membrane part	14
cell part	13
ribosome	13
outer membrane-bounded periplasmic space	12
proton-transporting ATP synthase complex, catalytic core F(1)	11
nucleus	11
mitochondrial nucleoid	9
yeast-form cell wall	8
hyphal cell wall	8
intracellular organelle	7
mitochondrial inner membrane	7
mitochondrial matrix	7
peroxisome	7
3-isopropylmalate dehydratase complex	7
mitochondrial proton-transporting ATP synthase complex	7
tricarboxylic acid cycle enzyme complex	6
intracellular part	6
fungal-type cell wall	6
cell periphery	5
cell outer membrane	5
respiratory chain	5
chloroplast stroma	4
Golgi apparatus	4
cytoplasmic, membrane-bounded vesicle	4
intracellular ribonucleoprotein complex	4
mitochondrial proton-transporting ATP synthase, catalytic core	4
nucleoid	4
chromosome	4
DNA topoisomerase complex (ATP-hydrolyzing)	4
small ribosomal subunit	4

endoplasmic reticulum membrane	3
chloroplast envelope	3
cytosolic small ribosomal subunit	3
mitochondrial part	3
thylakoid	2
acetyl-CoA carboxylase complex	2
cytoskeleton	2
mitochondrial membrane	2
endoplasmic reticulum lumen	2
plasmodesma	2
chloroplast part	2
ATP-binding cassette (ABC) transporter complex	2
phosphopyruvate hydratase complex	2
secretory granule	2
vacuole	2
carbamoyl-phosphate synthase complex	2
peroxisomal matrix	2
mitochondrial electron transfer flavoprotein complex	1
cell cortex	1
proton-transporting ATP synthase complex, coupling factor F(o)	1
extracellular space	1
proton-transporting two-sector ATPase complex, catalytic domain	1
periplasmic space	1
intracellular organelle lumen	1
glycine cleavage complex	1
cytosolic ribosome	1
microtubule associated complex	1
condensed chromosome kinetochore	1
endocytic vesicle lumen	1
bacterial-type flagellum filament	1
vacuolar membrane	1
spindle pole body	1
prefoldin complex	1
cell wall	1
cell septum	1
large ribosomal subunit	1
signal peptidase complex	1
mitochondrial permeability transition pore complex	1
protein complex	1
catalytic step 2 spliceosome	1
extrinsic component of organelle membrane	1
lipid particle	1

haptoglobin-hemoglobin complex	1
chloroplast thylakoid membrane	1
trans-Golgi network	1
mitochondrial oxoglutarate dehydrogenase complex	1
mitochondrial membrane part	1
glycerol-3-phosphate dehydrogenase complex	1
hemoglobin complex	1
Bcl-2 family protein complex	1
mitochondrial envelope	1
nucleolus	1
glutamyl-tRNA(Gln) amidotransferase complex	1
Ndc80 complex	1
signal recognition particle	1
type III protein secretion system complex	1
mitochondrial outer membrane	1
chloroplast thylakoid	1
myelin sheath	1
tubulin complex	1
integral component of mitochondrial inner membrane	1
endoplasmic reticulum	1
